# Error Exponents and *α*-Mutual Information

**DOI:** 10.3390/e23020199

**Published:** 2021-02-05

**Authors:** Sergio Verdú

**Affiliations:** Independent Researcher, Princeton, NJ 08540, USA; verdu@informationtheory.org

**Keywords:** information measures, relative entropy, Rényi divergence, mutual information, α-mutual information, Augustin–Csiszár mutual information, data transmission, error exponents, large deviations

## Abstract

Over the last six decades, the representation of error exponent functions for data transmission through noisy channels at rates below capacity has seen three distinct approaches: (1) Through Gallager’s $E_{0}$ functions (with and without cost constraints); (2) large deviations form, in terms of conditional relative entropy and mutual information; (3) through the α-mutual information and the Augustin–Csiszár mutual information of order α derived from the Rényi divergence. While a fairly complete picture has emerged in the absence of cost constraints, there have remained gaps in the interrelationships between the three approaches in the general case of cost-constrained encoding. Furthermore, no systematic approach has been proposed to solve the attendant optimization problems by exploiting the specific structure of the information functions. This paper closes those gaps and proposes a simple method to maximize Augustin–Csiszár mutual information of order α under cost constraints by means of the maximization of the α-mutual information subject to an exponential average constraint.

## 1. Introduction

### 1.1. Phase 1: The MIT School

The capacity *C* of a stationary memoryless channel is equal to the maximal symbolwise input–output mutual information. Not long after Shannon [1] established this result, Rice [2] observed that, when operating at any encoding rate R<C, there exist codes whose error probability vanishes exponentially with blocklength, with a speed of decay that decreases as *R* approaches *C*. This early observation moved the center of gravity of information theory research towards the quest for the reliability function, a term coined by Shannon [3] to refer to the maximal achievable exponential decay as a function of *R*. The MIT information theory school, and most notably, Elias [4], Feinstein [5], Shannon [3,6], Fano [7], Gallager [8,9], and Shannon, Gallager and Berlekamp [10,11], succeeded in upper/lower bounding the reliability function by the sphere-packing error exponent function and the random coding error exponent function, respectively. Fortunately, these functions coincide for rates between *C* and a certain value, called the critical rate, thereby determining the reliability function in that region. The influential 1968 textbook by Gallager [9] set down the major error exponent results obtained during Phase 1 of research on this topic, including the expurgation technique to improve upon the random coding error exponent lower bound. Two aspects of those early works (and of Dobrushin’s contemporary papers [12,13] on the topic) stand out:

(a) The error exponent functions were expressed as the result of the Karush-Kuhn-Tucker optimization of ad-hoc functions which, unlike mutual information, carried little insight. In particular, during the first phase, center stage is occupied by the parametrized function of the input distribution $P_{X}$ and the random transformation (or "channel”) $P_{Y|X}$,
  (1) $$\begin{array}{c} E_{0}\left(\rho ,P_{X}\right)=-\log\sum_{y\in B}{\left(\sum_{x\in A}P_{X}\left(x\right)P_{Y|X}^{\frac{1}{1+\rho }}\left(y|x\right)\right)}^{1+\rho }\;, \end{array}$$
  introduced by Gallager in [8].
(b) Despite the large-deviations nature of the setup, none of the tools from that then-nascent field (other than the Chernoff bound) found their way to the first phase of the work on error exponents; in particular, relative entropy, introduced by Kullback and Leibler [14], failed to put in an appearance.

To this date, the reliability function remains open for low rates even for the binary symmetric channel, despite a number of refined converse and achievability results (e.g., [15,16,17,18,19,20,21]) obtained since [9]. Our focus in this paper is not on converse/achievability techniques but on the role played by various information measures in the formulation of error exponent results.

### 1.2. Phase 2: Relative Entropy

The second phase of the error exponent research was pioneered by Haroutunian [22] and Blahut [23], who infused the expressions for the error exponent functions with meaning by incorporating relative entropy. The sphere-packing error exponent function corresponding to a random transformation $P_{Y|X}$ is given as

(2) $$E_{\mathrm{sp}}\left(R\right)=\underset{P_{X}}{\sup}\min_{\begin{array}{c} Q_{Y|X}:A\to B\\ I(P_{X},Q_{Y|X})\leq R\;\; \end{array}}D(Q_{Y|X}∥P_{Y|X}\left|P_{X}\right).$$

Roughly speaking, optimal codes of rate R<C incur in errors due to atypical channel behavior, and large deviations establishes that the overwhelmingly most likely such behavior can be explained as if the channel would be supplanted by the one with mutual information bounded by *R* which is closest to the true channel in conditional relative entropy $D(Q_{Y|X}∥P_{Y|X}|P_{X})$. Within the confines of finite-alphabet memoryless channels, this direction opened the possibility of using the combinatorial method of types to obtain refined results robustifying the choice of the optimal code against incomplete knowledge of the channel. The 1981 textbook by Csiszár and Körner [24] summarizes the main results obtained during Phase 2.

### 1.3. Phase 3: Rényi Information Measures

Entropy and relative entropy were generalized by Rényi [25], who introduced the notions of Rényi entropy and Rényi divergence of order α. He arrived at Rényi entropy by relaxing the axioms Shannon proposed in [1], and showed to be satisfied by no measure but entropy. Shortly after [25], Campbell [26] realized the operational role of Rényi entropy in variable-length data compression if the usual average encoding length criterion E[ℓ(c(X))] is replaced by an exponential average $\alpha^{-1}\log E[\exp\left(\alpha \;\ell \left(c\left(X\right)\right)\right]$. Arimoto [27] put forward a generalized conditional entropy inspired by Rényi’s measures (now known as Arimoto-Rényi conditional entropy) and proposed a generalized mutual information by taking the difference between Rényi entropy and the Arimoto-Rényi conditional entropy. The role of the Arimoto-Rényi conditional entropy in the analysis of the error probability of Bayesian *M*-ary hypothesis testing problems has been recently shown in [28], tightening and generalizing a number of results dating back to Fano’s inequality [29].

Phase 3 of the error exponent research was pioneered by Csiszár [30] where he established a connection between Gallager’s $E_{0}$ function and Rényi divergence by means of a Bayesian measure of the discrepancy among a finite collection of distributions introduced by Sibson [31]. Although [31] failed to realize its connection to mutual information, Csiszár [30,32] noticed that it could be viewed as a natural generalization of mutual information. Arimoto [27] also observed that the unconstrained maximization of his generalized mutual information measure with respect to the input distribution coincides with a scaled version of the maximal $E_{0}$ function. This resulted in an extension of the Arimoto-Blahut algorithm useful for the computation of error exponent functions [33] (see also [34]) for finite-alphabet memoryless channels.

Within Haroutunian’s framework [22] applied in the context of the method of types, Poltyrev [35] proposed an alternative to Gallager’s $E_{0}$ function, defined by means of a cumbersome maximization over a reverse random transformation. This measure turned out to coincide (modulo different parametrizations) with another generalized mutual information introduced four years earlier by Augustin in his unpublished thesis [36], by means of a minimization with respect to an output probability measure.

The key contribution in the development of this third phase is Csiszár’s paper [32] where he makes a compelling case for the adoption of Rényi’s information measures in the large deviations analysis of lossless data compression, hypothesis testing and data transmission. Recall that more than two decades earlier, Csiszár [30] had already established the connection of Gallager’s $E_{0}$ function and the generalized mutual information inspired by Sibson [31], which, henceforth, we refer to as the α-mutual information. Therefore, its relevance to the error exponent analysis of error correcting codes had already been established. Incidentally, more recently, another operational role was found for α-mutual information in the context of the large deviations analysis of composite hypothesis testing [37]. In addition to α-mutual information, and always working with discrete alphabets, Csiszár [32] considers the generalized mutual informations due to Arimoto [27], and to Augustin [36], which we refer to as the Augustin–Csiszár mutual information of order α. Csiszár shows that all those three generalizations of mutual information coincide upon their unconstrained maximization with respect to the input distribution. Further relationships among those Rényi-based generalized mutual informations have been obtained in recent years in [38,39,40,41,42,43,44,45]. In [32] the maximal α-mutual information or generalized capacity of order α finds an operational characterization as a generalized cutoff rate–an equivalent way to express the reliability function. This would have been the final word on the topic if it weren’t for its limitation to discrete-alphabet channels, and more importantly, encoding without cost constraints.

### 1.4. Cost Constraints

If the transmitted codebook is cost-constrained, i.e., every codeword $\left(c_{1},\ldots ,c_{n}\right)$ is forced to satisfy $\sum_{i=1}^{n}b\left(c_{i}\right)\leq n\;\theta$ for some nonnegative cost function b(·), then the channel capacity is equal to the input–output mutual information maximized over input probability measures restricted to satisfy E[b(X)]≤θ. Gallager [9] incorporated cost constraints in his treatment of error exponents by generalizing (1) to the function

(3) $$\begin{array}{c} E_{0}\left(\rho ,P_{X},r,\theta \right)=-\log\sum_{y\in B}{\left(\sum_{x\in A}P_{X}\left(x\right)\exp\left(r\;b(x)-r\;\theta \right)P_{Y|X}^{\frac{1}{1+\rho }}\left(y|x\right)\right)}^{1+\rho }\;, \end{array}$$

with which he was able to prove an achievability result invoking Shannon’s random coding technique [1]. Gallager also suggested in the footnote of page 329 of [9] that the converse technique of [10] is amenable to extension to prove a sphere-packing converse based on (3). However, an important limitation is that that technique only applies to constant-composition codes (all codewords have the same empirical distribution). A more powerful converse circumventing that limitation (at least for symmetric channels) was given by [46] also expressing the upper bound on the reliability function by optimizing (3) with respect to ρ, *r* and $P_{X}$. A notable success of the approach based on the optimization of (3) was the determination of the reliability function (for all rates below capacity) of the direct detection photon channel [47].

In contrast, the Phase Two expression (2) for the sphere-packing error exponent for cost-constrained channels is much more natural and similar to the way the expression for channel capacity is impacted by cost constraints, namely we simply constrain the maximization in (2) to satisfy E[b(X)]≤θ. Unfortunately, no general methods to solve the ensuing optimization have been reported.

Once cost constraints are incorporated, the equivalence among the maximal α-mutual information, maximal order-α Augustin–Csiszár mutual information, and maximal Arimoto mutual information of order α breaks down. Of those three alternatives, it is the maximal Augustin–Csiszár mutual information under cost constraints that appears in the error exponent functions. The challenge is that Augustin–Csiszár mutual information is much harder to evaluate, let alone maximize, than α-mutual information. The Phase 3 effort to encompass cost constraints started by Augustin [36] and was continued recently by Nakiboglu [43]. Their focus was to find a way to express (3) in terms of Rényi information measures. Although, as we explain in Item 62, they did not quite succeed, their efforts were instrumental in developing key properties of the Augustin–Csiszár mutual information.

### 1.5. Organization

To enhance readability and ease of reference, the rest of this work is organized in 81 items, grouped into Section 13 and an appendix.

Basic notions and notation (including the key concept of α-response) are collected in Section 2. Unlike much of the literature on the topic, we do not restrict attention to discrete input/output alphabets, nor do we impose any topological structures on them.

The paper is essentially self-contained. Section 3 covers the required background material on relative entropy, Rényi divergence of order α, and their conditional versions, including a key representation of Rényi divergence in terms of relative entropies and a tilted probability measure, and additive decompositions of Rényi divergence involving the α-response.

Section 4 studies the basic properties of α-mutual information and order-α Augustin–Csiszár mutual information. This includes their variational representations in terms of conventional (non-Rényi) information measures such as conditional relative entropy and mutual information, which are particularly simple to show in the main range of interest in applications to error exponents, namely, α∈(0,1).

The interrelationships between α-mutual information and order-α Augustin–Csiszár mutual information are covered in Section 5, which introduces the dual notions of α-adjunct and 〈α〉-adjunct of an input probability measure.

The maximizations with respect to the input distribution of α-mutual information and order-α Augustin–Csiszár mutual information account for their role in the fundamental limits in data transmission through noisy channels. Section 6 gives a brief review of the results in [45] for the maximization of α-mutual information. For Augustin–Csiszár mutual information, Section 7 covers its unconstrained maximization, which coincides with its α-mutual information counterpart. Section 8 proposes an approach to find $C_{\alpha }^{c}\left(\theta \right)$, the maximal Augustin–Csiszár mutual information of order α∈(0,1) subject to E[b(X)]≤θ. Instead of trying to identify directly the input distribution that maximizes Augustin–Csiszár mutual information, the method seeks its 〈α〉-adjunct. This is tantamount to maximizing α-mutual information over a larger set of distributions.

Section 9 shows

(4) $$\begin{array}{c} \rho \;C_{\frac{1}{1+\rho }}^{c}\left(\theta \right)=\min_{r\geq 0}\max_{P_{X}}E_{0}\left(\rho ,P_{X},r,\theta \right), \end{array}$$

where the maximization on the right side is unconstrained. In other words, the minimax of Gallager’s $E_{0}$ function (3) with cost constraints is shown to be equal to the maximal Augustin–Csiszár mutual information, thereby bridging the existing gap between the Phase 1 and Phase 3 representations alluded to earlier in this introduction.

As in [48], Section 10 defines the sphere-packing and random-coding error exponent functions in the natural canonical form of Phase 2 (e.g., (2)), and gives a very simple proof of the nexus between the Phase 2 and Phase 3 representations, namely,

(5) $$\begin{array}{c} E_{\mathrm{sp}}\left(R\right)=\underset{\rho \geq 0}{\sup}\;\left\{\rho \;C_{\frac{1}{1+\rho }}^{c}\left(\theta \right)-\rho \;R\right\}, \end{array}$$

with or without cost constraints. In this regard, we note that, although all the ingredients required were already present at the time the revised version of [24] was published three decades after the original, [48] does not cover the role of Rényi’s information measures in channel error exponents.

Examples illustrating the proposed method are given in Section 11 and Section 12 for the additive Gaussian noise channel under a quadratic cost function, and the additive exponential noise channel under a linear cost function, respectively. Simple parametric expressions are given for the error exponent functions, and the least favorable channels that account for the most likely error mechanism (Section 1.2) are identified in both cases.

## 2. Relative Information and Information Density

We begin with basic terminology and notation required for the subsequent development.

1. If (A,F,P) is a probability space, X∼P indicates P[X∈F]=P(F) for all F∈F.
2. If probability measures *P* and *Q* defined on the same measurable space (A,F) satisfy P(A)=0 for all A∈F such that Q(A)=0, we say that *P* is dominated by *Q*, denoted as P≪Q. If *P* and *Q* dominate each other, we write P≪≫Q. If there is an event such that P(A)=0 and Q(A)=1, we say that *P* and *Q* are mutually singular, and we write P⊥Q.
3. If P≪Q, then $\frac{dP}{dQ}$ is the Radon-Nikodym derivative of the dominated measure *P* with respect to the reference measure *Q*. Its logarithm is known as the relative information, namely, the random variable
  (6) $$\begin{array}{c} {ı}_{P∥Q}\left(a\right)=\log\frac{dP}{dQ}\left(a\right)\in \left[-\infty ,+\infty \right),\;a\in A. \end{array}$$
  As with the Radon-Nikodym derivative, any identity involving relative informations can be changed on a set of measure zero under the reference measure without incurring in any contradiction. If P≪Q≪R, then the chain rule of Radon-Nikodym derivatives yields
  (7) $$\begin{array}{c} {ı}_{P∥Q}\left(a\right)+{ı}_{Q∥R}\left(a\right)={ı}_{P∥R}\left(a\right),\;a\in A. \end{array}$$
  Throughout the paper, the base of exp and log is the same and chosen by the reader unless explicitly indicated otherwise. We frequently define a probability measure *P* from the specification of ${ı}_{P∥Q}$ and Q≫P since
  (8) $$\begin{array}{c} P\left(A\right)=\int_{A}\exp\left({ı}_{P∥Q}\left(a\right)\right)\;dQ\left(a\right),\;A\in F. \end{array}$$
  If X∼P and Y∼Q, it is often convenient to write ${ı}_{X∥Y}\left(x\right)$ instead of ${ı}_{P∥Q}\left(x\right)$. Note that
  (9) $$\begin{array}{c} E\left[\exp\left({ı}_{X∥Y}\left(Y\right)\right)\right]=1. \end{array}$$
  **Example** **1.** *If $X∼N\;\left(\mu_{X},\sigma_{X}^{2}\right)$ (Gaussian with mean $\mu_{X}$ and variance $\sigma_{X}^{2}$) and $Y∼N\;\left(\mu_{Y},\sigma_{Y}^{2}\right)$, then,*
  (10) $$\begin{array}{c} {ı}_{X∥Y}\left(a\right)=\frac{1}{2}\log\frac{\sigma_{Y}^{2}}{\sigma_{X}^{2}}+\frac{1}{2}\left(\frac{{\left(a-\mu_{Y}\right)}^{2}}{\sigma_{Y}^{2}}-\frac{{\left(a-\mu_{X}\right)}^{2}}{\sigma_{X}^{2}}\right)\log e. \end{array}$$
4. Let (A,F) and (B,G) be measurable spaces, known as the input and output spaces, respectively. Likewise, A and B are referred to as the input and output alphabets respectively. The simplified notation $P_{Y|X}:A\to B$ denotes a random transformation from (A,F) to (B,G), i.e., for any x∈A, $P_{Y|X=x}\left(\cdot \right)$ is a probability measure on (B,G), and for any B∈G, $P_{Y|X=\cdot }\left(B\right)$ is an F-measurable function.
5. We abbreviate by $P_{A}$ the set of probability measures on (A,F), and by $P_{A\times B}$ the set of probability measures on (A×B,F⊗G). If $P\in P_{A}$ and $P_{Y|X}:A\to B$ is a random transformation, the corresponding joint probability measure is denoted by $P\;P_{Y|X}\in P_{A\times B}$ (or, interchangeably, $P_{Y|X}P$). The notation $P\to P_{Y|X}\to Q$ simply indicates that the output marginal of the joint probability measure $P\;P_{Y|X}$ is denoted by $Q\in P_{B}$, namely,
  (11) $$\begin{array}{c} Q\left(B\right)=\int P_{Y|X}\left(B|x\right)\;dP_{X}\left(x\right)=E\left[P_{Y|X}\left(B|X\right)\right],\;B\in G. \end{array}$$
6. If $P_{X}\to P_{Y|X}\to P_{Y}$ and $P_{Y|X=a}\ll P_{Y}$, the information density ${ı}_{X;Y}:A\times B\to \left[-\infty ,\infty \right)$ is defined as
  (12) $$\begin{array}{c} {ı}_{X;Y}\left(a;b\right)={ı}_{P_{Y|X=a}∥P_{Y}}\left(b\right),\;\left(a,b\right)\in A\times B. \end{array}$$
  Following Rényi’s terminology [49], if $P_{X}P_{Y|X}\ll P_{X}\times P_{Y}$, the dependence between *X* and *Y* is said to be regular, and the information density can be defined on (x,y)∈A×B. Henceforth, we assume that $P_{Y|X}$ is such that the dependence between its input and output is regular regardless of the input probability measure. For example, if X=Y∈R, then $P_{Y|X=a}\left(A\right)=1\left\{a\in A\right\}$, and their dependence is not regular, since for any $P_{X}$ with non-discrete components *P_XY_* 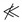 *P_X_* × *P_Y_*.
7. Let α>0, and $P_{X}\to P_{Y|X}\to P_{Y}$. The α-response to $P_{X}\in P_{A}$ is the output probability measure $P_{Y[\alpha ]}\ll P_{Y}$ with relative information given by
  (13) $$\begin{array}{c} {ı}_{Y[\alpha ]∥Y}\left(y\right)=\frac{1}{\alpha }\log E\left[\exp\left(\alpha \;{ı}_{X;Y}\left(X;y\right)-\kappa_{\alpha }\right)\right],\;X∼P_{X}, \end{array}$$
  where $\kappa_{\alpha }$ is a scalar that guarantees that $P_{Y[\alpha ]}$ is a probability measure. Invoking (9), we obtain
  (14) $$\begin{array}{cc} \kappa_{\alpha } & =\alpha \log E\left[E^{\frac{1}{\alpha }}\left[\exp\left(\alpha \;{ı}_{X;Y}\left(X;\bar{Y}\right)\right)|\bar{Y}\right]\right],\;\left(X,\bar{Y}\right)∼P_{X}\times P_{Y}. \end{array}$$
  For brevity, the dependence of $\kappa_{\alpha }$ on $P_{X}$ and $P_{Y|X}$ is omitted. Jensen’s inequality applied to ${\left(\cdot \right)}^{\alpha }$ results in $\kappa_{\alpha }\leq 0$ for α∈(0,1) and $\kappa_{\alpha }\geq 0$ for α>1. Although the α-response has a long record of services to information theory, this terminology and notation were introduced recently in [45]. Alternative terminology and notation were proposed in [42], which refers to the α-response as the order α Rényi mean. Note that $\kappa_{1}=0$ and the 1-response to $P_{X}$ is $P_{Y}$. If $p_{Y[\alpha ]}$ and $p_{Y|X}$ denote the densities of $P_{Y[\alpha ]}$ and $P_{Y|X}$ with respect to some common dominating measure, then (13) becomes
  (15) $$\begin{array}{c} p_{Y[\alpha ]}\left(y\right)=\exp\left(-\frac{\kappa_{\alpha }}{\alpha }\right)\;E^{\frac{1}{\alpha }}\left[p_{Y|X}^{\alpha }\left(y|X\right)\right],\;X∼P_{X}. \end{array}$$
  For α>1 (resp. α<1) we can think of the normalized version of $p_{Y|X}^{\alpha }$ as a random transformation with less (resp. more) "noise" than $p_{Y|X}$.
8. We will have opportunity to apply the following examples.

**Example** **2.** *If Y=X+N, where $X∼N\;\left(\mu_{X},\sigma_{X}^{2}\right)$ independent of $N∼N\;\left(\mu_{N},\sigma_{N}^{2}\right)$, then the α-response to $P_{X}$ is*

(16) $$\begin{array}{c} Y\left[\alpha \right]∼N\;\left(\mu_{X}+\mu_{N},\alpha \;\sigma_{X}^{2}+\sigma_{N}^{2}\right). \end{array}$$

**Example** **3.** *Suppose that Y=X+N, where N is exponential with mean ζ, independent of X, which is a mixed random variable with density*

(17) $$\begin{array}{c} f_{X}\left(t\right)=\frac{\zeta }{\alpha \;\mu }\delta \left(t\right)+\left(1-\frac{\zeta }{\alpha \;\mu }\right)\frac{1}{\mu }e^{-t/\mu }\;1\left\{t>0\right\}, \end{array}$$

*with αμ≥ζ. Then, Y[α], the α-response to $P_{X}$, is exponential with mean αμ.*

## 3. Relative Entropy and Rényi Divergence

Given a pair of probability measures $\left(P,Q\right)\in P_{A}^{2}$, relative entropy and Rényi divergence gauge the distinctness between *P* and *Q*.

9. Provided P≪Q, the relative entropy is the expectation of the relative information with respect to the dominated measure
  (18) $$\begin{array}{cc} D(P∥Q) & =E\left[{ı}_{P∥Q}\left(X\right)\right],\;X∼P \end{array}$$
  (19) $$\begin{array}{cc} \; & =E\left[\exp\left({ı}_{P∥Q}\left(Y\right)\right){ı}_{P∥Q}\left(Y\right)\right],\;Y∼Q \end{array}$$
  (20) $$\begin{array}{cc} \; & \geq 0, \end{array}$$
  with equality if and only if P=Q. If *P* 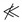 *Q*, then D(P∥Q)=∞. As in Item 3, if X∼P and Y∼Q, we may write D(X∥Y) instead of D(P∥Q), in the same spirit that the expectation and entropy of *P* are written as E[X] and H(X), respectively.
10. Arising in the sequel, a common optimization in information theory finds, among the probability measures satisfying an average cost constraint, that which is closest to a given reference measure *Q* in the sense of D(·∥Q). For that purpose, the following result proves sufficient. Incidentally, we often refer to unconstrained maximizations over probability distributions. It should be understood that those optimizations are still constrained to the sets $P_{A}$ or $P_{B}$. As customary in information theory, we will abbreviate $\max_{P_{X}\in P_{A}}$ by $\max_{X}$ or $\max_{P_{X}}$.
  **Theorem** **1.** *Let $P_{Z}\in P_{A}$ and suppose that g:A→[0,∞) is a Borel measurable mapping. Then,*
  (21) $$\begin{array}{c} \min_{X}\left\{D(X\;∥\;Z)+E[g(X)]\right\}=-\log E\left[\exp\left(-g\left(Z\right)\right)\right], \end{array}$$
achieved uniquely by $P_{X}^{*}\ll \gg P_{Z}$ defined by
  (22) $$\begin{array}{c} {ı}_{X^{*}∥Z}\left(a\right)=-g\left(a\right)-\log E[\exp(-g(Z))],\;a\in A. \end{array}$$
  **Proof.**  Note that since *g* is nonnegative, η=E[exp(−g(Z))]∈(0,1]. Furthermore,
  (23) $$\begin{array}{c} E\left[g\left(X^{*}\right)\right]=\frac{\int g\left(t\right)\exp\left(-g\left(t\right)\right)\;dP_{Z}\left(t\right)}{E[\exp(-g(Z))]}\in \left[0,\frac{1}{e\;\eta }\right]. \end{array}$$
  Therefore, the subset of $P_{A}$ for which the term in {·} in (21) is finite is nonempty: Fix any $P_{X}$ from that subset, (which therefore satisfies $P_{X}\ll P_{Z}\ll P_{X}^{*}$) and invoke the chain rule (7) to write
  (24) $$\begin{array}{cc} D(X\;∥\;Z)+E[g(X)]\; & =E\left[{ı}_{X∥X^{*}}\left(X\right)+{ı}_{X^{*}∥Z}\left(X\right)+g\left(X\right)\right] \end{array}$$
  (25) $$\begin{array}{cc} \; & =D\left(X\;∥\;X^{*}\right)-\log E[\exp(-g(Z))],\;X∼P_{X}, \end{array}$$
  which is uniquely minimized by letting $P_{X}=P_{X}^{*}$. Note that for typographical convenience we have denoted $X^{*}∼P_{X}^{*}$. □
11. Let *p* and *q* denote the Radon-Nikodym derivatives of probability measures *P* and *Q*, respectively, with respect to a common dominating σ-finite measure μ. The Rényi divergence of order α∈(0,1)∪(1,∞) between *P* and *Q* is defined as [25,50]
  (26) $$\begin{array}{cc} D_{\alpha }\left(P∥Q\right) & =\frac{1}{\alpha -1}\log\int_{A}p^{\alpha }q^{1-\alpha }d\mu \end{array}$$
  (27) $$\begin{array}{cc} \; & =\frac{1}{\alpha -1}\log E\left[\exp\left(\alpha \;{ı}_{P∥R}\left(Z\right)+\left(1-\alpha \right){ı}_{Q∥R}\left(Z\right)\right)\right],\;Z∼R \end{array}$$
  (28) $$\begin{array}{cc} \; & =\frac{1}{\alpha -1}\log E\left[\exp\left(\alpha \;{ı}_{P∥Q}\left(Y\right)\right)\right],\;Y∼Q \end{array}$$
  (29) $$\begin{array}{cc} \; & =\frac{1}{\alpha -1}\log E\left[\exp\left(\left(\alpha -1\right)\;{ı}_{P∥Q}\left(X\right)\right)\right],\;X∼P, \end{array}$$
  where (28) and (29) hold if P≪Q, and in (27), *R* is a probability measure that dominates both *P* and *Q*. Note that (28) and (29) state that $\left(t-1\right)D_{t}\left(X∥Y\right)$ and $t\;D_{1+t}\left(X∥Y\right)$ are the cumulant generating functions of the random variables ${ı}_{X∥Y}\left(Y\right)$ and ${ı}_{X∥Y}\left(X\right)$, respectively. The relative entropy is the limit of $D_{\alpha }\left(P∥Q\right)$ as α↑1, so it is customary to let $D_{1}\left(P∥Q\right)=D\left(P∥Q\right)$. For any α>0, $D_{\alpha }\left(P∥Q\right)\geq 0$ with equality if and only if P=Q. Furthermore, $D_{\alpha }\left(P∥Q\right)$ is non-decreasing in α, satisfies the skew-symmetric property
  (30) $$\begin{array}{cc} \left(1-\alpha \right)D_{\alpha }\left(P∥Q\right) & =\alpha \;D_{1-\alpha }\left(Q∥P\right),\;\alpha \in \left[0,1\right], \end{array}$$
  and
  (31) $$\begin{array}{c} \underset{\alpha \in (0,1)}{\inf}D_{\alpha }\left(P∥Q\right)=\infty ⟺P⊥Q⟹\underset{\alpha >1}{\inf}D_{\alpha }\left(P∥Q\right)=\infty . \end{array}$$
12. The expressions in the following pair of examples will come in handy in Section 11 and Section 12.
  **Example** **4.** *Suppose that $\sigma_{\alpha }^{2}=\alpha \;\sigma_{1}^{2}+\left(1-\alpha \right)\sigma_{0}^{2}>0$ and α∈(0,1)∪(1,∞). Then,*
  (32) $$\begin{array}{c} \;D_{\alpha }\left(N\;\left(\mu_{0},\sigma_{0}^{2}\right)\;∥\;N\;\left(\mu_{1},\sigma_{1}^{2}\right)\right)=\frac{1}{2}\log\frac{\sigma_{1}^{2}}{\sigma_{0}^{2}}+\frac{1}{2(\alpha -1)}\log\frac{\sigma_{1}^{2}}{\sigma_{\alpha }^{2}}+\frac{\alpha {\left(\mu_{1}-\mu_{0}\right)}^{2}}{2\;\sigma_{\alpha }^{2}}\log e, \end{array}$$
  (33) $$\begin{array}{cc} \;D\left(N\;\left(\mu_{0},\sigma_{0}^{2}\right)\;∥\;N\;\left(\mu_{1},\sigma_{1}^{2}\right)\right)\; & =\frac{1}{2}\log\frac{\sigma_{1}^{2}}{\sigma_{0}^{2}}+\frac{1}{2}\left(\frac{\sigma_{0}^{2}}{\sigma_{1}^{2}}-1\right)\log e+\frac{{\left(\mu_{1}-\mu_{0}\right)}^{2}}{2\;\sigma_{1}^{2}}\log e \end{array}$$
  (34) $$\begin{array}{cc} \; & =\lim_{\alpha \to 1}D_{\alpha }\left(N\;\left(\mu_{0},\sigma_{0}^{2}\right)\;∥\;N\;\left(\mu_{1},\sigma_{1}^{2}\right)\right). \end{array}$$
  **Example** **5.** *Suppose Z is exponentially distributed with unit mean, i.e., its probability density function is $e^{-t}1\left\{t\geq 0\right\}$. For $d_{0}\geq d_{1}$ and α such that $\left(1-\alpha \right)\;\mu_{0}+\alpha \;\mu_{1}>0$ we obtain*
  $$\begin{array}{cc} D_{\alpha }\left(\mu_{0}\;Z+d_{0}\;∥\;\mu_{1}\;Z+d_{1}\right)\; & =\frac{d_{0}-d_{1}}{\mu_{1}}\log e+\log\frac{\mu_{1}}{\mu_{0}}+\frac{1}{1-\alpha }\log\left(\alpha +\left(1-\alpha \right)\frac{\mu_{0}}{\mu_{1}}\right), \end{array}$$
  (35) $$\begin{array}{cc} D\left(\mu_{0}\;Z+d_{0}\;∥\;\mu_{1}\;Z+d_{1}\right) & =\left(\frac{\mu_{0}}{\mu_{1}}-1+\frac{d_{0}-d_{1}}{\mu_{1}}\right)\log e+\log\frac{\mu_{1}}{\mu_{0}} \end{array}$$
  (36) $$\begin{array}{cc} \; & =\lim_{\alpha \to 1}D_{\alpha }\left(\mu_{0}\;Z+d_{0}\;∥\;\mu_{1}\;Z+d_{1}\right). \end{array}$$
13. Intimately connected with the notion of Rényi divergence is the tilted probability measure $P_{\alpha }$ defined, if $D_{\alpha }\left(P_{1}∥P_{0}\right)<\infty$, by
  (37) $$\begin{array}{c} {ı}_{P_{\alpha }∥Q}\left(a\right)=\alpha \;{ı}_{P_{1}∥Q}\left(a\right)+\left(1-\alpha \right)\;{ı}_{P_{0}∥Q}\left(a\right)+\left(1-\alpha \right)D_{\alpha }\left(P_{1}\;∥\;P_{0}\right), \end{array}$$
  where *Q* is any probability measure that dominates both $P_{0}$ and $P_{1}$. Although (37) is defined in general, our main emphasis is on the range α∈(0,1), in which, as long as *P*_0_ 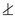 *P*_1_, the tilted probability measure is defined and satisfies $P_{\alpha }\ll P_{0}$ and $P_{\alpha }\ll P_{1}$, with corresponding relative informations
  (38) $$\begin{array}{cc} {ı}_{P_{\alpha }∥P_{0}}\left(a\right) & ={ı}_{P_{\alpha }∥Q}\left(a\right)-{ı}_{P_{0}∥Q}\left(a\right) \end{array}$$
  (39) $$\begin{array}{c} =\left(1-\alpha \right)D_{\alpha }\left(P_{1}\;∥\;P_{0}\right)+\alpha \;\left({ı}_{P_{1}∥Q}\left(a\right)-{ı}_{P_{0}∥Q}\left(a\right)\right), \end{array}$$
  (40) $$\begin{array}{cc} {ı}_{P_{\alpha }∥P_{1}}\left(a\right) & ={ı}_{P_{\alpha }∥Q}\left(a\right)-{ı}_{P_{1}∥Q}\left(a\right) \end{array}$$
  (41) $$\begin{array}{c} =\left(1-\alpha \right)D_{\alpha }\left(P_{1}\;∥\;P_{0}\right)-\left(1-\alpha \right)\left({ı}_{P_{1}∥Q}\left(a\right)-{ı}_{P_{0}∥Q}\left(a\right)\right), \end{array}$$
  where we have used the chain rule for $P_{\alpha }\ll P_{0}\ll Q$ and $P_{\alpha }\ll P_{1}\ll Q$. Taking a linear combination of (38)–(41) we conclude that, for all a∈A,
  (42) $$\begin{array}{cc} \left(1-\alpha \right)D_{\alpha }\left(P_{1}∥P_{0}\right) & =\left(1-\alpha \right)\;{ı}_{P_{\alpha }∥P_{0}}\left(a\right)+\alpha \;{ı}_{P_{\alpha }∥P_{1}}\left(a\right). \end{array}$$
  Henceforth, we focus particular attention on the case α∈(0,1) since that is the region of interest in the application of Rényi information measures to the evaluation of error exponents in channel coding for codes whose rate is below capacity. In addition, often proofs simplify considerably for α∈(0,1).
14. Much of the interplay between relative entropy and Rényi divergence hinges on the following identity, which appears, without proof, in (3) of [51].
  **Theorem** **2.** *Let α∈(0,1) and assume that *P*_0_
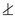
*P*_1_ are defined on the same measurable space. Then, for any $P\ll P_{1}$ and $P\ll P_{0}$,*
  (43) $$\begin{array}{c} \alpha \;D\left(P\;∥P_{1}\right)+\left(1-\alpha \right)\;D\left(P\;∥P_{0}\right)=D\left(P\;∥P_{\alpha }\right)+\left(1-\alpha \right)D_{\alpha }\left(P_{1}\;∥P_{0}\right), \end{array}$$
*where $P_{\alpha }$ is the tilted probability measure in (37) and (43) holds regardless of whether the relative entropies are finite. In particular,*
  (44) $$\begin{array}{c} D\left(P\;∥\;P_{\alpha }\right)<\infty ⟺\max\left\{D\left(P\;∥\;P_{0}\right),D\left(P\;∥\;P_{1}\right)\right\}<\infty . \end{array}$$
  **Proof.**  We distinguish three overlapping cases:
  (1) $D(P\;∥\;P_{\alpha })<\infty$: Taking expectation of (42) with respect to a←X∼P, yields (43) because
    (45) $$\begin{array}{cc} E\left[{ı}_{P_{\alpha }∥P_{0}}\left(X\right)\right] & =D\left(P∥P_{0}\right)-D\left(P∥P_{\alpha }\right), \end{array}$$
    (46) $$\begin{array}{cc} E\left[{ı}_{P_{\alpha }∥P_{1}}\left(X\right)\right] & =D\left(P∥P_{1}\right)-D\left(P∥P_{\alpha }\right), \end{array}$$
    where, thanks to the assumption that $D(P\;∥\;P_{\alpha })<\infty$, we have invoked Corollary A1 in the Appendix A twice with $\left(P,Q,R\right)\leftarrow \left(P,P_{\alpha },P_{0}\right)$ and $\left(P,Q,R\right)\leftarrow \left(P,P_{\alpha },P_{1}\right)$, respectively;
  (2) $\max\{D\left(P\;∥\;P_{0}\right),D\left(P\;∥\;P_{1}\right)\}<\infty$: The proof is identical since we are entitled to invoke Corollary A1 to show (45) (resp., (46)) because $D(P\;∥\;P_{0})<\infty$ (resp., $D(P\;∥\;P_{1})<\infty$).
  (3) $D(P\;∥\;P_{\alpha })=\infty$ and $\max\{D\left(P\;∥\;P_{0}\right),D\left(P\;∥\;P_{1}\right)\}=\infty$: both sides of (43) are equal to *∞*.
  Finally, to show that (44) follows from (43), simply recall from (31) that $D_{\alpha }\left(P_{1}\;∥P_{0}\right)<\infty$. □
15. Relative entropy and Rényi divergence are related by the following fundamental variational representation.
  **Theorem** **3.** *Fix α∈(0,1) and $\left(P_{1},P_{0}\right)\in P_{A}^{2}$. Then, the Rényi divergence between $P_{1}$ and $P_{0}$ satisfies*
  (47) $$\begin{array}{c} \left(1-\alpha \right)\;D_{\alpha }\left(P_{1}∥P_{0}\right)=\min_{P}\left\{\alpha \;D\left(P∥P_{1}\right)+\left(1-\alpha \right)\;D\left(P∥P_{0}\right)\right\}, \end{array}$$
*where the minimum is over $P_{A}$. If *P*_0_
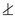
*P*_1_, then the right side of (47) is attained by the tilted measure $P_{\alpha }$, and the minimization can be restricted to the subset of probability measures which are dominated by both $P_{1}$ and $P_{0}$.*
  **Proof.**  If $P_{0}⊥P_{1}$, then both sides of (47) are +∞ since there is no probability measure that is dominated by both $P_{0}$ and $P_{1}$. If *P*_0_ 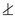 *P*_1_, then minimizing both sides of (43) with respect to *P* yields (47) and the fact that the tilted probability measure attains the minimum therein. □
  The variational representation in (47) was observed in [39] in the finite-alphabet case, and, contemporaneously, in full generality in [50]. Unlike Theorem 3, both of those references also deal with α>1. The function $d\left(\alpha \right)=\left(1-\alpha \right)\;D_{\alpha }\left(P_{1}∥P_{0}\right)$, with $d\left(1\right)=\lim_{\alpha ↑1}d\left(\alpha \right)$, is concave in α because the right side of (47) is a minimum of affine functions of α.
16. Given random transformations $P_{Y|X}:A\to B$, $Q_{Y|X}:A\to B$, and a probability measure $P_{X}\in P_{A}$ on the input space, the conditional relative entropy is
  (48) $$\begin{array}{cc} D(P_{Y|X}\;∥\;Q_{Y|X}\;|\;P_{X}) & =D(P_{Y|X}P_{X}\;∥\;Q_{Y|X}P_{X}) \end{array}$$
  (49) $$\begin{array}{cc} \; & =E\left[D\left(P_{Y|X}\left(\cdot |X\right)\;∥\;Q_{Y|X}\left(\cdot |X\right)\right)\right],\;X∼P_{X}. \end{array}$$
  Analogously, the conditional Rényi divergence is defined as
  (50) $$\begin{array}{c} D_{\alpha }(P_{Y|X}\;∥\;Q_{Y|X}\;\left|\;P_{X}\right)=D_{\alpha }\left(P_{Y|X}P_{X}\;∥\;Q_{Y|X}P_{X}\right). \end{array}$$
  A word of caution: the notation in (50) conforms to that in [38,45] but it is not universally adopted, e.g., [43] uses the left side of (50) to denote the Rényi generalization of the right side of (49). We can express the conditional Rényi divergence as
  $$\begin{array}{cc} \; & \;D_{\alpha }(P_{Y|X}\;∥\;Q_{Y|X}\left|P_{X}\right) \end{array}$$
  (51) $$\begin{array}{cc} \; & =\frac{1}{\alpha -1}\log E\left[\exp\left(\left(\alpha -1\right)D_{\alpha }\left(P_{Y|X}\left(\cdot |X\right)\;∥\;Q_{Y|X}\left(\cdot |X\right)\right)\right)\right],\;X∼P_{X}, \end{array}$$
  (52) $$\begin{array}{cc} \; & =\frac{1}{\alpha -1}\log E\left[{\left(\frac{dP_{Y|X}}{dQ_{Y|X}}\left(Y|X\right)\right)}^{\alpha -1}\right],\;\left(X,Y\right)∼P_{X}P_{Y|X}, \end{array}$$
  where (52) holds if $P_{X}P_{Y|X}\ll P_{X}Q_{Y|X}$. Jensen’s inequality applied to (51) results in
  (53) $$\begin{array}{cc} D_{\alpha }(P_{Y|X}\;∥\;Q_{Y|X}\left|P_{X}\right) & \leq E\left[D_{\alpha }\left(P_{Y|X}\left(\cdot |X\right)\;∥\;Q_{Y|X}\left(\cdot |X\right)\right)\right],\;\alpha \in \left(0,1\right); \end{array}$$
  (54) $$\begin{array}{cc} D_{\alpha }(P_{Y|X}\;∥\;Q_{Y|X}\left|P_{X}\right) & \geq E\left[D_{\alpha }\left(P_{Y|X}\left(\cdot |X\right)\;∥\;Q_{Y|X}\left(\cdot |X\right)\right)\right],\;\alpha >1. \end{array}$$
  Nevertheless, an immediate and crucial observation we can draw from (51) is that the unconstrained maximizations of the sides of (53) and of (54) over $P_{X}$ do coincide: for all α>0,
  (55) $$\begin{array}{cc} \underset{X}{\sup}D_{\alpha }(P_{Y|X}\;∥\;Q_{Y|X}\left|P_{X}\right) & =\underset{X}{\sup}E\left[D_{\alpha }\left(P_{Y|X}\left(\cdot |X\right)\;∥\;Q_{Y|X}\left(\cdot |X\right)\right)\right] \end{array}$$
  (56) $$\begin{array}{cc} \; & =\underset{a\in A}{\sup}D_{\alpha }\left(P_{Y|X=a}\;∥\;Q_{Y|X=a}\right). \end{array}$$
17. Conditional Rényi divergence satisfies the following additive decomposition, originally pointed out, without proof, by Sibson [31] in the setting of finite A.
  **Theorem** **4.** *Given $P_{X}\in P_{A}$, $Q_{Y}\in P_{B}$, $P_{Y|X}:A\to B$, and α∈(0,1)∪(1,∞), we have*
  (57) $$\begin{array}{c} D_{\alpha }(P_{Y|X}\;∥\;Q_{Y}|P_{X})=D_{\alpha }(P_{Y|X}\;∥\;P_{Y[\alpha ]}\left|P_{X}\right)+D_{\alpha }\left(P_{Y[\alpha ]}∥Q_{Y}\right). \end{array}$$
*Furthermore, with $\kappa_{\alpha }$ as in (14),*
  (58) $$\begin{array}{c} D_{\alpha }\left(P_{Y|X}\;∥\;P_{Y[\alpha ]}|P_{X}\right)=\frac{\kappa_{\alpha }}{\alpha -1}. \end{array}$$
  **Proof.** Select an arbitrary probability measure $R_{Y}\in P_{B}$ that dominates both $Q_{Y}$ and $P_{Y}$, and, therefore, $P_{Y[\alpha ]}$ too. Letting $\left(X,Z\right)∼P_{X}\times R_{Y}$, we have
  (59) $$\begin{array}{cc} \; & D_{\alpha }(P_{Y|X}\;∥\;Q_{Y}\left|P_{X}\right)=\frac{1}{\alpha -1}\log E\left[{\left(\frac{dP_{XY}}{dP_{X}\times R_{Y}}\left(X,Z\right)\right)}^{\alpha }{\left(\frac{dQ_{Y}}{dR_{Y}}\left(Z\right)\right)}^{1-\alpha }\right] \end{array}$$
  (60) $$\begin{array}{cc} \; & =\frac{1}{\alpha -1}\log E\left[E\left[\exp\left(\alpha \;{ı}_{X;Y}\left(X;Z\right)\right)|Z\right]{\left(\frac{dP_{Y}}{dR_{Y}}\left(Z\right)\right)}^{\alpha }{\left(\frac{dQ_{Y}}{dR_{Y}}\left(Z\right)\right)}^{1-\alpha }\right] \end{array}$$
  (61) $$\begin{array}{cc} \; & =\frac{\kappa_{\alpha }}{\alpha -1}+\frac{1}{\alpha -1}\log E\left[{\left(\frac{dP_{Y[\alpha ]}}{dP_{Y}}\left(Z\right)\right)}^{\alpha }{\left(\frac{dP_{Y}}{dR_{Y}}\left(Z\right)\right)}^{\alpha }{\left(\frac{dQ_{Y}}{dR_{Y}}\left(Z\right)\right)}^{1-\alpha }\right] \end{array}$$
  (62) $$\begin{array}{cc} \; & =\frac{\kappa_{\alpha }}{\alpha -1}+\frac{1}{\alpha -1}\log E\left[{\left(\frac{dP_{Y[\alpha ]}}{dR_{Y}}\left(Z\right)\right)}^{\alpha }{\left(\frac{dQ_{Y}}{dR_{Y}}\left(Z\right)\right)}^{1-\alpha }\right] \end{array}$$
  (63) $$\begin{array}{cc} \; & =\frac{\kappa_{\alpha }}{\alpha -1}+D_{\alpha }\left(P_{Y[\alpha ]}∥Q_{Y}\right), \end{array}$$
  where (61) follows from (13), and (62) follows from the chain rule of Radon-Nikodym derivatives applied to $P_{Y[\alpha ]}\ll P_{Y}\ll R_{Y}$. Then, (58) follows by specializing $Q_{Y}=P_{Y[\alpha ]}$, and the proof of (57) is complete, upon plugging (58) into the right side of (63). □
  A proof of (57) in the discrete case can be found in Appendix A of [37].
18. For all α>0, given two inputs $\left(P_{X},Q_{X}\right)\in P_{A}^{2}$ and one random transformation $P_{Y|X}:A\to B$, Rényi divergence (and, in particular, relative entropy) satisfies the data processing inequality,
  (64) $$\begin{array}{c} D_{\alpha }\left(P_{X}\;∥\;Q_{X}\right)\geq D_{\alpha }\left(P_{Y}\;∥\;Q_{Y}\right), \end{array}$$
  where $P_{X}\to P_{Y|X}\to P_{Y}$, and $Q_{X}\to P_{Y|X}\to Q_{Y}$. The data processing inequality for Rényi divergence was observed by Csiszár [52] in the more general context of *f*-divergences. More recently it was stated in [39,50]. Furthermore, given one input $P_{X}\in P_{A}$ and two transformations $P_{Y|X}:A\to B$ and $Q_{Y|X}:A\to B$, conditioning cannot decrease Rényi divergence,
  (65) $$\begin{array}{c} D_{\alpha }(P_{Y|X}\;∥\;Q_{Y|X}\left|P_{X}\right)\geq D_{\alpha }\left(P_{Y}\;∥\;Q_{Y}\right). \end{array}$$
  Since $D_{\alpha }(P_{Y|X}\;∥\;Q_{Y|X}\left|P_{X}\right)=D_{\alpha }\left(P_{X}P_{Y|X}\;∥\;P_{X}Q_{Y|X}\right)$, (65) follows by applying (64) to a deterministic transformation which takes an input pair and outputs the second component. Inequalities (53) and (65) imply the convexity of $D_{\alpha }\left(P∥Q\right)$ in (P,Q) for α∈(0,1].

## 4. Dependence Measures

In this paper we are interested in three information measures that quantify the dependence between random variables *X* and *Y*, such that $P_{X}\to P_{Y|X}\to P_{Y}$, namely, mutual information, and two of its generalizations, α- mutual information and Augustin–Csiszár mutual information of order α.

19. The mutual information is
  (66) $$\begin{array}{cc} I\left(X;Y\right)=I\left(P_{X},P_{Y|X}\right) & =D(P_{Y|X}\;∥\;P_{Y}\;|\;P_{X}) \end{array}$$
  (67) $$\begin{array}{cc} \; & =\min_{Q_{Y}}D(P_{Y|X}\;∥\;Q_{Y}\;\left|\;P_{X}\right) \end{array}$$
  (68) $$\begin{array}{cc} \; & =\min_{Q_{Y}}D\left(P_{XY}\;∥\;P_{X}\times Q_{Y}\right). \end{array}$$
20. Given α∈(0,1)∪(1,∞), the α-mutual information is defined as (see [30,31,32,40,42,45])
  (69) $$\begin{array}{cc} \; & \;I_{\alpha }\left(X;Y\right)=I_{\alpha }\left(P_{X},P_{Y|X}\right) \end{array}$$
  (70) $$\begin{array}{cc} \; & =\min_{Q_{Y}}D_{\alpha }(P_{Y|X}\;∥\;Q_{Y}\;\left|\;P_{X}\right) \end{array}$$
  (71) $$\begin{array}{cc} \; & =\min_{Q_{Y}}D_{\alpha }\left(P_{XY}\;∥\;P_{X}\times Q_{Y}\right) \end{array}$$
  (72) $$\begin{array}{cc} \; & =D_{\alpha }\left(P_{Y|X}\;∥\;P_{Y[\alpha ]}\;|\;P_{X}\right) \end{array}$$
  (73) $$\begin{array}{cc} \; & =\frac{1}{\alpha -1}\log E\left[\exp\left(\left(\alpha -1\right)D_{\alpha }\left(P_{Y|X}\left(\cdot |X\right)\;∥\;P_{Y[\alpha ]}\right)\right)\right],\;X∼P_{X} \end{array}$$
  (74) $$\begin{array}{cc} \; & =D_{\alpha }\left(P_{Y|X}\;∥\;P_{Y}|P_{X}\right)-D_{\alpha }\left(P_{Y[\alpha ]}\;∥\;P_{Y}\right) \end{array}$$
  (75) $$\begin{array}{cc} \; & =\frac{\kappa_{\alpha }}{\alpha -1} \end{array}$$
  (76) $$\begin{array}{cc} \; & =\frac{\alpha }{\alpha -1}\log E\left[E^{\frac{1}{\alpha }}\left[\exp\left(\alpha \;{ı}_{X;Y}\left(X;\bar{Y}\right)\right)\;|\;\bar{Y}\right]\right],\;\left(X,\bar{Y}\right)∼P_{X}\times P_{Y}, \end{array}$$
  where (72) and (74) follow from (57); (73) is a special case of (51); (75) follows from Theorem 4; and, (76) is (14). In view of (67) and (69), we let $I_{1}\left(X;Y\right)=I\left(X;Y\right)$. The notation we use for α-mutual information conforms to that used in [40,42,45,53]. Other notations include $K_{\alpha }$ in [32,38,39] and $I_{\alpha }^{g}$ in [43]. $I_{0}\left(X;Y\right)$ and $I_{\infty }\left(X;Y\right)$ are defined by taking the corresponding limits.
21. Theorem 4 and (72) result in the additive decomposition
  (77) $$\begin{array}{c} I_{\alpha }\left(X;Y\right)=D_{\alpha }(P_{Y|X}\;∥\;Q_{Y}\left|P_{X}\right)-D_{\alpha }\left(P_{Y[\alpha ]}\;∥\;Q_{Y}\right), \end{array}$$
  for any $Q_{Y}$ with $D_{\alpha }\left(P_{Y[\alpha ]}\;∥\;Q_{Y}\right)<\infty$, thereby generalizing the well-known decomposition for mutual information,
  (78) $$\begin{array}{c} I\left(X;Y\right)=D(P_{Y|X}\;∥\;Q_{Y}\left|P_{X}\right)-D\left(P_{Y}\;∥\;Q_{Y}\right), \end{array}$$
  which, in contrast to (77), is a simple consequence of the chain rule whenever the dependence between *X* and *Y* is regular, and of Lemma A1 in general.
22.
  **Example** **6.** *Additive independent Gaussian noise. If Y=X+N, where $X∼N\;\left(0,\sigma_{X}^{2}\right)$ independent of $N∼N\;\left(0,\sigma_{N}^{2}\right)$, then, for α>0,*
  (79) $$\begin{array}{cc} Y[\alpha ] & \;∼N\;\left(0,\alpha \;\sigma_{X}^{2}+\sigma_{N}^{2}\right), \end{array}$$
  (80) $$\begin{array}{cc} I_{\alpha }\left(X;X+N\right) & =I_{\alpha }\left(X+N;X\right)=\frac{1}{2}\log\left(1+\alpha \;\frac{\sigma_{X}^{2}}{\sigma_{N}^{2}}\right). \end{array}$$
23. If α∈(0,1), (47) and (69) result in
  (81) $$\begin{array}{c} \left(1-\alpha \right)I_{\alpha }\left(P_{X},P_{Y|X}\right)\\ =\min_{Q_{X}Q_{Y|X}}\left\{D\left(Q_{X}\;∥\;P_{X}\right)+\alpha \;D(Q_{Y|X}\;∥\;P_{Y|X}\;\left|\;Q_{X}\right)+\left(1-\alpha \right)\;I\left(Q_{X},Q_{Y|X}\right)\right\}. \end{array}$$
  For α>1 a proof of (81) is given in [39] for finite alphabets.
24. Unlike $I(P_{X},P_{Y|X})$, we can express $I_{\alpha }\left(P_{X},P_{Y|X}\right)$ directly in terms of its arguments without involving the corresponding output distribution or the α-response to $P_{X}$. This is most evident in the case of discrete alphabets, in which (76) becomes
  (82) $$\begin{array}{cc} I_{\alpha }\left(X;Y\right) & =\frac{\alpha }{\alpha -1}\log\sum_{y\in B}{\left(\sum_{x\in A}P_{X}\left(x\right)P_{Y|X=x}^{\alpha }\left(y\right)\right)}^{\frac{1}{\alpha }}\;, \end{array}$$
  (83) $$\begin{array}{cc} I_{0}\left(X;Y\right) & =-\log\max_{y\in B}\sum_{x\in A}P_{X}\left(x\right)1\left\{P_{Y|X}\left(y|x\right)>0\right\}, \end{array}$$
  (84) $$\begin{array}{cc} I_{\infty }\left(X;Y\right) & =\log\left(\sum_{b\in Y}\;\;\underset{a:P_{X}\left(a\right)>0}{\sup}P_{Y|X}\left(b|a\right)\right). \end{array}$$
  For example, if *X* is discrete and $H_{\alpha }\left(X\right)$ denotes the Rényi entropy of order α, then for all α>0,
  (85) $$\begin{array}{c} H_{\alpha }\left(X\right)=I_{\frac{1}{\alpha }}\left(X;X\right). \end{array}$$
  If *X* and *Y* are equiprobable with P[X≠Y]=δ, then, in bits, $I_{\alpha }\left(X;Y\right)=1-h_{\alpha }\left(\delta \right)$, where $h_{\alpha }\left(\delta \right)$ denotes the binary Rényi entropy.
25. In the main region of interest, namely, α∈(0,1), frequently we use a different parametrization in terms of ρ>0, with $\alpha =\frac{1}{1+\rho }$.
  **Theorem** **5.** *For any ρ>0, we have the upper bound*
  (86) $$\begin{array}{c} \rho \;I_{\frac{1}{1+\rho }}\left(X;Y\right)\leq \min_{Q_{Y|X}:A\to B}\left\{D(Q_{Y|X}∥P_{Y|X}\;\left|\;P_{X}\right)+\rho \;I\left(P_{X},Q_{Y|X}\right)\right\}. \end{array}$$
  **Proof.**  Fix $Q_{Y|X}:A\to B$, and let $P_{X}\to Q_{Y|X}\to Q_{Y}$. Then,
  (87) $$\begin{array}{cc} I_{\frac{1}{1+\rho }}\left(X;Y\right)\; & \leq D_{\frac{1}{1+\rho }}\left(P_{XY}∥P_{X}\times Q_{Y}\right) \end{array}$$
  (88) $$\begin{array}{cc} \; & =\frac{1+\rho }{\rho }\min_{R_{XY}}\left\{\frac{1}{1+\rho }D\left(R_{XY}∥P_{XY}\right)+\frac{\rho }{1+\rho }D\left(R_{XY}∥P_{X}\times Q_{Y}\right)\right\} \end{array}$$
  (89) $$\begin{array}{cc} \; & \leq \frac{1}{\rho }\;D\left(Q_{Y|X}P_{X}∥P_{XY}\right)+D\left(Q_{Y|X}P_{X}∥P_{X}\times Q_{Y}\right) \end{array}$$
  (90) $$\begin{array}{cc} \; & =\frac{1}{\rho }\;D(Q_{Y|X}∥P_{Y|X}\left|P_{X}\right)+I\left(P_{X},Q_{Y|X}\right), \end{array}$$
  where (87), (88) and (90) follow from (69), (47) and (66) respectively. □
  Just like (53), we will show in Section 7 that (86) becomes an equality upon the unconstrained maximization of both sides.
26. Before introducing the last dependence measure in this section, recall from Definition 7 and (58) that $P_{Y[\alpha ]}\ll P_{Y}$, the α-response (of $P_{Y|X}$) to $P_{X}$ defined by
  (91) $$\begin{array}{c} {ı}_{Y[\alpha ]∥Y}\left(y\right)=\frac{1}{\alpha }\log E\left[\exp\left(\alpha \;{ı}_{X;Y}\left(X;y\right)+\left(1-\alpha \right)D_{\alpha }\left(P_{Y|X}\;∥\;P_{Y[\alpha ]}|P_{X}\right)\right)\right], \end{array}$$
  attains $\min_{Q_{Y}}D_{\alpha }(P_{Y|X}∥Q_{Y}\left|P_{X}\right)$, where the expectation is with respect to $X∼P_{X}$. We proceed to define $P_{Y\langle \alpha \rangle }\ll P_{Y}$, the 〈α〉-response (of $P_{Y|X}$) to $P_{X}$ by means of
  (92) $$\begin{array}{c} {ı}_{Y\langle \alpha \rangle ∥Y}\left(y\right)=\frac{1}{\alpha }\log E\left[\exp(\alpha \;{ı}_{X;Y}\left(X;y\right)+\left(1-\alpha \right)D_{\alpha }\left(P_{Y|X}\left(\cdot |X\right)\;∥\;P_{Y\langle \alpha \rangle }\right)\right], \end{array}$$
  with $X∼P_{X}$. Note that $P_{Y\langle 1\rangle }=P_{Y[1]}=P_{Y}$.
27. In the case of discrete alphabets, (92) becomes the implicit equation
  (93) $$\begin{array}{c} P_{Y\langle \alpha \rangle }^{\alpha }\left(y\right)=\sum_{a\in A}P_{X}\left(a\right)\frac{P_{Y|X}^{\alpha }\left(y|a\right)}{\sum_{b\in B}P_{Y|X}^{\alpha }\left(b|a\right)\;P_{Y\langle \alpha \rangle }^{1-\alpha }\left(b\right)},\;y\in B, \end{array}$$
  which coincides with (9.24) in Fano’s 1961 textbook [7], with s←1−α, and is also given by Haroutunian in (19) of [22]. For example, if A=B is discrete and Y=X, then $P_{Y\langle \alpha \rangle }=P_{X}$, while $P_{Y[\alpha ]}^{\alpha }\left(y\right)=c\;P_{X}\left(y\right)$, y∈A.
28. The 〈α〉-response satisfies the following identity, which can be regarded as the counterpart of (57) satisfied by the α-response.
  **Theorem** **6.** *Fix $P_{X}\in P_{A}$, $P_{Y|X}:A\to B$ and $Q_{Y}\in P_{B}$. Then,*
  (94) $$\begin{array}{c} D_{\alpha }\left(P_{Y\langle \alpha \rangle }\;∥\;Q_{Y}\right)\\ =\frac{1}{\alpha -1}\log E\left[\exp\left(\left(1-\alpha \right)\left(D_{\alpha }\left(P_{Y|X}\left(\cdot |X\right)∥P_{Y\langle \alpha \rangle }\right)-D_{\alpha }\left(P_{Y|X}\left(\cdot |X\right)∥Q_{Y}\right)\right)\right)\right]. \end{array}$$
  **Proof.**  For brevity we assume $Q_{Y}\ll P_{Y}$. Otherwise, the proof is similar adopting a reference measure that dominates both $Q_{Y}$ and $P_{Y}$. The definition of unconditional Rényi divergence in Item 11 implies that we can write (α−1) times the exponential of the left side of (94) as
  (95) $$\begin{array}{cc} \; & \exp\left(\left(\alpha -1\right)D_{\alpha }\left(P_{Y\langle \alpha \rangle }∥Q_{Y}\right)\right)=E\left[{\left(\frac{dP_{Y\langle \alpha \rangle }}{dP_{Y}}\left(Y\right)\right)}^{\alpha }{\left(\frac{dQ_{Y}}{dP_{Y}}\left(Y\right)\right)}^{1-\alpha }\right] \end{array}$$
  (96) $$\begin{array}{cc} \; & =E\left[\exp\left(\alpha \;{ı}_{X;Y}\left(X;Y\right)+\left(1-\alpha \right)D_{\alpha }\left(P_{Y|X}\left(\cdot |X\right)\;∥\;P_{Y\langle \alpha \rangle }\right)\right){\left(\frac{dQ_{Y}}{dP_{Y}}\left(Y\right)\right)}^{1-\alpha }\right]\\ \; & =E\left[E\left[\exp\left(\alpha \;{ı}_{X;Y}\left(X;Y\right)+\left(1-\alpha \right)\left({ı}_{Q_{Y}∥P_{Y}}\left(Y\right)+D_{\alpha }\left(P_{Y|X}\left(\cdot |X\right)\;∥\;P_{Y\langle \alpha \rangle }\right)\right)\right)|X\right]\right] \end{array}$$
  (97) $$\begin{array}{cc} \; & =E\left[\exp\left(\left(1-\alpha \right)\left(D_{\alpha }\left(P_{Y|X}\left(\cdot |X\right)\;∥\;P_{Y\langle \alpha \rangle }\right)-D_{\alpha }\left(P_{Y|X}\left(\cdot |X\right)\;∥\;Q_{Y}\right)\right)\right)\right], \end{array}$$
  where $\left(X,Y\right)∼P_{X}\times P_{Y}$, (96) follows from (92), and (97) follows from the definition of unconditional Rényi divergence in (27). □
  **Theorem** **7.** *If α∈(0,1], then*
  (98) $$\begin{array}{cc} D_{\alpha }\left(P_{Y\langle \alpha \rangle }\;∥\;Q_{Y}\right) & \leq E\left[D_{\alpha }\left(P_{Y|X}\left(\cdot |X\right)\;∥\;Q_{Y}\right)\right]-E\left[D_{\alpha }\left(P_{Y|X}\left(\cdot |X\right)\;∥\;P_{Y\langle \alpha \rangle }\right)\right] \end{array}$$
  (99) $$\begin{array}{cc} \; & \leq D(P_{Y\langle \alpha \rangle }\;∥\;Q_{Y}). \end{array}$$
  *If α≥1, inequalities (98) and (99) are reversed.*
  **Proof.**  Assume α∈(0,1]. Jensen’s inequality applied to the right side of (94) results in (98). To show (99), again we assume for brevity $Q_{Y}\ll P_{Y}$, and define the positive functions V:A×B→(0,∞) and W:A×B→(0,∞),
  (100) $$\begin{array}{cc} V(x,y) & =\exp\left(\alpha {ı}_{X;Y}\left(x;y\right)+\left(1-\alpha \right){ı}_{Y\langle \alpha \rangle ∥Y}\left(y\right)\right), \end{array}$$
  (101) $$\begin{array}{cc} W(x,y) & =\exp\left(\alpha {ı}_{X;Y}\left(x;y\right)+\left(1-\alpha \right){ı}_{Q_{Y}∥P_{Y}}\left(y\right)\right). \end{array}$$
  Note that, with $\left(X,Y\right)∼P_{X}\times P_{Y}$, and (x,y)∈A×B,
  (102) $$\begin{array}{cc} E[V(x,Y)] & =\exp\left(\left(\alpha -1\right)D_{\alpha }\left(P_{Y|X=x}∥P_{Y\langle \alpha \rangle }\right)\right), \end{array}$$
  (103) $$\begin{array}{cc} E[W(x,Y)] & =\exp\left(\left(\alpha -1\right)D_{\alpha }\left(P_{Y|X=x}∥Q_{Y}\right)\right),\\ E\left[\frac{V(X,y)}{E[V(X,Y)|X]}\right] & =\exp\left(\left(1-\alpha \right){ı}_{Y\langle \alpha \rangle ∥Y}\left(y\right)\right)\cdot \end{array}$$
  (104) =·EexpαıX;Y(X;y)+(1−α)Dα(PY|X(·|X)∥PY〈α〉)
  (105) $$\begin{array}{cc} \; & =\frac{dP_{Y\langle \alpha \rangle }}{dP_{Y}}\left(y\right). \end{array}$$
  where (104) uses (100) and (102) and (105) follows from (92). Then,
  $$\begin{array}{cc} \; & \;D_{\alpha }\left(P_{Y|X=x}\;∥\;Q_{Y}\right)-D_{\alpha }\left(P_{Y|X=x}\;∥\;P_{Y\langle \alpha \rangle }\right) \end{array}$$
  (106) $$\begin{array}{cc} \; & =\frac{1}{1-\alpha }\log\frac{E[V(x,Y)]}{E[W(x,Y)]} \end{array}$$
  (107) $$\begin{array}{cc} \; & \leq \frac{1}{1-\alpha }E\left[\frac{V(x,Y)}{E[V(x,Y)]}\log\frac{V(x,Y)}{W(x,Y)}\right] \end{array}$$
  (108) $$\begin{array}{cc} \; & =E\left[\frac{V(x,Y)}{E[V(x,Y)]}\left({ı}_{Y\langle \alpha \rangle ∥Y}\left(Y\right)-{ı}_{Q_{Y}∥P_{Y}}\left(Y\right)\right)\right], \end{array}$$
  where the expectations are with respect to $Y∼P_{Y}$, and
  - (107) follows from the log-sum inequality for integrable non-negative random variables,
    (109) $$\begin{array}{c} E\left[V\right]\log\frac{E[V]}{E[W]}\leq E\left[V\log\frac{V}{W}\right]; \end{array}$$
  - (108) ⇐ (100) and (101).
  Taking expectation with respect to $X∼P_{X}$ of (106)–(108) yields (99) because of Lemma A1 and (105). If α≥1, then Jensen’s inequality applied to the right side of (94) results in (98) but with the opposite inequality. Moreover, (107) is reversed and the remainder of the proof holds verbatim. □
  In the case of finite input-alphabets, a different proof of (99) is given in Appendix B of [54].
29. Introduced in the unpublished dissertation [36] and rescued from oblivion in [32], the Augustin–Csiszár mutual information of order α is defined for α>0 as
  (110) $$\begin{array}{cc} I_{\alpha }^{c}\left(X;Y\right)=I_{\alpha }^{c}\left(P_{X},P_{Y|X}\right)= & \min_{Q_{Y}}E\left[D_{\alpha }\left(P_{Y|X}\left(\cdot |X\right)\;∥\;Q_{Y}\right)\right] \end{array}$$
  (111) $$\begin{array}{cc} \; & =E\left[D_{\alpha }\left(P_{Y|X}\left(\cdot |X\right)\;∥\;P_{Y\langle \alpha \rangle }\right)\right], \end{array}$$
  where (111) follows from (98) if α∈(0,1], and from the reverse of (99) if α≥1. We conform to the notation in [40], where $I_{\alpha }^{a}$ was used to denote the difference between entropy and Arimoto-Rényi conditional entropy. In [32,39,43] the Augustin–Csiszár mutual information of order α is denoted by $I_{\alpha }$. In Augustin’s original notation [36], $I^{\rho }\left(P_{X}\right)$ means $I_{1-\rho }^{c}\left(P_{X},P_{Y|X}\right)$, ρ∈(0,1). Independently of [36], Poltyrev [35] introduced a functional (expressed as a maximization over a reverse random transformation) which turns out to be $\rho I_{\frac{1}{1+\rho }}^{c}\left(X;Y\right)$ and which he denoted by $E_{0}\left(\rho ,P_{X}\right)$, although in Gallager’s notation that corresponds to $\rho I_{\frac{1}{1+\rho }}\left(X;Y\right)$, as we will see in (233). $I_{0}^{c}\left(X;Y\right)$ and $I_{\infty }^{c}\left(X;Y\right)$ are defined by taking the corresponding limits.
30. In the discrete case, (110) boils down to
  (112) $$\begin{array}{c} I_{\alpha }^{c}\left(X;Y\right)=\min_{Q_{Y}}\frac{1}{\alpha -1}\sum_{x\in A}P_{X}\left(x\right)\log\sum_{y\in B}P_{Y|X}^{\alpha }\left(y|x\right)\;Q_{Y}^{1-\alpha }\left(y\right), \end{array}$$
  which can be juxtaposed with the much easier expression in (82) for $I_{\alpha }\left(X;Y\right)$ involving no further optimization. Minimizing the Lagrangian, we can verify that the minimizer in (112) satisfies (93). With $\left(X,\bar{Y}\right)∼P_{X}\times Q_{Y}$, we have
  (113) $$\begin{array}{cc} I_{0}^{c}\left(X;Y\right) & =\min_{Q_{Y}}E\left[\log\frac{1}{P[P_{Y|X}\left(\bar{Y}|X\right)>0|X]}\right], \end{array}$$
  (114) $$\begin{array}{cc} I_{\infty }^{c}\left(X;Y\right) & =\min_{Q_{Y}}E\left[\log{∥\frac{P_{Y|X}\left(\bar{Y}|X\right)}{Q_{Y}\left(\bar{Y}\right)}∥}_{\infty }\right], \end{array}$$
  where the expectations are with respect to *X*.
31. The respective minimizers of (72) and (110), namely, the α-response and the 〈α〉-response, are quite different. Most notably, in contrast to Item 7, an explicit expression for $P_{Y\langle \alpha \rangle }$ is unknown. Instead of defining $P_{Y\langle \alpha \rangle }$ through (92), [36] defines it, equivalently, as the fixed point of the operator (dubbed the Augustin operator in [43]) which maps the set of probability measures on the output space to itself,
  (115) $$\begin{array}{c} \frac{dT_{\alpha }\left(Q\right)}{dQ}\left(y\right)=E\left[{\left(\frac{dP_{Y|X}}{dQ}\left(y|X\right)\right)}^{\alpha }\exp\left(\left(1-\alpha \right)D_{\alpha }\left(P_{Y|X}\left(\cdot |X\right)∥Q\right)\right)\right], \end{array}$$
  where $X∼P_{X}$. Although we do not rely on them, Lemma 34.2 of (α∈(0,1)) and Lemma 13 of [43] (α>1) claim that the minimizer in (110), referred to in [43] as the Augustin mean of order α, is unique and is a fixed point of the operator $T_{\alpha }$ regardless of $P_{X}$. Moreover, Lemma 13(c) of [43] establishes that for α∈(0,1) and finite input alphabets, repeated iterations of the operator $T_{\alpha }$ with initial argument $P_{Y[\alpha ]}$ converge to $P_{Y\langle \alpha \rangle }$.
32. It is interesting to contrast the next example with the formulas in Examples 2 and 6.
  **Example** **7.** *Additive independent Gaussian noise.* *If Y=X+N, where $X∼N\;\left(0,\sigma_{X}^{2}\right)$ independent of $N∼N\;\left(0,\sigma_{N}^{2}\right)$, then*
  (116) $$\begin{array}{cc} Y\langle \alpha \rangle & ∼N\;\left(0,\frac{\sigma_{N}^{2}}{2}\left(2-\frac{1}{\alpha }+\Delta +\mathrm{snr}\right)\right), \end{array}$$
  (117) $$\begin{array}{cc} \mathrm{snr} & =\frac{\sigma_{X}^{2}}{\sigma_{N}^{2}}, \end{array}$$
  (118) $$\begin{array}{cc} \Delta & =\sqrt{4\;\mathrm{snr}+{\left(\frac{1}{\alpha }-\mathrm{snr}\right)}^{2}}. \end{array}$$
*This result can be obtained by postulating a zero-mean Gaussian distribution with variance $v_{\alpha }^{2}$ as $P_{Y\langle \alpha \rangle }$ and verifying that (92) is indeed satisfied if $v_{\alpha }^{2}$ is chosen as in (116). The first step is to invoke (32), which yields*
  (119) $$\begin{array}{cc} D_{\alpha }\left(P_{Y|X=x}\;∥\;P_{Y\langle \alpha \rangle }\right) & =\frac{\lambda_{\alpha }}{2}+\frac{\alpha \;x^{2}}{2\;s_{\alpha }^{2}}\log e, \end{array}$$
  (120) $$\begin{array}{cc} \lambda_{\alpha } & =\log\frac{v_{\alpha }^{2}}{\sigma_{N}^{2}}+\frac{1}{\alpha -1}\log\frac{v_{\alpha }^{2}}{s_{\alpha }^{2}}, \end{array}$$
  *where we have denoted $s_{\alpha }^{2}=\alpha \;v_{\alpha }^{2}+\left(1-\alpha \right)\sigma_{N}^{2}$. Since $Y∼N\;\left(0,\sigma_{X}^{2}+\sigma_{N}^{2}\right)$,*
  (121) $$\begin{array}{cc} {ı}_{X;Y}\left(x;y\right) & =\frac{1}{2}\log\frac{\sigma_{X}^{2}+\sigma_{N}^{2}}{\sigma_{N}^{2}}+\frac{1}{2}\left(\frac{y^{2}}{\sigma_{X}^{2}+\sigma_{N}^{2}}-\frac{{\left(y-x\right)}^{2}}{\sigma_{N}^{2}}\right)\log e, \end{array}$$
  (122) $$\begin{array}{cc} {ı}_{Y\langle \alpha \rangle ∥Y}\left(y\right) & =\frac{1}{2}\log\frac{\sigma_{X}^{2}+\sigma_{N}^{2}}{v_{\alpha }^{2}}+\frac{1}{2}\left(\frac{y^{2}}{\sigma_{X}^{2}+\sigma_{N}^{2}}-\frac{y^{2}}{v_{\alpha }^{2}}\right)\log e. \end{array}$$
*Assembling (120) and (121), the right side of (92) becomes*
  (123) $$\begin{array}{cc} \; & \frac{1}{\alpha }\log E\left[\exp(\alpha \;{ı}_{X;Y}\left(X;y\right)+\left(1-\alpha \right)D_{\alpha }\left(P_{Y|X}\left(\cdot |X\right)\;∥\;P_{Y\langle \alpha \rangle }\right)\right]\\ \; & =\frac{1}{2}\log\frac{\sigma_{X}^{2}+\sigma_{N}^{2}}{\sigma_{N}^{2}}+\frac{1}{2}\frac{y^{2}\log e}{\sigma_{X}^{2}+\sigma_{N}^{2}}+\frac{1-\alpha }{2\alpha }\lambda_{\alpha }\\ \; & \;+\frac{1}{\alpha }\log E\left[\exp_{e}\left(-\frac{\alpha {\left(y-X\right)}^{2}}{2\sigma_{N}^{2}}+\frac{\alpha \left(1-\alpha \right)X^{2}}{2s_{\alpha }^{2}}\right)\right] \end{array}$$
  (124) $$\begin{array}{cc} \; & =\frac{1}{2}\log\frac{\sigma_{X}^{2}+\sigma_{N}^{2}}{\sigma_{N}^{2}}+\frac{1-\alpha }{2\alpha }\lambda_{\alpha }+\frac{y^{2}\log e}{2}\left(\frac{1}{\sigma_{X}^{2}+\sigma_{N}^{2}}-\frac{s_{\alpha }^{2}-\alpha \left(1-\alpha \right)\sigma_{X}^{2}}{\sigma_{N}^{2}s_{\alpha }^{2}+\alpha^{2}v_{\alpha }^{2}\sigma_{X}^{2}}\right)\\ \; & \;+\frac{1}{2\alpha }\log\frac{\sigma_{N}^{2}s_{\alpha }^{2}}{\sigma_{N}^{2}s_{\alpha }^{2}+\alpha^{2}v_{\alpha }^{2}\sigma_{X}^{2}} \end{array}$$
  (125) $$\begin{array}{cc} \; & =\frac{1}{2}\log\frac{\sigma_{X}^{2}+\sigma_{N}^{2}}{v_{\alpha }^{2}}+\frac{1}{2}\left(\frac{y^{2}}{\sigma_{X}^{2}+\sigma_{N}^{2}}-\frac{y^{2}}{v_{\alpha }^{2}}\right)\log e, \end{array}$$
  *where (124) follows by Gaussian integration, and the marvelous simplification in (125) is satisfied provided that we choose*
  (126) $$\begin{array}{c} s_{\alpha }^{2}=\frac{\alpha \;\sigma_{X}^{2}\;v_{\alpha }^{2}}{v_{\alpha }^{2}-\sigma_{N}^{2}}. \end{array}$$
*Comparing (122) and (125), we see that (92) is indeed satisfied with $Y\langle \alpha \rangle ∼N\;\left(0,v_{\alpha }^{2}\right)$ if $v_{\alpha }^{2}$ satisfies the quadratic Equation (126), whose solution is in (116)–(118). Invoking (32) and (116), we obtain*
  (127) $$\begin{array}{cc} I_{\alpha }^{c}\left(X;X+N\right) & =\frac{\alpha \;\mathrm{snr}}{1+\alpha \;\Delta +\alpha \;\mathrm{snr}}\log e+\frac{1}{2}\log\left(1+\frac{1}{2}\left(\Delta +\mathrm{snr}-\frac{1}{\alpha }\right)\right)\\ \; & \;-\frac{1}{2(1-\alpha )}\log\frac{2-\frac{1}{\alpha }+\Delta +\mathrm{snr}}{1+\alpha \;\Delta +\alpha \;\mathrm{snr}}. \end{array}$$
  Beyond its role in evaluating the Augustin–Csiszár mutual information for Gaussian inputs, the Gaussian distribution in (116) has found some utility in the analysis of finite blocklength fundamental limits for data transmission [55].
33. This item gives a variational representation for the Augustin–Csiszár mutual information in terms of mutual information and conditional relative entropy (i.e., non-Rényi information measures). As we will see in Section 10, this representation accounts for the role played by Augustin–Csiszár mutual information in expressing error exponent functions.
  **Theorem** **8.** *For α∈(0,1), the Augustin–Csiszár mutual information satisfies the variational representation in terms of conditional relative entropy and mutual information,*
  (128) $$\begin{array}{c} \left(1-\alpha \right)\;I_{\alpha }^{c}\left(P_{X},P_{Y|X}\right)=\min_{Q_{Y|X}}\left\{\alpha \;D(Q_{Y|X}∥P_{Y|X}\left|P_{X}\right)+\left(1-\alpha \right)\;I\left(P_{X},Q_{Y|X}\right)\right\}, \end{array}$$
*where the minimum is over all the random transformations from the input to the output spaces.*
  **Proof.**  Invoking (47) with $\left(P_{1},P_{0}\right)\leftarrow \left(P_{Y|X=x},Q_{Y}\right)$ we obtain
  (129) $$\begin{array}{cc} \left(1-\alpha \right)\;D_{\alpha }\left(P_{Y|X=x}∥Q_{Y}\right)\; & =\min_{R_{Y}}\left\{\alpha \;D\left(R_{Y}∥P_{Y|X=x}\right)+\left(1-\alpha \right)\;D\left(R_{Y}∥Q_{Y}\right)\right\} \end{array}$$
  (130) $$\begin{array}{cc} \; & \;=\min_{R_{Y|X=x}}\left\{\alpha \;D\left(R_{Y|X=x}∥P_{Y|X=x}\right)+\left(1-\alpha \right)\;D\left(R_{Y|X=x}∥Q_{Y}\right)\right\}. \end{array}$$
  Averaging over $x∼P_{X}$, followed by minimization with respect to $Q_{Y}$ yields (128) upon recalling (67). □
  In the finite-alphabet case with α∈(0,1)∪(1,∞), the representation in (128) is implicit in the appendix of [32], and stated explicitly in [39], where it is shown by means of a minimax theorem. This is one of the instances in which the proof of the result is considerably easier for α∈(0,1); we can take the following route to show (128) for α>1. Neglecting to emphasize its dependence on $P_{X}$, denote
  (131) $$\begin{array}{c} f_{\alpha }\left(Q_{Y},R_{Y|X}\right)=\frac{\alpha }{1-\alpha }\;D(R_{Y|X}∥P_{Y|X}|P_{X})+D(R_{Y|X}∥Q_{Y}\left|P_{X}\right). \end{array}$$
  Invoking (47) we obtain
  (132) $$\begin{array}{cc} D_{\alpha }\left(P_{Y|X=x}∥Q_{Y}\right) & =\max_{R_{Y|X=x}}\left\{\frac{\alpha }{1-\alpha }\;D\left(R_{Y|X=x}∥P_{Y|X=x}\right)+D\left(R_{Y|X=x}∥Q_{Y}\right)\right\}. \end{array}$$
  Averaging (132) with respect to $P_{X}$ followed by minimization over $Q_{Y}$, results in
  (133) $$\begin{array}{cc} I_{\alpha }^{c}\left(P_{X},P_{Y|X}\right) & =\min_{Q_{Y}}\max_{R_{Y|X}}f_{\alpha }\left(Q_{Y},R_{Y|X}\right) \end{array}$$
  (134) $$\begin{array}{cc} \; & \geq \max_{R_{Y|X}}\min_{Q_{Y}}f_{\alpha }\left(Q_{Y},R_{Y|X}\right) \end{array}$$
  (135) $$\begin{array}{cc} \; & =\max_{R_{Y|X}}\left\{\frac{\alpha }{1-\alpha }\;D(R_{Y|X}∥P_{Y|X}\left|P_{X}\right)+I\left(P_{X},R_{Y|X}\right)\right\}, \end{array}$$
  which shows ≥ in (128). If a minimax theorem can be invoked to show equality in (134), then (128) is established for α>1. For that purpose, for fixed $R_{Y|X}$, $f(\cdot ,R_{Y|X})$ is convex and lower semicontinuous in $Q_{Y}$ on the set where it is finite. Rewriting
  (136) $$\begin{array}{c} f(Q_{Y},R_{Y|X})\\ =\frac{1}{1-\alpha }\;D(R_{Y|X}∥P_{Y|X}|P_{X})+D(R_{Y|X}∥Q_{Y}|P_{X})-D(R_{Y|X}∥P_{Y|X}\left|P_{X}\right), \end{array}$$
  it can be seen that $f(Q_{Y},\cdot )$ is upper semicontinuous and concave (if α>1). A different, and considerably more intricate route is taken in Lemma 13(d) of [43], which also gives (128) for α>1 assuming finite input alphabets.
34. Unlike mutual information, neither $I_{\alpha }\left(X;Y\right)=I_{\alpha }\left(Y;X\right)$ nor $I_{\alpha }^{c}\left(X;Y\right)=I_{\alpha }^{c}\left(Y;X\right)$ hold in general.
  **Example** **8.** *Erasure transformation. Let A={0,1},B={0,1,e},*
  (137) $$P_{Y|X}\left(b|a\right)=\left\{\begin{array}{cc} 1-\delta , & a=b;\\ \delta , & b=e;\\ 0, & a\neq b\neq e, \end{array}\right.$$
*with δ∈(0,1), and $P_{X}\left(0\right)=\frac{1}{2}$. Then, we obtain, for α∈(0,1)∪(1,∞),*
  (138) $$\begin{array}{cc} I_{\alpha }\left(X;Y\right) & =I_{\alpha }^{c}\left(X;Y\right)=\frac{\alpha }{\alpha -1}\log\left(\delta +\left(1-\delta \right)\;2^{\left(1-\frac{1}{\alpha }\right)}\right), \end{array}$$
  (139) $$\begin{array}{cc} I_{\alpha }\left(Y;X\right) & =\frac{1}{\alpha -1}\log\left(\delta +\left(1-\delta \right)\;2^{\alpha -1}\right), \end{array}$$
  (140) $$\begin{array}{cc} I_{\alpha }^{c}\left(Y;X\right) & =I(X;Y)=1-\delta \;bits. \end{array}$$
35. It was shown in Theorem 5.2 of [38] that α-mutual information satisfies the data processing lemma, namely, if *X* and *Z* are conditionally independent given *Y*, then
  (141) $$\begin{array}{cc} I_{\alpha }\left(X;Z\right) & \leq \min\left\{I_{\alpha }\left(X;Y\right),I_{\alpha }\left(Y;Z\right)\right\}, \end{array}$$
  (142) $$\begin{array}{cc} I_{\alpha }\left(Z;X\right) & \leq \min\left\{I_{\alpha }\left(Z;Y\right),I_{\alpha }\left(Y;X\right)\right\}. \end{array}$$
  As shown by Csiszár [32] using the data processing inequality for Rényi divergence, the data processing lemma also holds for $I_{\alpha }^{c}$.
36. From (53), (54) and the monotonicity of $D_{\alpha }\left(P∥Q\right)$ in α, we obtain the ordering
  (143) $$\begin{array}{cc} I_{\beta }\left(X;Y\right)\leq I_{\alpha }\left(X;Y\right) & \leq I_{\alpha }^{c}\left(X;Y\right)\leq I_{\nu }^{c}\left(X;Y\right)\leq I\left(X;Y\right),\;0<\beta \leq \alpha \leq \nu <1; \end{array}$$
  (144) $$\begin{array}{cc} I\left(X;Y\right)\leq I_{\nu }^{c}\left(X;Y\right) & \leq I_{\alpha }^{c}\left(X;Y\right)\leq I_{\alpha }\left(X;Y\right)\leq I_{\beta }\left(X;Y\right),\;1<\nu \leq \alpha \leq \beta . \end{array}$$
37. The convexity/concavity properties of the generalized mutual informations are summarized next.
  **Theorem** **9.**
  (*a*) *$\rho \;I_{\frac{1}{1+\rho }}\left(X;Y\right)$ and $\rho \;I_{\frac{1}{1+\rho }}^{c}\left(X;Y\right)$ are concave and monotonically non-decreasing in ρ≥0.*
  (*b*) *$I(\cdot ,P_{Y|X})$ and $I_{\alpha }^{c}\left(\cdot ,P_{Y|X}\right)$ are concave functions. The same holds for $I_{\alpha }\left(\cdot ,P_{Y|X}\right)$ if α>1.*
  (*c*) *If α∈(0,1), then $I(P_{X},\cdot )$, $I_{\alpha }\left(P_{X},\cdot \right)$ and $I_{\alpha }^{c}\left(P_{X},\cdot \right)$ are convex functions.*
  **Proof.** 
  (a) According to (81) and (128), respectively, with $\alpha =\frac{1}{1+\rho }\in \left(0,1\right)$, $\rho \;I_{\frac{1}{1+\rho }}\left(X;Y\right)$ and $\rho \;I_{\frac{1}{1+\rho }}^{c}\left(X;Y\right)$ are the infima of affine functions with nonnegative slopes.
  (b) For mutual information the result goes back to [56] in the finite-alphabet case. In general, it holds since (67) is the infimum of linear functions of $P_{X}$. The same reasoning applies to Augustin–Csiszár mutual information in view of (110). For α-mutual information with α>1, notice from (51) that $D_{\alpha }(P_{Y|X}\;∥\;Q_{Y}\left|P_{X}\right)$ is concave in $P_{X}$ if α>1. Therefore,
    (145) $$\begin{array}{cc} \; & \;I_{\alpha }\left(\lambda \;P_{X}^{1}+\left(1-\lambda \right)\;P_{X}^{0},P_{Y|X}\right) \end{array}$$
    (146) $$\begin{array}{cc} \; & =\underset{Q_{Y}}{\inf}D_{\alpha }(P_{Y|X}\;∥\;Q_{Y}\left|\;\lambda \;P_{X}^{1}+\left(1-\lambda \right)\;P_{X}^{0}\right) \end{array}$$
    (147) $$\begin{array}{cc} \; & \geq \underset{Q_{Y}}{\inf}\lambda \;D_{\alpha }(P_{Y|X}\;∥\;Q_{Y}|\;P_{X}^{1})+\left(1-\lambda \right)\;D_{\alpha }(P_{Y|X}\;∥\;Q_{Y}\left|\;P_{X}^{0}\right) \end{array}$$
    (148) $$\begin{array}{cc} \; & \geq \lambda \;I_{\alpha }\left(P_{X}^{1},P_{Y|X}\right)+\left(1-\lambda \right)\;I_{\alpha }\left(P_{X}^{0},P_{Y|X}\right). \end{array}$$
  (c) The convexity of $I(P_{X},\cdot )$ and $I_{\alpha }\left(P_{X},\cdot \right)$ follow from the convexity of $D_{\alpha }\left(P∥Q\right)$ in (P,Q) for α∈(0,1] as we saw in Item 18. To show convexity of $I_{\alpha }^{c}\left(P_{X},\cdot \right)$ if α∈(0,1), we apply (169) in Item 45 with $P_{Y|X}=\lambda P_{Y|X}^{1}+\left(1-\lambda \right)P_{Y|X}^{0}$, and invoke the convexity of $I_{\alpha }\left(P_{X},\cdot \right)$:
    $$\begin{array}{cc} \; & \;\left(1-\alpha \right)\;I_{\alpha }^{c}\left(P_{X},P_{Y|X}\right) \end{array}$$
    (149) $$\begin{array}{cc} \; & =\max_{Q_{X}}\left\{\left(1-\alpha \right)\;I_{\alpha }\left(Q_{X},\lambda P_{Y|X}^{1}+\left(1-\lambda \right)P_{Y|X}^{0}\right)-D\left(P_{X}\;∥\;Q_{X}\right)\right\},\\ \; & \leq \max_{Q_{X}}\left\{\lambda \left(1-\alpha )\;I_{\alpha }\left(Q_{X},P_{Y|X}^{1}\right)-D\left(P_{X}\;∥\;Q_{X}\right)\right)\right. \end{array}$$
    (150) $$\begin{array}{cc} \; & \left.\;+\left(1-\lambda \right)\left(1-\alpha )\;I_{\alpha }\left(Q_{X},P_{Y|X}^{0}\right)-D\left(P_{X}\;∥\;Q_{X}\right)\right)\right\} \end{array}$$
    (151) $$\begin{array}{cc} \; & \leq \left(1-\alpha \right)\left(\lambda I_{\alpha }^{c}\left(P_{X},P_{Y|X}^{1}\right)+\left(1-\lambda \right)I_{\alpha }^{c}\left(P_{X},P_{Y|X}^{0}\right)\right). \end{array}$$
  □
  Although not used in the sequel, we note, for completeness, that if α∈(0,1)∪(1,∞), [38] (see corrected version in [41]) shows that $\exp\left(\left(1-\frac{1}{\alpha }\right)I_{\alpha }\left(\cdot ,P_{Y|X}\right)\right)/\left(\alpha -1\right)$ is concave.

## 5. Interplay between $I_{\alpha }\left(P_{X},P_{Y|X}\right)$ and $I_{\alpha }^{c}\left(P_{X},P_{Y|X}\right)$

In this section we study the interplay between both notions of mutual informations of order α, and, in particular, various variational representations of these information measures.

38. For given α∈(0,1)∪(1,∞) and $P_{Y|X}:A\to B$, define $Q_{X[\alpha ]}\ll \gg P_{X}$, the α-adjunct of $P_{X}$ by
  (152) $$\begin{array}{c} {ı}_{Q_{X[\alpha ]}∥P_{X}}\left(x\right)=\left(\alpha -1\right)\;D_{\alpha }\left(P_{Y|X=x}∥P_{Y[\alpha ]}\right)-\kappa_{\alpha }, \end{array}$$
  with $\kappa_{\alpha }$ the constant in (14) and $P_{Y[\alpha ]}$, the α-response to $P_{X}$.
39.
  **Example** **9.** *Let Y=X+N with $X∼N\;\left(0,\sigma_{X}^{2}\right)$ independent of $N∼N\;\left(0,\sigma_{N}^{2}\right)$, and $\mathrm{snr}=\frac{\sigma_{X}^{2}}{\sigma_{N}^{2}}$. The α-adjunct of the input is*
  (153) $$\begin{array}{c} Q_{X[\alpha ]}=N\;\left(0,\sigma_{X}^{2}\frac{1+\alpha^{2}\;\mathrm{snr}}{1+\alpha \;\mathrm{snr}}\right). \end{array}$$
40.
  **Theorem** **10.** *The 〈α〉-response to $Q_{X[\alpha ]}$ is $P_{Y[\alpha ]}$, the α-response to $P_{X}$.*
  **Proof.**  We just need to verify that (92) is satisfied if we substitute Y〈α〉 by Y[α], and instead of taking the expectation in the right side with respect to $X∼P_{X}$ we take it with respect to $\tilde{X}∼Q_{X[\alpha ]}$. Then,
  $$\begin{array}{cc} \; & E\left[\exp(\alpha \;{ı}_{X;Y}\left(\tilde{X};y\right)+\left(1-\alpha \right)D_{\alpha }\left(P_{Y|X}\left(\cdot |\tilde{X}\right)\;∥\;P_{Y[\alpha ]}\right)\right] \end{array}$$
  (154) $$\begin{array}{cc} \; & =E\left[\exp\left({ı}_{Q_{X[\alpha ]}∥P_{X}}\left(X\right)+\alpha \;{ı}_{X;Y}\left(X;y\right)+\left(1-\alpha \right)D_{\alpha }\left(P_{Y|X}\left(\cdot |X\right)\;∥\;P_{Y[\alpha ]}\right)\right)\right] \end{array}$$
  (155) $$\begin{array}{cc} \; & =E\left[\exp(\alpha \;{ı}_{X;Y}\left(X;y\right)-\kappa_{\alpha })\right] \end{array}$$
  (156) $$\begin{array}{cc} \; & =\exp\left(\alpha \;{ı}_{Y[\alpha ]∥Y}\left(y\right)\right), \end{array}$$
  where (154) is by change of measure, (155) follows by substitution of (152), and (156) is the same as (13). □
41. For given α∈(0,1)∪(1,∞) and $P_{Y|X}:A\to B$, we define $Q_{X\langle \alpha \rangle }\ll \gg P_{X}$, the 〈α〉-adjunct of an input probability measure $P_{X}$ through
  (157) $$\begin{array}{c} {ı}_{Q_{X\langle \alpha \rangle }∥P_{X}}\left(x\right)=\left(1-\alpha \right)\;D_{\alpha }\left(P_{Y|X=x}\;∥\;P_{Y\langle \alpha \rangle }\right)+\upsilon_{\alpha }, \end{array}$$
  where $P_{Y\langle \alpha \rangle }$ is the 〈α〉-response to $P_{X}$ and $\upsilon_{\alpha }$ is a normalizing constant so that $Q_{X\langle \alpha \rangle }$ is a probability measure. According to (9), we must have
  (158) $$\begin{array}{c} E\left[\exp\left({ı}_{Q_{X\langle \alpha \rangle }∥P_{X}}\left(X\right)\right)\right]=1,\;X∼P_{X}. \end{array}$$
  Hence,
  (159) $$\begin{array}{cc} \upsilon_{\alpha } & =\left(\alpha -1\right)\;D_{\alpha }\left(P_{Y|X}\;∥\;P_{Y\langle \alpha \rangle }\;|\;Q_{X\langle \alpha \rangle }\right). \end{array}$$
42. With the aid of the expression in Example 7, we obtain
  **Example** **10.** *Let Y=X+N with $X∼N\;\left(0,\sigma_{X}^{2}\right)$ independent of $N∼N\;\left(0,\sigma_{N}^{2}\right)$, and $\mathrm{snr}=\frac{\sigma_{X}^{2}}{\sigma_{N}^{2}}$. Then, the 〈α〉-adjunct of the input is*
  (160) $$\begin{array}{c} Q_{X\langle \alpha \rangle }=N\;\left(0,\sigma_{X}^{2}\;\frac{1+\alpha (\Delta +\mathrm{snr})}{1+\alpha \left(\Delta -\mathrm{snr}\right)+2\;\alpha^{2}\;\mathrm{snr}}\right), \end{array}$$
  *which, in contrast to $Q_{X[\alpha ]}$, has larger variance than $\sigma_{X}^{2}$ if α∈(0,1).*
43. The following result is the dual of Theorem 10.
  **Theorem** **11.** *The α-response to $Q_{X\langle \alpha \rangle }$ is $P_{Y\langle \alpha \rangle }$, the 〈α〉-response to $P_{X}$. Therefore,*
  (161) $$\begin{array}{cc} \upsilon_{\alpha }\; & =\left(\alpha -1\right)\;I_{\alpha }\left(Q_{X\langle \alpha \rangle },P_{Y|X}\right). \end{array}$$
  **Proof.**  The proof is similar to that of Theorem 10. We just need to verify that we obtain the right side of (92) if on the right side of (91) we substitute $P_{X}$ by $Q_{X\langle \alpha \rangle }$ and $P_{Y[\alpha ]}$ by $P_{Y\langle \alpha \rangle }$. Let $\bar{X}∼Q_{X\langle \alpha \rangle }$. Then,
  $$\begin{array}{cc} \; & \frac{1}{\alpha }\log E\left[\exp\left(\alpha \;{ı}_{X;Y}\left(\bar{X};y\right)+\left(1-\alpha \right)D_{\alpha }\left(P_{Y|X}\;∥\;P_{Y\langle \alpha \rangle }|Q_{X\langle \alpha \rangle }\right)\right)\right] \end{array}$$
  (162) $$\begin{array}{cc} \; & =\frac{1}{\alpha }\log E\left[\exp\left({ı}_{Q_{X\langle \alpha \rangle }∥P_{X}}\left(X\right)+\alpha \;{ı}_{X;Y}\left(X;y\right)-\upsilon_{\alpha }\right)\right] \end{array}$$
  (163) $$\begin{array}{cc} \; & =\frac{1}{\alpha }\log E\left[\exp\left(\alpha \;{ı}_{X;Y}\left(X;y\right)+\left(1-\alpha \right)D_{\alpha }\left(P_{Y|X}\left(\cdot |X\right)\;∥\;P_{Y\langle \alpha \rangle }\right)\right)\right] \end{array}$$
  (164) $$\begin{array}{cc} \; & ={ı}_{Y\langle \alpha \rangle ∥Y}\left(y\right), \end{array}$$
  where (162)–(164) follow by change of measure, (157), and (92), respectively. □
44. By recourse to a minimax theorem, the following representation is given for α∈(0,1)∪(1,∞) in the case of finite alphabets in [39], and dropping the restriction on the finiteness of the output space in [43]. As we show, a very simple and general proof is possible for α∈(0,1).
  **Theorem** **12.** *Fix α∈(0,1), $P_{X}\in P_{A}$ and $P_{Y|X}:A\to B$. Then,*
  (165) $$\begin{array}{c} \left(1-\alpha \right)\;I_{\alpha }\left(X;Y\right)=\min_{Q_{X}}\left\{\left(1-\alpha \right)\;I_{\alpha }^{c}\left(Q_{X},P_{Y|X}\right)+D\left(Q_{X}\;∥\;P_{X}\right)\right\}, \end{array}$$
  *where the minimum is attained by $Q_{X[\alpha ]}$, the α-adjunct of $P_{X}$ defined in (152).*
  **Proof.**  The variational representations in (81) and (128) result in (165). To show that the minimum is indeed attained by $Q_{X[\alpha ]}$, recall from Theorem 10 that the 〈α〉-response to $Q_{X[\alpha ]}$ is $P_{Y[\alpha ]}$. Therefore, evaluating the term in {} in (165) for $Q_{X}\leftarrow Q_{X[\alpha ]}$ yields, with $\tilde{X}∼Q_{X[\alpha ]}$,
  $$\begin{array}{cc} \; & \;\left(1-\alpha \right)\;I_{\alpha }^{c}\left(Q_{X[\alpha ]},P_{Y|X}\right)+D\left(Q_{X[\alpha ]}\;∥\;P_{X}\right) \end{array}$$
  (166) $$\begin{array}{cc} \; & =\left(1-\alpha \right)\;E\left[D_{\alpha }\left(P_{Y|X}\left(\cdot |\tilde{X}\right)\;∥\;P_{Y[\alpha ]}\right)\right]+D\left(Q_{X[\alpha ]}\;∥\;P_{X}\right) \end{array}$$
  (167) $$\begin{array}{cc} \; & =-\kappa_{\alpha } \end{array}$$
  (168) $$\begin{array}{cc} \; & =\left(1-\alpha \right)\;I_{\alpha }\left(X;Y\right), \end{array}$$
  where (167) follows from (152) and (168) results from (69)–(75). □
45. For finite-input alphabets, Lemma 18(b) of [43] (earlier Theorem 3.4 of [35] gave an equivalent variational characterization assuming, in addition, finite output alphabets) established the following dual to Theorem 12.
  **Theorem** **13.** *Fix α∈(0,1), $P_{X}\in P_{A}$ and $P_{Y|X}:A\to B$. Then,*
  (169) $$\begin{array}{c} \left(1-\alpha \right)\;I_{\alpha }^{c}\left(X;Y\right)=\max_{Q_{X}}\left\{\left(1-\alpha \right)\;I_{\alpha }\left(Q_{X},P_{Y|X}\right)-D\left(P_{X}\;∥\;Q_{X}\right)\right\}. \end{array}$$
*The maximum is attained by $Q_{X\langle \alpha \rangle }$, the 〈α〉-adjunct of $P_{X}$ defined by (157).*
  **Proof.**  First observe that (165) implies that ≥ holds in (169). Second, the term in {} on the right side of (169) evaluated at $Q_{X}\leftarrow Q_{X\langle \alpha \rangle }$ becomes
  $$\begin{array}{cc} \; & \;\left(1-\alpha \right)\;I_{\alpha }\left(Q_{X\langle \alpha \rangle },P_{Y|X}\right)-D\left(P_{X}\;∥\;Q_{X\langle \alpha \rangle }\right) \end{array}$$
  (170) $$\begin{array}{cc} \; & =\left(1-\alpha \right)\;I_{\alpha }\left(Q_{X\langle \alpha \rangle },P_{Y|X}\right)+\left(1-\alpha \right)I_{\alpha }^{c}\left(P_{X},P_{Y|X}\right)+\upsilon_{\alpha } \end{array}$$
  (171) $$\begin{array}{cc} \; & =\left(1-\alpha \right)I_{\alpha }^{c}\left(P_{X},P_{Y|X}\right), \end{array}$$
  where (170) follows by taking the expectation of minus (157) with respect to $P_{X}$. Therefore, ≤ also holds in (169) and the maximum is attained by $Q_{X\langle \alpha \rangle }$, as we wanted to show. □
  Hinging on Theorem 8, Theorems 12 and 13 are given for α∈(0,1) which is the region of interest in the analysis of error exponents. Whenever, as in the finite-alphabet case, (128) holds for α>1, Theorems 12 and 13 also hold for α>1.
  Notice that since the definition of $Q_{X\langle \alpha \rangle }$ involves $P_{Y\langle \alpha \rangle }$, the fact that it attains the maximum in (169) does not bring us any closer to finding $I_{\alpha }^{c}\left(X;Y\right)$ for a specific input probability measure $P_{X}$. Fortunately, as we will see in Section 8, (169) proves to be the gateway to the maximization of $I_{\alpha }^{c}\left(X;Y\right)$ in the presence of input-cost constraints.
46. Focusing on the main range of interest, α∈(0,1), we can express (169) as
  (172) $$\begin{array}{cc} I_{\alpha }^{c}\left(P_{X},P_{Y|X}\right)\; & =\max_{Q_{X}}\left\{I_{\alpha }\left(Q_{X},P_{Y|X}\right)-\frac{1}{1-\alpha }D\left(P_{X}\;∥\;Q_{X}\right)\right\} \end{array}$$
  (173) $$\begin{array}{cc} \; & =\max_{\xi \geq 0}\left\{I\left(\xi \right)-\frac{\xi }{1-\alpha }\right\} \end{array}$$
  (174) $$\begin{array}{cc} \; & =I\left(\xi_{\alpha }\right)-\frac{\xi_{\alpha }}{1-\alpha }, \end{array}$$
  where we have defined the function (dependent on α, $P_{X}$, and $P_{Y|X}$)
  (175) $$I\left(\xi \right)=\max_{\begin{array}{c} Q_{X}:\\ D(P_{X}∥Q_{X})\leq \xi \end{array}}I_{\alpha }\left(Q_{X},P_{Y|X}\right),$$
  and $\xi_{\alpha }$ is the solution to
  (176) $$\begin{array}{c} \dot{I}\left(\xi_{\alpha }\right)=\frac{1}{1-\alpha }. \end{array}$$
  Recall that the maxima over the input distribution in (172) and (175) are attained by the 〈α〉-adjunct $Q_{X\langle \alpha \rangle }$ defined in Item 41.
47. At this point it is convenient to summarize the notions of input and output probability measures that we have defined for a given α, random transformation $P_{Y|X}$, and input probability measure $P_{X}$:
  - $P_{Y}$: The familiar output probability measure $P_{X}\to P_{Y|X}\to P_{Y}$, defined in Item 5.
  - $P_{Y[\alpha ]}$: The α-response to $P_{X}$, defined in Item 7. It is the unique achiever of the minimization in the definition of α-mutual information in (67).
  - $P_{Y\langle \alpha \rangle }$: The 〈α〉-response to $P_{X}$ defined in Item 26. It is the unique achiever of the minimization in the definition of Augustin–Csiszár α-mutual information in (110).
  - $Q_{X[\alpha ]}$: The α-adjunct of $P_{X}$, defined in (152). The 〈α〉-response to $Q_{X[\alpha ]}$ is $P_{Y[\alpha ]}$. Furthermore, $Q_{X[\alpha ]}$ achieves the minimum in (165).
  - $Q_{X\langle \alpha \rangle }$: The 〈α〉-adjunct of $P_{X}$, defined in (157). The α-response to $Q_{X\langle \alpha \rangle }$ is $P_{Y\langle \alpha \rangle }$. Furthermore, $Q_{X\langle \alpha \rangle }$ achieves the maximum in (169).

## 6. Maximization of $I_{\alpha }\left(X;Y\right)$

Just like the maximization of mutual information with respect to the input distribution yields the channel capacity (of course, subject to conditions [57]), the maximization of $I_{\alpha }\left(X;Y\right)$ and of $I_{\alpha }^{c}\left(X;Y\right)$ arises in the analysis of error exponents, as we will see in Section 10. A recent in-depth treatment of the maximization of α-mutual information is given in [45]. As we see most clearly in (82) for the discrete case, when it comes to its optimization, one advantage of $I_{\alpha }\left(X;Y\right)$ over I(X;Y) is that the input distribution does not affect the expression through its influence on the output distribution.

48. The maximization of α-mutual information is facilitated by the following result.
  **Theorem** **14 ([45]).** *Given α∈(0,1)∪(1,∞); a random transformation $P_{Y|X}:A\to B$; and, a convex set $P\subset P_{A}$, the following are equivalent.*
  (*a*)  $P_{X}^{*}\in P$ attains the maximal α-mutual information on P,
    (177) $$\begin{array}{c} I_{\alpha }\left(P_{X}^{*},P_{Y|X}\right)=\max_{P\in P}I_{\alpha }\left(P,P_{Y|X}\right)<\infty . \end{array}$$
  (*b*)  *For any $P_{X}\in P$, and any output distribution $Q_{Y}\in P_{B}$,*
    (178) $$\begin{array}{cc} D_{\alpha }(P_{Y|X}\;∥\;P_{Y[\alpha ]}^{*}\left|P_{X}\right)\; & \leq D_{\alpha }(P_{Y|X}\;∥\;P_{Y[\alpha ]}^{*}\left|P_{X}^{*}\right) \end{array}$$
    (179) $$\begin{array}{cc} \; & \leq D_{\alpha }(P_{Y|X}\;∥\;Q_{Y}\left|P_{X}^{*}\right), \end{array}$$
    where $P_{Y[\alpha ]}^{*}$ is the α-response to $P_{X}^{*}$.
*Moreover, if $P_{Y[\alpha ]}$ denotes the α-response to $P_{X}$, then*
  (180) $$\begin{array}{c} D_{\alpha }\left(P_{Y[\alpha ]}∥P_{Y[\alpha ]}^{*}\right)\leq I_{\alpha }\left(P_{X}^{*},P_{Y|X}\right)-I_{\alpha }\left(P_{X},P_{Y|X}\right)<\infty . \end{array}$$
  Note that, while $I_{\alpha }\left(\cdot ,P_{Y|X}\right)$ may not be maximized by a unique (or, in fact, by any) input distribution, the resulting α-response $P_{Y[\alpha ]}^{*}$ is indeed unique. If P is such that none of its elements attain the maximal $I_{\alpha }$, it is known [42,45] that the α-response to any asymptotically optimal sequence of input distributions converges to $P_{Y[\alpha ]}^{*}$. This is the counterpart of a result by Kemperman [58] concerning mutual information.
49. The following example appears in [45].
  **Example** **11.** *Let Y=X+N where $N∼N\;\left(0,\sigma_{N}^{2}\right)$ independent of X. Fix α∈(0,1) and P>0. Suppose that the set, $P\subset P_{A}$, of allowable input probability measures consists of those that satisfy the constraint*
  (181) $$\begin{array}{c} E\left[\exp_{e}\left(-\frac{\alpha \left(1-\alpha \right)X^{2}}{2\left(\alpha^{2}P+\sigma_{N}^{2}\right)}\right)\right]\geq \sqrt{\frac{\alpha^{2}P+\sigma_{N}^{2}}{\alpha \;P+\sigma_{N}^{2}}}. \end{array}$$
We can readily check that $X^{*}∼N\;\left(0,P\right)$ satisfies (181) with equality, and as we saw in Example 2, its α-response is $P_{Y[\alpha ]}^{*}=N\left(0,\alpha \;P+\sigma^{2}\right)$. Theorem 14 establishes that $P_{X}^{*}$ does indeed maximize the α-mutual information among all the distributions in P, yielding (recall Example 6)
  (182) $$\begin{array}{c} \max_{P_{X}\in P}I_{\alpha }\left(X;Y\right)=\frac{1}{2}\log\left(1+\frac{\alpha P}{\sigma^{2}}\right). \end{array}$$
*Curiously, if, instead of P defined by the constraint (181), we consider the more conventional $P=\{X:E\left[X^{2}\right]\leq P\}$, then the left side of (182) is unknown at present. Numerical evidence shows that it can exceed the right side by employing non-Gaussian inputs.*
50. Recalling (56) and (178) implies that if $P_{X}^{*}$ attains the finite maximal unconstrained α-mutual information and its α-response is denoted by $P_{Y[\alpha ]}^{*}$, then,
  (183) $$\begin{array}{c} \max_{X}I_{\alpha }\left(X;Y\right)=\max_{P\in P}I_{\alpha }\left(P,P_{Y|X}\right)=\max_{a\in A}D_{\alpha }\left(P_{Y|X=a}∥P_{Y[\alpha ]}^{*}\right), \end{array}$$
  which requires that $P_{X}^{*}\left(A_{\alpha }^{*}\right)=1$, with
  (184) $$\begin{array}{c} A_{\alpha }^{*}=\left\{x\in A:D_{\alpha }\left(P_{Y|X=x}∥P_{Y[\alpha ]}^{*}\right)=\max_{a\in A}D_{\alpha }\left(P_{Y|X=a}∥P_{Y[\alpha ]}^{*}\right)\right\}. \end{array}$$
  For discrete alphabets, this requires that if $x\notin A_{\alpha }^{*}$, then $P_{X}^{*}\left(x\right)=0$, which is tantamount to
  (185) $$\begin{array}{cc} \sum_{y\in B}P_{Y|X}^{\alpha }\left(y|x\right)\;E^{\frac{1-\alpha }{\alpha }}\left[P_{Y|X}^{\alpha }\left(y|X^{*}\right)\right]\; & \geq \exp\left(\frac{\alpha -1}{\alpha }I_{\alpha }\left(X^{*};Y^{*}\right)\right), \end{array}$$
  with equality for all x∈A such that $P_{X}^{*}\left(x\right)>0$. For finite-alphabet random transformations this observation is equivalent to Theorem 5.6.5 in [9].
51. Getting slightly ahead of ourselves, we note that, in view of (128), an important consequence of Theorem 15 below, is that, as anticipated in Item 25, the unconstrained maximization of $I_{\alpha }\left(X;Y\right)$ for α∈(0,1) can be expressed in terms of the solution to an optimization problem involving only conventional mutual information and conditional relative entropy. For ρ≥0,
  (186) $$\begin{array}{c} \rho \;\underset{X}{\sup}I_{\frac{1}{1+\rho }}\left(X;Y\right)=\underset{X}{\sup}\min_{Q_{Y|X}:A\to B}\left\{D(Q_{Y|X}∥P_{Y|X}\left|P_{X}\right)+\rho \;I\left(P_{X},Q_{Y|X}\right)\right\}. \end{array}$$

## 7. Unconstrained Maximization of $I_{\alpha }^{c}\left(X;Y\right)$

52. In view of the fact that it is much easier to determine the α-mutual information than the order-α Augustin–Csiszár information, it would be advantageous to show that the unconstrained maximum of $I_{\alpha }^{c}\left(X;Y\right)$ equals the unconstrained maximum of $I_{\alpha }\left(X;Y\right)$. In the finite-alphabet setting, in which it is possible to invoke a "minisup” theorem (e.g., see Section 7.1.7 of [59]), Csiszár [32] showed this result for α>0. The assumption of finite output alphabets was dropped in Theorem 1 of [42], and further generalized in Theorem 3 of the same reference. As we see next, for α∈(0,1), it is possible to give an elementary proof without restrictions on the alphabets.
  **Theorem** **15.** *Let α∈(0,1). If the suprema are over $P_{A}$, the set of all probability measures defined on the input space, then*
  (187) $$\begin{array}{c} \underset{X}{\sup}I_{\alpha }^{c}\left(X;Y\right)=\underset{X}{\sup}I_{\alpha }\left(X;Y\right). \end{array}$$
  **Proof.** In view of (143), ≥ holds in (187). To show ≤, we assume $\sup_{X}I_{\alpha }\left(X;Y\right)<\infty$ as, otherwise, there is nothing left to prove. The unconstrained maximization identity in (183) implies
  (188) $$\begin{array}{cc} \underset{X}{\sup}I_{\alpha }\left(X;Y\right)\; & =\underset{a\in A}{\sup}D_{\alpha }\left(P_{Y|X=a}∥P_{Y[\alpha ]}^{*}\right) \end{array}$$
  (189) $$\begin{array}{cc} \; & =\underset{P_{X}\in P}{\sup}E\left[D_{\alpha }\left(P_{Y|X}\left(\cdot |X\right)∥P_{Y[\alpha ]}^{*}\right)\right] \end{array}$$
  (190) $$\begin{array}{cc} \; & \geq \underset{Q\in Q}{\inf}\underset{P_{X}\in P}{\sup}E\left[D_{\alpha }\left(P_{Y|X}\left(\cdot |X\right)∥Q\right)\right] \end{array}$$
  (191) $$\begin{array}{cc} \; & \geq \underset{P_{X}\in P}{\sup}\underset{Q\in Q}{\inf}E\left[D_{\alpha }\left(P_{Y|X}\left(\cdot |X\right)∥Q\right)\right] \end{array}$$
  (192) $$\begin{array}{cc} \; & =\underset{X}{\sup}I_{\alpha }^{c}\left(X;Y\right), \end{array}$$
  where $P_{Y[\alpha ]}^{*}$ is the unique α-response to any input that achieves the maximal α-mutual information, and if there is no such input, it is the limit of the α-responses to any asymptotically optimal input sequence (Item 48). □
  Furthermore, if $\left\{X_{n}\right\}$ is asymptotically optimal for $I_{\alpha }$, i.e., $\lim_{n\to \infty }I_{\alpha }\left(X_{n};Y_{n}\right)=\sup_{X}I_{\alpha }\left(X;Y\right)$, then $\left\{X_{n}\right\}$ is also asymptotically optimal for $I_{\alpha }^{c}$ because for any δ>0, we can find *N*, such that for all n>N,
  (193) $$\begin{array}{cc} I_{\alpha }\left(X_{n};Y_{n}\right)+\delta \; & \geq \underset{a\in A}{\sup}D_{\alpha }\left(P_{Y|X=a}∥P_{Y[\alpha ]}^{*}\right) \end{array}$$
  (194) $$\begin{array}{cc} \; & \geq E\left[D_{\alpha }\left(P_{Y|X}\left(\cdot |X_{n}\right)∥P_{Y[\alpha ]}^{*}\right)\right] \end{array}$$
  (195) $$\begin{array}{cc} \; & \geq I_{\alpha }^{c}\left(X_{n};Y_{n}\right) \end{array}$$
  (196) $$\begin{array}{cc} \; & \geq I_{\alpha }\left(X_{n};Y_{n}\right). \end{array}$$

## 8. Maximization of $I_{\alpha }^{c}\left(X;Y\right)$ Subject to Average Cost Constraints

This section is at the heart of the relevance of Rényi information measures to error exponent functions.

53. Given α∈(0,1), $P_{Y|X}:A\to B$, a cost function b:A→[0,∞) and real scalar θ≥0, the objective is to maximize the Augustin–Csiszár mutual information allowing only those probability measures that satisfy E[b(X)]≤θ, namely,
  (197) $$C_{\alpha }^{c}\left(\theta \right)=\underset{\begin{array}{c} P_{X}:\\ E[b(X)]\leq \theta \end{array}}{\sup}\;\;\;\;\;\;I_{\alpha }^{c}\left(P_{X},P_{Y|X}\right).$$
  Unfortunately, identity (187) no longer holds when the maximizations over the input probability measure are cost-constrained, and, in general, we can only claim
  (198) $$C_{\alpha }^{c}\left(\theta \right)\geq \underset{\begin{array}{c} P_{X}:\\ E[b(X)]\leq \theta \end{array}}{\sup}\;\;\;\;\;\;I_{\alpha }\left(P_{X},P_{Y|X}\right).$$
  A conceptually simple approach to solve for $C_{\alpha }^{c}\left(\theta \right)$ is to
  (a) postulate an input probability measure $P_{X}^{*}$ that achieves the supremum in (197);
  (b) solve for its 〈α〉-response $P_{Y}^{*}$ using (92);
  (c) show that $\left(P_{X}^{*},P_{Y}^{*}\right)$ is a saddle point for the game with payoff function
    (199) $$\begin{array}{c} B\left(P_{X},Q_{Y}\right)=\int D_{\alpha }\left(P_{Y|X=x}∥Q_{Y}\right)\;dP_{X}, \end{array}$$
    where $Q_{Y}\in P_{A}$ and $P_{X}$ is chosen from the convex subset of $P_{A}$ of probability measures which satisfy E[b(X)]≤θ.
  Since $P_{Y}^{*}$ is already known, by definition, to be the 〈α〉-response to $P_{X}^{*}$, verifying the saddle point is tantamount to showing that $B(P_{X},P_{Y}^{*})$ is maximized by $P_{X}^{*}$ among $\left\{P_{X}\in P_{A}:E\left[b\left(X\right)\right]\leq \theta \right\}$. Theorem 1 of [43] guarantees the existence of a saddle point in the case of finite input alphabets. In addition to the fact that it is not always easy to guess the optimum input $P_{X}^{*}$ (see e.g., Section 12), the main stumbling block is the difficulty in determining the 〈α〉-response to any candidate input distribution, although sometimes this is indeed feasible as we saw in Example 7.
54. Naturally, Theorem 15 implies
  (200) $$\begin{array}{c} C_{\alpha }^{c}\left(\theta \right)\leq \underset{X}{\sup}I_{\alpha }\left(X;Y\right). \end{array}$$
  If the unconstrained maximization of $I_{\alpha }^{c}\left(\cdot ,P_{Y|X}\right)$ is achieved by an input distribution $X^{☆}$ that satisfies $E[b\left(X^{☆}\right)]\leq \theta$, then equality holds in (200), which, in turn, is equal to $I_{\alpha }^{c}\left(P_{X}^{☆},P_{Y|X}\right)$. In that case, the average cost constraint is said to be inactive. For most cost functions and random transformations of practical interest, the cost constraint is active for all θ>0. To ascertain whether it is, we simply verify whether there exists an input achieving the right side of (200), which happens to satisfy the constraint. If so, $C_{\alpha }^{c}\left(\theta \right)$ has been found. The same holds if we can find a sequence $\left\{X_{n}\right\}$ such that $E[b\left(X_{n}\right)]\leq \theta$ and $I_{\alpha }\left(X_{n};Y_{n}\right)\to \sup_{X}I_{\alpha }\left(X;Y\right)$. Otherwise, we proceed with the method described below. Thus, henceforth, we assume that the cost constraint is active.
55. The approach proposed in this paper to solve for $C_{\alpha }^{c}\left(\theta \right)$ for α∈(0,1) hinges on the variational representation in (172), which allows us to sidestep having to find any 〈α〉-response. Note that once we set out to maximize $I_{\alpha }^{c}\left(P_{X},P_{Y|X}\right)$ over $P=\{P_{X}\in P_{A}:E\left[b\left(X\right)\right]\leq \theta \}$, the allowable $Q_{X}$ in the maximization in (175) range over a ξ-blow-up of P defined by
  (201) $$\begin{array}{c} \Gamma_{\xi }\left(P\right)=\left\{Q_{X}\in P_{A}:\exists P_{X}\in P,\;suchthat\;D\left(P_{X}∥Q_{X}\right)\leq \xi \right\}. \end{array}$$
  As we show in Item 56, we can accomplish such an optimization by solving an unconstrained maximization of the sum of α-mutual information and a term suitably derived from the cost function.
56. It will not be necessary to solve for (176), as our goal is to further maximize (172) over $P_{X}$ subject to an average cost constraint. The Lagrangian corresponding to the constrained optimization in (197) is
  (202) $$\begin{array}{c} L_{\alpha }\left(\nu ,P_{X}\right)=I_{\alpha }^{c}\left(X;Y\right)-\nu \;E\left[b\left(X\right)\right]+\nu \;\theta , \end{array}$$
  where on the left side we have omitted, for brevity, the dependence on θ stemming from the last term on the right side. The Lagrange multiplier method (e.g., [60]) implies that if $X^{*}$ achieves the supremum in (197), then there exists $\nu^{*}\geq 0$ such that for all $P_{X}$ on A and ν≥0,
  (203) $$\begin{array}{c} L_{\alpha }\left(\nu^{*},P_{X}\right)\leq L_{\alpha }\left(\nu^{*},P_{X}^{*}\right)\leq L_{\alpha }\left(\nu ,P_{X}^{*}\right). \end{array}$$
  Note from (202) that the right inequality in (203) can only be achieved if
  (204) $$\begin{array}{c} E[b\left(X^{*}\right)]=\theta , \end{array}$$
  and, consequently,
  (205) $$\begin{array}{c} C_{\alpha }^{c}\left(\theta \right)=L_{\alpha }\left(\nu^{*},P_{X}^{*}\right)=\min_{\nu \geq 0}\max_{P_{X}}L_{\alpha }\left(\nu ,P_{X}\right)=\max_{P_{X}}\min_{\nu \geq 0}L_{\alpha }\left(\nu ,P_{X}\right). \end{array}$$
  The pivotal result enabling us to obtain $C_{\alpha }^{c}\left(\theta \right)$ without the need to deal with Augustin–Csiszár mutual information is the following.
  **Theorem** **16.** *Given α∈(0,1), ν≥0, $P_{Y|X}:A\to B$, and b:A→[0,∞), denote the function*
  (206) $$\begin{array}{c} A_{\alpha }\left(\nu \right)=\max_{X}\left\{I_{\alpha }\left(X;Y\right)+\frac{1}{1-\alpha }\log E\left[\exp\left(-(1-\alpha )\nu \;b(X)\right)\right]\right\}. \end{array}$$
*Then,*
  (207) $$\begin{array}{c} \underset{P_{X}\in P_{A}}{\sup}L_{\alpha }\left(\nu ,P_{X}\right)=\nu \;\theta +A_{\alpha }\left(\nu \right), \end{array}$$
*and*
  (208) $$\begin{array}{c} C_{\alpha }^{c}\left(\theta \right)=\min_{\nu \geq 0}\left\{\nu \;\theta +A_{\alpha }\left(\nu \right)\right\}. \end{array}$$
  **Proof.**  Plugging (172) into (197) we obtain, with $X∼P_{X}$, and $\hat{X}∼Q_{X}$,
  (209) $$\begin{array}{cc} \; & \underset{P_{X}\in P_{A}}{\sup}L_{\alpha }\left(\nu ,P_{X}\right)=\underset{P_{X}}{\sup}\left\{I_{\alpha }^{c}\left(X;Y\right)-\nu \;E\left[b\left(X\right)\right]+\nu \;\theta \right\} \end{array}$$
  (210) $$\begin{array}{cc} \; & =\underset{P_{X}\in P_{A}}{\sup}\left\{\max_{Q_{X}\in P_{A}}\left\{I_{\alpha }\left(Q_{X},P_{Y|X}\right)-\frac{1}{1-\alpha }D\left(P_{X}\;∥\;Q_{X}\right)\right\}-\nu \;E\left[b\left(X\right)\right]+\nu \;\theta \right\} \end{array}$$
  (211) $$\begin{array}{cc} \; & =\nu \;\theta +\max_{Q_{X}\in P_{A}}\left\{I_{\alpha }\left(Q_{X},P_{Y|X}\right)-\frac{1}{1-\alpha }\underset{P_{X}}{\inf}\left\{D\left(P_{X}\;∥\;Q_{X}\right)+\nu \left(1-\alpha \right)E\left[b\left(X\right)\right]\right\}\right\} \end{array}$$
  (212) $$\begin{array}{cc} \; & =\nu \;\theta +\max_{Q_{X}\in P_{A}}\left\{I_{\alpha }\left(Q_{X},P_{Y|X}\right)+\frac{1}{1-\alpha }\log E\left[\exp\left(-\nu \left(1-\alpha \right)b\left(\hat{X}\right)\right)\right]\right\} \end{array}$$
  (213) $$\begin{array}{cc} \; & =\nu \;\theta +A_{\alpha }\left(\nu \right), \end{array}$$
  where (209) and (213) follow from (202) and (206), respectively, and (212) follows by invoking Theorem 1 with $Z∼Q_{X}$ and
  (214) $$\begin{array}{c} g(a)=(1-\alpha )\nu \;b(a), \end{array}$$
  which is nonnegative since α∈(0,1) and ν>0. Finally, (208) follows from (205) and (207). □
  In conclusion, we have shown that the maximization of Augustin–Csiszár mutual information of order α subject to E[b(X)]≤θ boils down to the unconstrained maximization of a Lagrangian consisting of the sum of α-mutual information and an exponential average of the cost function. Circumventing the need to deal with 〈α〉-responses and with Augustin–Csiszár mutual information of order α leads to a particularly simple optimization, as illustrated in Section 11 and Section 12.
57. Theorem 16 solves for the maximal Augustin–Csiszár mutual information of order α under an average cost constraint without having to find out the input probability measure $P_{X}^{*}$ that attains it nor its 〈α〉-response $P_{Y}^{*}$ (using the notation in Item 53). Instead, it gives the solution as
  (215) $$\begin{array}{c} C_{\alpha }^{c}\left(\theta \right)=\min_{\nu \geq 0}\left\{\nu \;\theta +\max_{X}\left\{I_{\alpha }\left(X;Y\right)+\frac{1}{1-\alpha }\log E\left[\exp\left(-(1-\alpha )\nu \;b(X)\right)\right]\right\}\right\}. \end{array}$$
  Although we are not going to invoke a minimax theorem, with the aid of Theorem 9-(b) we can see that the functional within the inner brackets is concave in $P_{X}$; Furthermore, if V∈(0,1], then $\log E\left[V^{\nu }\right]$ is easily seen to be convex in ν with the aid of the Cauchy-Schwarz inequality. Before we characterize the saddle point $\left(\nu^{*},Q_{X}^{*}\right)$ of the game in (215) we note that $\left(P_{X}^{*},P_{Y}^{*}\right)$ can be readily obtained from $\left(\nu^{*},Q_{X}^{*}\right)$.
  **Theorem** **17.** Fix α∈(0,1). Let $\nu^{*}>0$ denote the minimizer on the right side of (215), and $Q_{X}^{*}$ the input probability measure that attains the maximum in (206) (or (215)) for $\nu =\nu^{*}$. Then,
  (*a*) $Q_{X}^{*}$ is the 〈α〉-adjunct of $P_{X}^{*}$.
  (*b*) $P_{Y}^{*}=Q_{Y[\alpha ]}^{*}$, the α-response to $Q_{X}^{*}$.
  (*c*) $P_{X}^{*}\ll \gg Q_{X}^{*}$ with
    (216) $$\begin{array}{c} {ı}_{P_{X}^{*}∥Q_{X}^{*}}\left(a\right)=-\left(1-\alpha \right)\nu^{*}\;b\left(a\right)+\tau_{\alpha },\;a\in A, \end{array}$$
    where $\tau_{\alpha }$ is a normalizing constant ensuring that $P_{X}^{*}$ is a probability measure.
  **Proof.** 
  (a) We had already established in Theorem 13 that the maximum on the right side of (210) is achieved by the 〈α〉-adjunct of $P_{X}$. In the special case $\nu =\nu^{*}$, such $P_{X}$ is $P_{X}^{*}$. Therefore, $Q_{X}^{*}$, the argument that achieves the maximum in (206) for $\nu =\nu^{*}$, is the 〈α〉-adjunct of $P_{X}^{*}$.
  (b) According to Theorem 11, the α-response to $Q_{X}^{*}$ is the 〈α〉-response to $P_{X}^{*}$, which is $P_{Y}^{*}$ by definition.
  (c) For $\nu =\nu^{*}$, $P_{X}^{*}$ achieves the supremum in (209) and the infimum in (211). Therefore, (216) follows from Theorem 1 with $Z∼Q_{X}^{*}$ and g(·) given by (214) particularized to $\nu =\nu^{*}$.
  □
  The saddle point of (215) admits the following characterization.
  **Theorem** **18.** If α∈(0,1), the saddle point $\left(\nu^{*},Q_{X}^{*}\right)$ of (215) satisfies
  (217) $$\begin{array}{cc} E\left[b\left({\bar{X}}^{*}\right)\exp\left(-\left(1-\alpha \right)\nu^{*}\;b\left({\bar{X}}^{*}\right)\right)\right]\; & =\theta \;E\left[\exp\left(-\left(1-\alpha \right)\nu^{*}\;b\left({\bar{X}}^{*}\right)\right)\right],\;{\bar{X}}^{*}∼Q_{X}^{*}; \end{array}$$
  (218) $$\begin{array}{cc} D_{\alpha }\left(P_{Y|X=a}\;∥\;Q_{Y[\alpha ]}^{*}\right) & =\nu^{*}\;b\left(a\right)+c_{\alpha }\left(\nu^{*}\right),\;a\in A, \end{array}$$
  where $Q_{Y[\alpha ]}^{*}$ is the α-response to $Q_{X}^{*}$, and $c_{\alpha }\left(\nu^{*}\right)$ does not depend on a∈A. Furthermore,
  (219) $$\begin{array}{cc} A_{\alpha }\left(\nu^{*}\right) & =c_{\alpha }\left(\nu^{*}\right), \end{array}$$
  (220) $$\begin{array}{cc} C_{\alpha }^{c}\left(\theta \right) & =\nu^{*}\;\theta +c_{\alpha }\left(\nu^{*}\right). \end{array}$$
  **Proof.** First, we show that the scalar $\nu^{*}\geq 0$ that minimizes
  (221) $$\begin{array}{c} f\left(\nu \right)=\nu \;\theta +I_{\alpha }\left(Q_{X}^{*},P_{Y|X}\right)+\frac{1}{1-\alpha }\log E\left[\exp\left(-\left(1-\alpha \right)\nu \;b\left({\bar{X}}^{*}\right)\right)\right] \end{array}$$
  satisfies (217). If we abbreviate $V=\exp\left(-\left(1-\alpha \right)b\left({\bar{X}}^{*}\right)\right)\in \left(0,1\right]$, then the dominated convergence theorem results in
  (222) $$\begin{array}{c} \frac{d}{d\nu }\left\{\nu \;\theta +\frac{1}{1-\alpha }\log E\left[V^{\nu }\right]\right\}=\theta +\frac{1}{1-\alpha }\frac{E\left[V^{\nu }\log V\right]}{E\left[V^{\nu }\right]}. \end{array}$$
  Therefore, (217) is equivalent to $\dot{f}\left(\nu^{*}\right)=0$, which is all we need on account of the convexity of f(·). To show (218), notice that for all a∈A,
  (223) $$\begin{array}{cc} \left(1-\alpha \right)\nu^{*}\;b\left(a\right)-\tau_{\alpha } & ={ı}_{Q_{X}^{*}∥P_{X}^{*}}\left(a\right) \end{array}$$
  (224) $$\begin{array}{cc} \; & =\left(1-\alpha \right)D_{\alpha }\left(P_{Y|X=a}\;∥\;P_{Y}^{*}\right)+\upsilon_{\alpha }, \end{array}$$
  where (223) is (216) and (224) is (157) with $P_{Y\langle \alpha \rangle }\leftarrow P_{Y}^{*}$ in view of Theorem 17-(b). In conclusion, (218) holds with
  (225) $$\begin{array}{c} c_{\alpha }\left(\nu^{*}\right)=\frac{\upsilon_{\alpha }+\tau_{\alpha }}{\alpha -1}. \end{array}$$
  Finally, (206) implies
  (226) $$\begin{array}{cc} A_{\alpha }\left(\nu^{*}\right)\; & =I_{\alpha }\left(Q_{X}^{*},P_{Y|X}\right)+\frac{1}{1-\alpha }\log E\left[\exp\left(-\left(1-\alpha \right)\nu^{*}\;b\left({\bar{X}}^{*}\right)\right)\right]\\ \; & =\frac{1}{\alpha -1}\log E\left[\exp\left(\left(\alpha -1\right)D_{\alpha }\left(P_{Y|X}\left(\cdot |{\bar{X}}^{*}\right)\;∥\;P_{Y}^{*}\right)\right)\right] \end{array}$$
  (227) $$\begin{array}{cc} \; & \;+\frac{1}{1-\alpha }\log E\left[\exp\left(\left(\alpha -1\right)\nu^{*}\;b\left({\bar{X}}^{*}\right)\right)\right]\\ \; & =\frac{1}{\alpha -1}\log E\left[\exp\left(\left(\alpha -1\right)\left(\nu^{*}\;b\left({\bar{X}}^{*}\right)+c_{\alpha }\left(\nu^{*}\right)\right)\right)\right] \end{array}$$
  (228) $$\begin{array}{cc} \; & \;+\frac{1}{1-\alpha }\log E\left[\exp\left(\left(\alpha -1\right)\nu^{*}\;b\left({\bar{X}}^{*}\right)\right)\right] \end{array}$$
  (229) $$\begin{array}{cc} \; & =c_{\alpha }\left(\nu^{*}\right), \end{array}$$
  where (227) follows from the definition of α-mutual information and Theorem 17-(b), and (228) follows from (218). Plugging (219) into (208) results in (220). □
58. Typically, the application of Theorem 18 involves
  (a) guessing the form of the auxiliary input $Q_{X}^{*}$ (modulo some unknown parameter),
  (b) obtaining its α-response $Q_{Y[\alpha ]}^{*}$, and
  (c) verifying that (217) and (218) are satisfied for some specific choice of the unknown parameter.
  With the same approach, we can postulate, for every ν≥0, an input distribution $R_{X}^{\nu }$, whose α-response $R_{Y[\alpha ]}^{\nu }$ satisfies
  (230) $$\begin{array}{c} D_{\alpha }\left(P_{Y|X=a}\;∥\;R_{Y[\alpha ]}^{\nu }\right)=\nu \;b\left(a\right)+c_{\alpha }\left(\nu \right),\;a\in A, \end{array}$$
  where the only condition we place on $c_{\alpha }\left(\nu \right)$ is that it not depend on a∈A. If this is indeed the case, then the same derivation in (226)–(229) results in
  (231) $$\begin{array}{c} A_{\alpha }\left(\nu \right)=c_{\alpha }\left(\nu \right), \end{array}$$
  and we determine $\nu^{*}$ as the solution to $\theta =-{\dot{c}}_{\alpha }\left(\nu^{*}\right)$, in lieu of (217). Section 11 and Section 12 illustrate the effortless nature of this approach to solve for $A_{\alpha }\left(\nu \right)$. Incidentally, (230) can be seen as the α-generalization of the condition in Problem 8.2 of [48], elaborated later in [61].

## 9. Gallager’s $E_{0}$ Functions and the Maximal Augustin–Csiszár Mutual Information

In keeping with Gallager’s setting [9], we stick to discrete alphabets throughout this section.

59. In his derivation of an achievability result for discrete memoryless channels, Gallager [8] introduced the function (1), which we repeat for convenience,
  (232) $$\begin{array}{c} E_{0}\left(\rho ,P_{X}\right)=-\log\sum_{y\in B}{\left(\sum_{x\in A}P_{X}\left(x\right)P_{Y|X}^{\frac{1}{1+\rho }}\left(y|x\right)\right)}^{1+\rho }\;. \end{array}$$
  Comparing (82) and (232), we obtain
  (233) $$\begin{array}{c} E_{0}\left(\rho ,P_{X}\right)=\rho \;I_{\frac{1}{1+\rho }}\left(X;Y\right), \end{array}$$
  which, as we mentioned in Section 1, is the observation by Csiszár in [30] that triggered the third phase in the representation of error exponents. Popularized in [9], the $E_{0}$ function was employed by Shannon, Gallager and Berlekamp [10] for ρ≥0 and by Arimoto [62] for ρ∈(−1,0) in the derivation of converse results in data transmission, the latter of which considers rates above capacity, a region in which error probability increases with blocklength, approaching one at an exponential rate. For the achievability part, [8] showed upper bounds on the error probability involving $E_{0}\left(\rho ,P_{X}\right)$ for ρ∈[0,1]. Therefore, for rates below capacity, the α-mutual information only enters the picture for α∈(0,1). One exception in which Rényi divergence of order greater than 1 plays a role at rates below capacity was found by Sason [63], where a refined achievability result is shown for binary linear codes for output symmetric channels (a case in which equiprobable $P_{X}$ maximizes (233)), as a function of their Hamming weight distribution.
  Although Gallager did not have the benefit of the insight provided by the Rényi information measures, he did notice certain behaviors of $E_{0}$ reminiscent of mutual information. For example, the derivative of (233) with respect to ρ, at ρ←0 is equal to I(X;Y). As pointed out by Csiszár in [32], in the absence of cost constraints, Gallager’s $E_{0}$ function in (232) satisfies
  (234) $$\begin{array}{c} \max_{P_{X}}E_{0}\left(\rho ,P_{X}\right)=\rho \;\max_{X}I_{\frac{1}{1+\rho }}\left(X;Y\right)=\rho \;\max_{X}I_{\frac{1}{1+\rho }}^{c}\left(X;Y\right), \end{array}$$
  in view of (233) and (187).
  Recall that Gallager’s modified $E_{0}$ function in the case of cost constraints is
  (235) $$\begin{array}{c} E_{0}\left(\rho ,P_{X},r,\theta \right)=-\log\sum_{y\in B}{\left(\sum_{x\in A}P_{X}\left(x\right)\exp\left(r\;b(x)-r\;\theta \right)P_{Y|X}^{\frac{1}{1+\rho }}\left(y|x\right)\right)}^{1+\rho }\;, \end{array}$$
  which, like (232) he introduced in order to show an achievability result. Up until now, no counterpart to (234) has been found with cost constraints and (235). This is accomplished in the remainder of this section.
60. In the finite alphabet case the following result is useful to obtain a numerical solution for the functional in (206). More importantly, it is relevant to the discussion in Item 61.
  **Theorem** **19.** *In the special case of discrete alphabets, the function in (206) is equal to*
  (236) $$\begin{array}{c} A_{\alpha }\left(\nu \right)=\max_{G}\frac{\alpha }{\alpha -1}\log\sum_{y\in B}{\left(\sum_{a\in A}G\left(a\right)\;P_{Y|X}^{\alpha }\left(y|a\right)\right)}^{\frac{1}{\alpha }}\;, \end{array}$$
  *where the maximization is over all G:A→[0,∞) such that*
  (237) $$\begin{array}{c} \sum_{a\in A}G\left(a\right)\exp\left(-(1-\alpha )\nu b(a)\right)=1. \end{array}$$
  **Proof.**  Recalling (82) we have
  $$\begin{array}{cc} \; & \;I_{\alpha }\left(X;Y\right)+\frac{1}{1-\alpha }\log E\left[\exp\left(-(1-\alpha )\nu \;b(X)\right)\right]\\ \; & =\frac{\alpha }{\alpha -1}\log\sum_{y\in B}{\left(\sum_{x\in A}P_{X}\left(x\right)P_{Y|X=x}^{\alpha }\left(y\right)\right)}^{\frac{1}{\alpha }} \end{array}$$
  (238) $$\begin{array}{cc} \; & \;+\frac{1}{1-\alpha }\log E\left[\exp\left(-(1-\alpha )\nu \;b(X)\right)\right] \end{array}$$
  (239) $$\begin{array}{cc} \; & =\frac{\alpha }{\alpha -1}\log\sum_{y\in B}{\left(\frac{E\left[P_{Y|X}^{\alpha }\left(y|X\right)\right]}{E\left[\exp\left(-(1-\alpha )\nu b(X)\right)\right]}\right)}^{\frac{1}{\alpha }} \end{array}$$
  (240) $$\begin{array}{cc} \; & =\frac{\alpha }{\alpha -1}\log\sum_{y\in B}{\left(\sum_{a\in A}G\left(a\right)\;P_{Y|X}^{\alpha }\left(y|a\right)\right)}^{\frac{1}{\alpha }}\;, \end{array}$$
  where
  (241) $$\begin{array}{c} G\left(x\right)=\frac{P_{X}\left(x\right)}{\sum_{a\in A}P_{X}\left(a\right)\exp\left(-(1-\alpha )\nu \;b(a)\right)}. \end{array}$$
  □
61. We can now proceed to close the circle between the maximization of Augustin–Csiszár mutual information subject to average cost constraints (Phase 3 in Section 1) and Gallager’s approach (Phase 1 in Section 1).
  **Theorem** **20.** *In the discrete alphabet case, recalling the definitions in (202) and (235), for ρ>0,*
  (242) $$\begin{array}{cc} \max_{P_{X}}E_{0}\left(\rho ,P_{X},r,\theta \right) & =\rho \max_{P_{X}}\;L_{\frac{1}{1+\rho }}\left(r+\frac{r}{\rho },P_{X}\right),\;r>0; \end{array}$$
  (243) $$\begin{array}{cc} \min_{r\geq 0}\max_{P_{X}}E_{0}\left(\rho ,P_{X},r,\theta \right) & =\rho \;C_{\frac{1}{1+\rho }}^{c}\left(\theta \right), \end{array}$$
  *where the maximizations are over $P_{A}$.*
  **Proof.**  With
  (244) $$\begin{array}{c} \alpha =\frac{1}{1+\rho }\;and\;\nu =r\frac{1+\rho }{\rho }=\frac{r}{1-\alpha }, \end{array}$$
  the maximization of (235) with the respect to the input probability measure yields
  $$\begin{array}{cc} \; & \;\max_{P_{X}}E_{0}\left(\rho ,P_{X},r,\theta \right) \end{array}$$
  (245) $$\begin{array}{cc} \; & =\max_{P_{X}}\left\{\left(1+\rho \right)\;r\;\theta -\log\sum_{y\in B}{\left(\sum_{x\in A}P_{X}\left(x\right)\exp\left(r\;b(x)\right)P_{Y|X}^{\frac{1}{1+\rho }}\left(y|x\right)\right)}^{1+\rho }\right\} \end{array}$$
  (246) $$\begin{array}{cc} \; & =\rho \;\nu \;\theta +\rho \max_{P_{X}}\frac{\alpha }{\alpha -1}\log\sum_{y\in B}{\left(\sum_{x\in A}P_{X}\left(x\right)\exp\left((1-\alpha )\;\nu \;b(x)\right)P_{Y|X}^{\alpha }\left(y|x\right)\right)}^{\frac{1}{\alpha }} \end{array}$$
  (247) $$\begin{array}{cc} \; & =\rho \;\nu \;\theta +\rho \max_{G}\frac{\alpha }{\alpha -1}\log\sum_{y\in B}{\left(\sum_{x\in A}G\left(x\right)P_{Y|X}^{\alpha }\left(y|x\right)\right)}^{\frac{1}{\alpha }} \end{array}$$
  (248) $$\begin{array}{cc} \; & =\rho \;\nu \;\theta +\rho \;A_{\alpha }\left(\nu \right) \end{array}$$
  (249) $$\begin{array}{cc} \; & =\rho \max_{P_{X}}L_{\alpha }\left(\nu ,P_{X}\right), \end{array}$$
  where
  - the maximization on the right side of (247) is over all G:A→[0,∞) that satisfy (237), since that constraint is tantamount to enforcing the constraint that $P_{X}\in P_{A}$ on the left side of (247);
  - (248) ⟸ Theorem 19;
  - (249) ⟸ Theorem 16.
  The proof of (242) is complete once (244) is invoked to substitute α and ν from the right side of (249). If we now minimize the outer sides of (245)–(249) with respect to *r* we obtain, using (205) and (244),
  (250) $$\begin{array}{cc} \min_{r\geq 0}\max_{P_{X}}E_{0}\left(\rho ,P_{X},r,\theta \right) & =\rho \min_{r\geq 0}\max_{P_{X}}L_{\alpha }\left(\frac{r}{1-\alpha },P_{X}\right) \end{array}$$
  (251) $$\begin{array}{cc} \; & =\rho \min_{\nu \geq 0}\max_{P_{X}}L_{\alpha }\left(\nu ,P_{X}\right) \end{array}$$
  (252) $$\begin{array}{cc} \; & =\rho \;C_{\frac{1}{1+\rho }}^{c}\left(\theta \right). \end{array}$$
  □
  In p. 329 of [9], Gallager poses the unconstrained maximization (i.e., over $P_{X}\in P_{A}$) of the Lagrangian
  (253) $$\begin{array}{c} E_{0}\left(\rho ,P_{X},r,\theta \right)+\gamma \sum_{a\in A}P_{X}\left(a\right)b\left(a\right)-\gamma \;\theta . \end{array}$$
  Note the apparent discrepancy between the optimizations in (243) and (253): the latter is parametrized by *r* and γ (in addition to ρ and θ), while the maximization on the right side of (243) does not enforce any average cost constraint. In fact, there is no disparity since Gallager loc. cit. finds serendipitously that γ=0 regardless of *r* and θ, and, therefore, just one parameter is enough.
62. The raison d’être for Augustin’s introduction of $I_{\alpha }^{c}$ in [36] was his quest to view Gallager’s approach with average cost constraints under the optic of Rényi information measures. Contrasting (232) and (235) and inspired by the fact that, in the absence of cost constraints, (232) satisfies a variational characterization in view of (69) and (233), Augustin [36] dealt, not with (235), but with
  $$\min_{Q_{Y}}D_{\alpha }({\tilde{P}}_{Y|X}∥Q_{Y}\left|P_{X}\right),\;where\;\;{\tilde{P}}_{Y|X=x}=P_{Y|X=x}\exp\left(r^{\prime }b\left(x\right)\right).$$
  Assuming finite alphabets, Augustin was able to connect this quantity with the maximal $I_{\alpha }^{c}\left(X;Y\right)$ under cost constraints in an arcane analysis that invokes a minimax theorem. This line of work was continued in Section 5 of [43], which refers to $\min_{Q_{Y}}D_{\alpha }({\tilde{P}}_{Y|X}∥Q_{Y}\left|P_{X}\right)$ as the Rényi-Gallager information. Unfortunately, since ${\tilde{P}}_{Y|X}$ is not a random transformation, the conditional pseudo-Rényi divergence $D_{\alpha }({\tilde{P}}_{Y|X}∥Q_{Y}\left|P_{X}\right)$ need not satisfy the key additive decomposition in Theorem 4 so the approach of [36,43] fails to establish an identity equating the maximization of Gallager’s function (235) with the maximization of Augustin–Csiszár mutual information, which is what we have accomplished through a crisp and elementary analysis.

## 10. Error Exponent Functions

The central objects of interest in the error exponent analysis of data transmission are the functions $E_{\mathrm{sp}}\left(R,P_{X}\right)$ and $E_{r}\left(R,P_{X}\right)$ of a random transformation $P_{Y|X}:A\to B$. Reflecting the three different phases referred to in Section 1, there is no unanimity in the definition of those functions. Following [48], we adopt the standard canonical Phase 2 (Section 1.2) definitions of those functions, which are given in Items 63 and 67.

**Figure 1:**
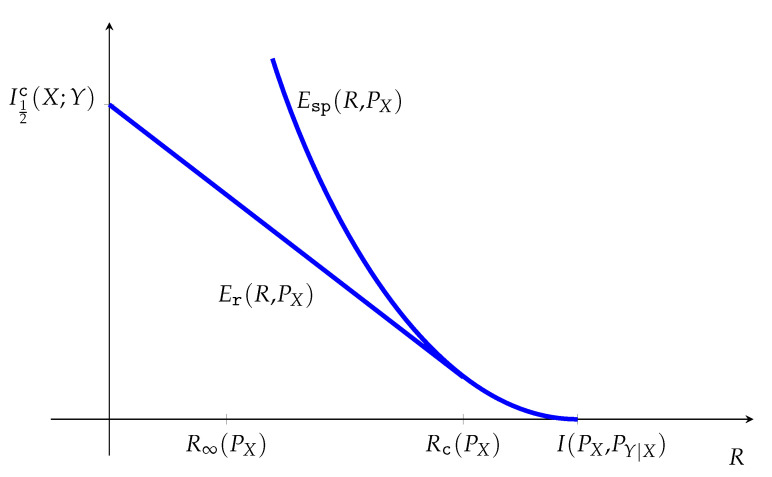
$E_{\mathrm{sp}}\left(\cdot ,P_{X}\right)$ and $E_{r}\left(\cdot ,P_{X}\right)$.

63. If R≥0 and $P_{X}\in P_{A}$, the sphere-packing error exponent function is (e.g., (10.19) of [48])
  (254) $$E_{\mathrm{sp}}\left(R,P_{X}\right)=\min_{\begin{array}{c} Q_{Y|X}:A\to B\\ I(P_{X},Q_{Y|X})\leq R\;\; \end{array}}D(Q_{Y|X}\;∥\;P_{Y|X}\;\left|\;P_{X}\right).$$
64. As a function of R≥0, the basic properties of (254) for fixed $\left(P_{X},P_{Y|X}\right)$ are as follows.
  (a) If $R\geq I(P_{X},P_{Y|X})$, then $E_{\mathrm{sp}}\left(R,P_{X}\right)=0$;
  (b) If $R<I(P_{X},P_{Y|X})$, then $E_{\mathrm{sp}}\left(R,P_{X}\right)>0$;
  (c) The infimum of the arguments for which the sphere-packing error exponent function is finite is denoted by $R_{\infty }\left(P_{X}\right)$;
  (d) On the interval $R\in (R_{\infty }\left(P_{X}\right),I\left(P_{X},P_{Y|X}\right))$, $E_{\mathrm{sp}}\left(R,P_{X}\right)$ is convex, strictly decreasing, continuous, and equal to (254) where the constraint is satisfied with equality. This implies that for *R* belonging to that interval, we can find $\rho_{R}\geq 0$ so that for all r≥0,
    (255) $$\begin{array}{c} E_{\mathrm{sp}}\left(r,P_{X}\right)\geq E_{\mathrm{sp}}\left(R,P_{X}\right)-\rho_{R}\;r+\rho_{R}\;R. \end{array}$$
65. In view of Theorem 8 and its definition in (254), it is not surprising that $E_{\mathrm{sp}}\left(R,P_{X}\right)$ is intimately related to the Augustin–Csiszár mutual information, through the following key identity.
  **Theorem** **21.** (256) $$\begin{array}{cc} E_{\mathrm{sp}}\left(R,P_{X}\right) & =\underset{\rho \geq 0}{\sup}\left\{\rho \;I_{\frac{1}{1+\rho }}^{c}\left(X;Y\right)-\rho \;R\right\},\;R\geq 0; \end{array}$$
  (257) $$\begin{array}{cc} R_{\infty }\left(P_{X}\right) & =I_{0}^{c}\left(X;Y\right). \end{array}$$
  **Proof.**  First note that ≥ holds in (256) because from (128) we obtain, for all ρ≥0,
  (258) $$\begin{array}{cc} \; & \;\rho \;I_{\frac{1}{1+\rho }}^{c}\left(X;Y\right)=\min_{Q_{Y|X}}\left\{D(Q_{Y|X}∥P_{Y|X}\left|P_{X}\right)+\rho \;I\left(P_{X},Q_{Y|X}\right)\right\} \end{array}$$
  (259) $$\leq \min_{\begin{array}{c} Q_{Y|X}:\\ I(P_{X},Q_{Y|X})\leq R \end{array}}\left\{D(Q_{Y|X}∥P_{Y|X}\left|P_{X}\right)+\rho \;I\left(P_{X},Q_{Y|X}\right)\right\}$$
  (260) $$\begin{array}{cc} \; & \leq E_{\mathrm{sp}}\left(R,P_{X}\right)+\rho \;R, \end{array}$$
  where (260) follows from the definition in (254). To show ≤ in (256) for those *R* such that $0<E_{\mathrm{sp}}\left(R,P_{X}\right)<\infty$, Property (d) in Item 64 allows us to write
  (261) $$\begin{array}{cc} \min_{Q_{Y|X}}\left\{D(Q_{Y|X}∥P_{Y|X}\left|P_{X}\right)+\rho_{R}\;I\left(P_{X},Q_{Y|X}\right)\right\}\; & =\min_{r\geq 0}\left\{E_{\mathrm{sp}}\left(r,P_{X}\right)+\rho_{R}\;r\right\} \end{array}$$
  (262) $$\begin{array}{cc} \; & \geq E_{\mathrm{sp}}\left(R,P_{X}\right)+\rho_{R}\;R, \end{array}$$
  where (262) follows from (255).
  To determine the region where the sphere-packing error exponent is infinite and show (257), first note that if $R<I_{0}^{c}\left(X;Y\right)=\lim_{\alpha ↓0}I_{\alpha }^{c}\left(X;Y\right)$, then $E_{\mathrm{sp}}\left(R,P_{X}\right)=\infty$ because for any ρ≥0, the function in {} on the right side of (256) satisfies
  (263) $$\begin{array}{cc} \rho \;I_{\frac{1}{1+\rho }}^{c}\left(X;Y\right)-\rho \;R\; & =\rho \;I_{\frac{1}{1+\rho }}^{c}\left(X;Y\right)-\rho \;I_{0}^{c}\left(X;Y\right)+\rho \;I_{0}^{c}\left(X;Y\right)-\rho \;R \end{array}$$
  (264) $$\begin{array}{cc} \; & \geq \rho \;I_{0}^{c}\left(X;Y\right)-\rho \;R, \end{array}$$
  where (264) follows from the monotonicity of $I_{\alpha }^{c}\left(X;Y\right)$ in α we saw in (143). Conversely, if $I_{0}^{c}\left(X;Y\right)<R<\infty$, there exists ϵ∈(0,1) such that $I_{\epsilon }^{c}\left(X;Y\right)<R$, which implies that in the minimization
  (265) $$\begin{array}{c} I_{\epsilon }^{c}\left(X;Y\right)=\min_{Q_{Y|X}}\left\{\frac{\epsilon }{1-\epsilon }D(Q_{Y|X}∥P_{Y|X}\left|P_{X}\right)+I\left(P_{X},Q_{Y|X}\right)\right\} \end{array}$$
  we may restrict to those $Q_{Y|X}$ such that $I(P_{X},Q_{Y|X})\leq R$, and consequently, $I_{\epsilon }^{c}\left(X;Y\right)\geq \frac{\epsilon }{1-\epsilon }E_{\mathrm{sp}}\left(R,P_{X}\right)$. Therefore, to avoid a contradiction, we must have $E_{\mathrm{sp}}\left(R,P_{X}\right)<\infty$.
  The remaining case is $I_{0}^{c}\left(X;Y\right)=\infty$. Again, the monotonicity of the Augustin–Csiszár mutual information implies that $I_{\alpha }^{c}\left(X;Y\right)=\infty$ for all α>0. So, (128) prescribes $D(Q_{Y|X}∥P_{Y|X}|P_{X})=\infty$ for any $Q_{Y|X}$ is such that $I(P_{X},Q_{Y|X})<\infty$. Therefore, $E_{\mathrm{sp}}\left(R,P_{X}\right)=\infty$ for all R≥0, as we wanted to show. □
  Augustin [36] provided lower bounds on error probability for codes of type $P_{X}$ as a function of $I_{\alpha }^{c}\left(X;Y\right)$ but did not state (256); neither did Csiszár in [32] as he was interested in a non-conventional parametrization (generalized cutoff rates) of the reliability function. As pointed out in p. 5605 of [64], the ingredients for the proof of (256) were already present in the hint of Problem 23 of Section II.5 of [24]. In the discrete case, an exponential lower bound on error probability for codes with constant composition $P_{X}$ is given as a function of $I_{\frac{1}{1+\rho }}^{c}\left(P_{X},P_{Y|X}\right)$ in [44,64]. As in [64], Nakiboglu [65] gives (256) as the definition of the sphere-packing function and connects it with (254) in Lemma 3 therein, within the context of discrete input alphabets.
  In the discrete case, (257) is well-known (e.g., [66]), and given by (83). As pointed out in [40], $\max_{X}I_{0}^{c}\left(X;Y\right)$ is the zero-error capacity with noiseless feedback found by Shannon [67], provided there is at least a pair $\left(a_{1},a_{2}\right)\in A^{2}$ such that $P_{Y|X=a_{1}}⊥P_{Y|X=a_{2}}$. Otherwise, the zero-error capacity with feedback is zero.
66. The critical rate, $R_{c}\left(P_{X}\right)$, is defined as the smallest abscissa at which the convex function $E_{\mathrm{sp}}\left(\cdot ,P_{X}\right)$ meets its supporting line of slope −1. According to (256),
  (266) $$\begin{array}{c} I_{\frac{1}{2}}^{c}\left(X;Y\right)=R_{c}\left(P_{X}\right)+E_{\mathrm{sp}}\left(R_{c}\left(P_{X}\right),P_{X}\right). \end{array}$$
67. If R≥0 and $P_{X}\in P_{A}$, the random-coding exponent function is (e.g., (10.15) of [48])
  (267) $$\begin{array}{c} E_{r}\left(R,P_{X}\right)=\min_{Q_{Y|X}:A\to B}\left\{D(Q_{Y|X}∥P_{Y|X}\left|P_{X}\right)+{\left[I\left(P_{X},Q_{Y|X}\right)-R\right]}^{+}\right\}, \end{array}$$
  with ${\left[t\right]}^{+}=\max\left\{0,t\right\}$.
68. The random-coding error exponent function is determined by the sphere-packing error exponent function through the following relation, illustrated in Figure 1.
  **Theorem** **22.** (268) $$\begin{array}{cc} E_{r}\left(R,P_{X}\right) & =\min_{r\geq R}\left\{E_{\mathrm{sp}}\left(r,P_{X}\right)+r-R\right\} \end{array}$$
  (269) $$=\left\{\begin{array}{cc} 0, & R\geq I(P_{X},P_{Y|X});\\ E_{\mathrm{sp}}\left(R,P_{X}\right), & R\in [R_{c}\left(P_{X}\right),I\left(P_{X},P_{Y|X}\right)];\\ I_{\frac{1}{2}}^{c}\left(X;Y\right)-R, & R\in [0,R_{c}\left(P_{X}\right)]. \end{array}\right.$$
  (270) $$\begin{array}{cc} \; & =\underset{\rho \in [0,1]}{\sup}\left\{\rho \;I_{\frac{1}{1+\rho }}^{c}\left(X;Y\right)-\rho \;R\right\}. \end{array}$$
  **Proof.**  Identities (268) and (269) are well-known (e.g., Lemma 10.4 and Corollary 10.4 in [48]). To show (270), note that (256) expresses $E_{\mathrm{sp}}\left(\cdot ,P_{X}\right)$ as the supremum of supporting lines parametrized by their slope −ρ. By definition of critical rate (for brevity, we do not show explicitly its dependence on $P_{X}$), if $R\in [R_{c},I\left(P_{X},P_{Y|X}\right)]$, then $E_{\mathrm{sp}}\left(R,P_{X}\right)$ can be obtained by restricting the optimization in (256) to ρ∈[0,1]. In that segment of values of *R*, $E_{\mathrm{sp}}\left(R,P_{X}\right)=E_{r}\left(R,P_{X}\right)$ according to (269). Moreover, on the interval $R\in [0,R_{c}]$, we have
  (271) $$\begin{array}{cc} \max_{\rho \in [0,1]}\left\{\rho \;I_{\frac{1}{1+\rho }}^{c}\left(X;Y\right)-\rho \;R\right\}\; & =I_{\frac{1}{2}}^{c}\left(X;Y\right)-R \end{array}$$
  (272) $$\begin{array}{cc} \; & =E_{\mathrm{sp}}\left(R_{c},P_{X}\right)+R_{c}-R \end{array}$$
  (273) $$\begin{array}{cc} \; & =E_{r}\left(R,P_{X}\right), \end{array}$$
  where we have used (266) and (269). □
  The first explicit connection between $E_{r}\left(R,P_{X}\right)$ and the Augustin–Csiszár mutual information was made by Poltyrev [35] although he used a different form for $I_{\alpha }^{c}\left(X;Y\right)$, as we discussed in (29).
69. The unconstrained maximizations over the input distribution of the sphere-packing and random coding error exponent functions are denoted, respectively, by
  (274) $$\begin{array}{cc} E_{\mathrm{sp}}\left(R\right) & =\underset{P_{X}}{\sup}E_{\mathrm{sp}}\left(R,P_{X}\right), \end{array}$$
  (275) $$\begin{array}{cc} E_{r}\left(R\right) & =\underset{P_{X}}{\sup}E_{r}\left(R,P_{X}\right). \end{array}$$
  Coding theorems [8,9,10,22,48] have shown that when these functions coincide they yield the reliability function (optimum speed at which the error probability vanishes with blocklength) as a function of the rate $R<\max_{X}I\left(X;Y\right)$. The intuition is that, for the most favorable input distribution, errors occur when the channel behaves so atypically that codes of rate *R* are not reliable. There are many ways in which the channel may exhibit such behavior and they are all unlikely, but the most likely among them is the one that achieves (254).
  It follows from (187), (256) and (270) that (274) and (275) can be expressed as
  (276) $$\begin{array}{cc} E_{\mathrm{sp}}\left(R\right) & =\underset{\rho \geq 0}{\sup}\left\{\rho \;\underset{X}{\sup}I_{\frac{1}{1+\rho }}\left(X;Y\right)-\rho \;R\right\}, \end{array}$$
  (277) $$\begin{array}{cc} E_{r}\left(R\right) & =\underset{\rho \in [0,1]}{\sup}\left\{\rho \;\underset{X}{\sup}I_{\frac{1}{1+\rho }}\left(X;Y\right)-\rho \;R\right\}. \end{array}$$
  Therefore, we can sidestep working with the Augustin–Csiszár mutual information in the absence of cost constraints.
70. Shannon [1] showed that, operating at rates below maximal mutual information, it is possible to find codes whose error probability vanishes with blocklength; for the converse, instead of error probability, Shannon measured reliability by the conditional entropy of the message given the channel output. That alternative reliability measure, as well as its generalization to Arimoto-Rényi conditional entropy, is also useful analyzing the average performance over code ensembles. It turns out (see e.g., [28,68]) that, below capacity, those conditional entropies also vanish exponentially fast in much the same way as error probability with bounds that are governed by $E_{\mathrm{sp}}\left(R\right)$ and $E_{r}\left(R\right)$ thereby lending additional operational significance to those functions.
71. We now introduce a cost function b:A→[0,∞) and real scalar θ≥0, and reexamine the optimizations in (274) and (275) allowing only those probability measures that satisfy E[b(X)]≤θ. With a patent, but unavoidable, abuse of notation we define
  (278) $$\begin{array}{cc} \; & E_{\mathrm{sp}}\left(R,\theta \right)=\underset{\begin{array}{c} P_{X}:\\ E[b(X)]\leq \theta \end{array}}{\sup}E_{\mathrm{sp}}\left(R,P_{X}\right) \end{array}$$
  (279) $$\begin{array}{cc} \; & =\underset{\rho \geq 0}{\sup}\;\left\{\rho \;\;\;\;\;\;\underset{\begin{array}{c} P_{X}:\\ E[b(X)]\leq \theta \end{array}}{\sup}\;\;\;\;\;\;I_{\frac{1}{1+\rho }}^{c}\left(X;Y\right)-\rho \;R\right\} \end{array}$$
  (280) $$\begin{array}{cc} \; & =\underset{\rho \geq 0}{\sup}\;\left\{\rho \;C_{\frac{1}{1+\rho }}^{c}\left(\theta \right)-\rho \;R\right\} \end{array}$$
  (281) $$\begin{array}{cc} \; & =\underset{\rho \geq 0}{\sup}\;\left\{-\rho \;R+\rho \min_{\nu \geq 0}\left\{\nu \;\theta +A_{\frac{1}{1+\rho }}\left(\nu \right)\right\}\right\}\\ \; & =\underset{\rho \geq 0}{\sup}\;\left\{-\rho \;R+\min_{\nu \geq 0}\left\{\rho \;\nu \;\theta \right.\right. \end{array}$$
  (282) $$\begin{array}{cc} \; & \;\left.\left.+\max_{X}\left\{\rho \;I_{\frac{1}{1+\rho }}\left(X;Y\right)+\left(1+\rho \right)\log E\left[\exp\left(-\frac{\rho \;\nu }{1+\rho }b\left(X\right)\right)\right]\right\}\right\}\right\}, \end{array}$$
  where (279), (281) and (282) follow from (256), (208) and (206), respectively.
72. In parallel to (278)–(281),
  (283) $$\begin{array}{cc} E_{r}\left(R,\theta \right) & =\underset{\begin{array}{c} P_{X}:\\ E[b(X)]\leq \theta \end{array}}{\sup}E_{r}\left(R,P_{X}\right) \end{array}$$
  (284) $$\begin{array}{cc} \; & =\underset{\rho \in [0,1]}{\sup}\;\left\{\rho \;\;\;\;\;\;\underset{\begin{array}{c} P_{X}:\\ E[b(X)]\leq \theta \end{array}}{\sup}\;\;\;\;\;\;I_{\frac{1}{1+\rho }}^{c}\left(X;Y\right)-\rho \;R\right\} \end{array}$$
  (285) $$\begin{array}{cc} \; & =\underset{\rho \in [0,1]}{\sup}\;\left\{\rho \;C_{\frac{1}{1+\rho }}^{c}\left(\theta \right)-\rho \;R\right\}, \end{array}$$
  where (284) follows from (270). In particular, if we define the critical rate and the cutoff rate as
  (286) $$\begin{array}{cc} R_{c} & =\underset{\begin{array}{c} P_{X}:\\ E[b(X)]\leq \theta \end{array}}{\sup}R_{c}\left(P_{X}\right), \end{array}$$
  (287) $$\begin{array}{cc} R_{0} & =\underset{\begin{array}{c} P_{X}:\\ E[b(X)]\leq \theta \end{array}}{\sup}I_{\frac{1}{2}}^{c}\left(X;Y\right), \end{array}$$
  respectively, then it follows from (270) that
  (288) $$\begin{array}{c} E_{r}\left(R\right)=R_{0}-R,\;R\in \left[0,R_{c}\right]. \end{array}$$
  Summarizing, the evaluation of $E_{\mathrm{sp}}\left(R,\theta \right)$ and $E_{r}\left(R,\theta \right)$ can be accomplished by the method proposed in Section 8, at the heart of which is the maximization in (206) involving α-mutual information instead of Augustin–Csiszár mutual information. In Section 11 and Section 12, we illustrate the evaluation of the error exponent functions with two important additive-noise examples.

## 11. Additive Independent Gaussian Noise; Input Power Constraint

We illustrate the procedure in Item 58 by taking Example 6 considerably further.

**Figure 2:**
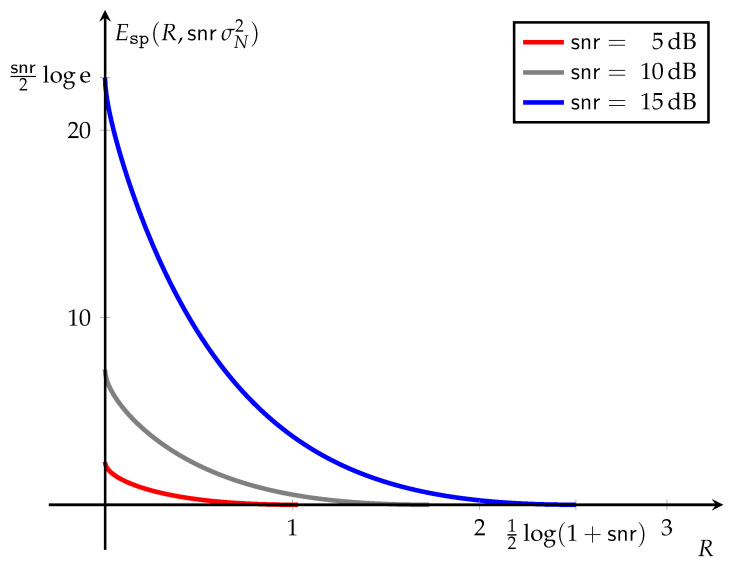
$E_{\mathrm{sp}}\left(R,\mathrm{snr}\;\sigma_{N}^{2}\right)$ in (312) and (313); logarithms in base 2.

73. Suppose A=B=R, $b\left(x\right)=x^{2}$, and $P_{Y|X=a}=N\;\left(a,\sigma_{N}^{2}\right)$. We start by testing whether we can find $R_{X}^{\nu }\in P_{A}$ such that its α-response satisfies (230). Naturally, it makes sense to try $R_{X}^{\nu }=N\;\left(0,\sigma^{2}\right)$ for some yet to be determined $\sigma^{2}$. As we saw in Example 6, this choice implies that its α-response is $R_{Y[\alpha ]}^{\nu }=N\;\left(0,\alpha \;\sigma^{2}+\sigma_{N}^{2}\right)$. Specializing Example 4, we obtain
  (289) $$\begin{array}{cc} \; & D_{\alpha }\left(P_{Y|X=x}\;∥\;R_{Y[\alpha ]}^{\nu }\right)=D_{\alpha }\left(N\;\left(x,\sigma_{N}^{2}\right)\;∥\;N\;\left(0,\alpha \;\sigma^{2}+\sigma_{N}^{2}\right)\right) \end{array}$$
  (290) $$\begin{array}{cc} \; & =\frac{1}{2}\log\left(1+\frac{\alpha \;\sigma^{2}}{\sigma_{N}^{2}}\right)-\frac{1}{2(1-\alpha )}\log\left(1+\frac{\alpha \left(1-\alpha \right)\sigma^{2}}{\alpha^{2}\sigma^{2}+\sigma_{N}^{2}}\right)+\frac{1}{2}\frac{\alpha x^{2}}{\alpha^{2}\sigma^{2}+\sigma_{N}^{2}}\log e. \end{array}$$
  Therefore, (230) is indeed satisfied with
  (291) $$\begin{array}{cc} c_{\alpha }\left(\nu \right) & =\frac{1}{2}\log\left(1+\frac{\alpha \;\sigma^{2}}{\sigma_{N}^{2}}\right)-\frac{1}{2(1-\alpha )}\log\left(1+\frac{\alpha \left(1-\alpha \right)\sigma^{2}}{\alpha^{2}\sigma^{2}+\sigma_{N}^{2}}\right), \end{array}$$
  (292) $$\begin{array}{cc} \nu & =\frac{1}{2}\frac{\alpha }{\alpha^{2}\sigma^{2}+\sigma_{N}^{2}}\log e, \end{array}$$
  where (292) follows if we choose the variance of the auxiliary input as
  (293) $$\begin{array}{cc} \sigma^{2}\; & =\frac{\log e}{2\;\alpha \;\nu }-\frac{\sigma_{N}^{2}}{\alpha^{2}} \end{array}$$
  (294) $$\begin{array}{cc} \; & =\frac{\sigma_{N}^{2}}{\alpha^{2}}\left(\frac{\alpha }{\lambda }-1\right). \end{array}$$
  In (294) we have introduced an alternative, more convenient, parametrization for the Lagrange multiplier
  (295) $$\begin{array}{c} \lambda =\frac{2\;\nu \;\sigma_{N}^{2}}{\log e}\in \left(0,\alpha \right). \end{array}$$
  In conclusion, with the choice in (293), $N\;\left(0,\sigma^{2}\right)$ attains the maximum in (206), and in view of (231), $A_{\alpha }\left(\nu \right)$ is given by the right side of (291) substituting $\sigma^{2}$ by (293). Therefore, we have
  (296) $$\begin{array}{cc} \; & \nu \;\theta +A_{\alpha }\left(\nu \right)=\frac{\lambda }{2}\mathrm{snr}\log e+c_{\alpha }\left(\frac{\lambda \log e}{2\;\sigma_{N}^{2}}\right) \end{array}$$
  (297) $$\begin{array}{cc} \; & =\frac{\lambda }{2}\mathrm{snr}\log e+\frac{1}{2}\log\left(1+\frac{1}{\lambda }-\frac{1}{\alpha }\right)-\frac{1}{2(1-\alpha )}\log\left(\alpha -\lambda (1-\alpha )\right)+\frac{\log\alpha }{1-\alpha }, \end{array}$$
  where we denoted $\mathrm{snr}=\frac{\theta }{\sigma_{N}^{2}}.$
  In accordance with Theorem 16 all that remains is to minimize (297) with respect to ν, or equivalently, with respect to λ. Differentiating (297) with respect to λ, the minimum is achieved at $\lambda^{*}$ satisfying
  (298) $$\begin{array}{c} \mathrm{snr}=\frac{1}{\lambda^{*}}\frac{\alpha -\lambda^{*}}{\alpha -\lambda^{*}+\alpha \;\lambda^{*}}, \end{array}$$
  whose only valid root (obtained by solving a quadratic equation) is
  (299) $$\begin{array}{cc} \lambda^{*} & =\frac{1+\alpha \;\mathrm{snr}-\alpha \;\Delta }{2\;\mathrm{snr}\;(1-\alpha )}\in \left(0,\alpha \right), \end{array}$$
  with Δ defined in (118). So, for α∈(0,1), (208) becomes
  (300) $$\begin{array}{cc} C_{\alpha }^{c}\left(\mathrm{snr}\;\sigma_{N}^{2}\right) & =\frac{1+\alpha \;\mathrm{snr}-\alpha \;\Delta }{4(1-\alpha )}\log e+\frac{1}{2}\log\left(1+\frac{2\;\mathrm{snr}\;(1-\alpha )}{1+\alpha \;\mathrm{snr}-\alpha \;\Delta }-\frac{1}{\alpha }\right)\\ \; & \;-\frac{1}{2(1-\alpha )}\log\left(\frac{\alpha \;\mathrm{snr}+\alpha \;\Delta -1}{2\;\mathrm{snr}\;\alpha^{2}}\right). \end{array}$$
  Letting $\alpha =\frac{1}{1+\rho }$, we obtain
  (301) $$\begin{array}{cc} C_{\frac{1}{1+\rho }}^{c}\left(\mathrm{snr}\;\sigma_{N}^{2}\right) & =\frac{\mathrm{snr}}{2\;\rho }\left(1-\beta \right)\log e+\frac{1}{2}\log\left(1+\beta \;\mathrm{snr}\right)-\frac{1+\rho }{2\;\rho }\log\left((1+\rho )\beta \right), \end{array}$$
  with
  (302) $$\begin{array}{c} \beta =\frac{1}{2}\left(1-\frac{1}{\alpha \;\mathrm{snr}}+\frac{\Delta }{\mathrm{snr}}\right)=\frac{1}{2}\left(1-\frac{1+\rho }{\mathrm{snr}}+\sqrt{\frac{4}{\mathrm{snr}}+{\left(\frac{1+\rho }{\mathrm{snr}}-1\right)}^{2}}\right). \end{array}$$
74. Alternatively, it is instructive to apply Theorem 18 to the current Gaussian/quadratic cost setting. Suppose we let $Q_{X}^{*}=N\;\left(0,\sigma^{*2}\right)$, where $\sigma^{*2}$ is to be determined. With the aid of the formulas
  (303) $$\begin{array}{cc} E\left[X^{2}\;e^{-\mu X^{2}}\right] & =\frac{\sigma^{2}}{{\left(1+2\;\mu \;\sigma^{2}\right)}^{\frac{3}{2}}}, \end{array}$$
  (304) $$\begin{array}{cc} E\left[e^{-\mu X^{2}}\right] & =\frac{1}{\sqrt{1+2\;\mu \;\sigma^{2}}}, \end{array}$$
  where μ≥0, and $X∼N\;\left(0,\sigma^{2}\right)$, (217) becomes
  (305) $$\begin{array}{c} \frac{1}{\mathrm{snr}}=\frac{\sigma_{N}^{2}}{\sigma^{*2}}+\left(1-\alpha \right)\lambda^{*}, \end{array}$$
  upon substituting $\sigma^{2}\leftarrow \sigma^{*2}$ and
  (306) $$\begin{array}{c} \mu \leftarrow \nu^{*}\frac{1-\alpha }{\log e}=\lambda^{*}\frac{1-\alpha }{2\sigma_{N}^{2}}. \end{array}$$
  Likewise (218) translates into (291) and (292) with $\left(\nu ,\sigma^{2}\right)\leftarrow \left(\nu^{*},\sigma^{*2}\right)$, namely,
  (307) $$\begin{array}{cc} c_{\alpha }\left(\nu^{*}\right) & =\frac{1}{2}\log\left(1+\frac{\alpha \;\sigma^{*2}}{\sigma_{N}^{2}}\right)-\frac{1}{2(1-\alpha )}\log\left(1+\frac{\alpha \left(1-\alpha \right)\sigma^{*2}}{\alpha^{2}\sigma^{*2}+\sigma_{N}^{2}}\right), \end{array}$$
  (308) $$\begin{array}{cc} \lambda^{*} & =\frac{\alpha \sigma_{N}^{2}}{\alpha^{2}\sigma^{*2}+\sigma_{N}^{2}}. \end{array}$$
  Eliminating $\sigma^{*2}$ from (305) by means of (308) results in (299) and the same derivation that led to (300) shows that it is equal to $\nu^{*}\theta +c_{\alpha }\left(\nu^{*}\right)$.
75. Applying Theorem 17, we can readily find the input distribution, $P_{X}^{*}$, that attains $C_{\alpha }^{c}\left(\theta \right)$ as well as its 〈α〉-response $P_{Y}^{*}$ (recall the notation in Item 53). According to Example 2, $P_{Y}^{*}$, the α-response to $Q_{X}^{*}$ is Gaussian with zero mean and variance
  (309) $$\begin{array}{cc} \sigma_{N}^{2}+\alpha \;\sigma^{*2} & =\sigma_{N}^{2}\left(1+\frac{1}{\lambda^{*}}-\frac{1}{\alpha }\right) \end{array}$$
  (310) $$\begin{array}{cc} \; & =\frac{\sigma_{N}^{2}}{2}\left(2-\frac{1}{\alpha }+\Delta +\mathrm{snr}\right), \end{array}$$
  where (309) follows from (308) and (310) follows by using the expression for Δ in (118). Note from Example 7 that $P_{Y}^{*}$ is nothing but the 〈α〉-response to $N\;\left(0,\mathrm{snr}\;\sigma_{N}^{2}\right)$. We can easily verify from Theorem 17 that indeed $P_{X}^{*}=N\;\left(0,\mathrm{snr}\;\sigma_{N}^{2}\right)$ since in this case (216) becomes
  (311) $$\begin{array}{c} {ı}_{P_{X}^{*}∥Q_{X}^{*}}\left(a\right)=-\left(1-\alpha \right)\nu^{*}\;a^{2}+\tau_{\alpha }, \end{array}$$
  which can only be satisfied by $P_{X}^{*}=N\;\left(0,\mathrm{snr}\;\sigma_{N}^{2}\right)$ in view of (305). As an independent confirmation, we can verify, after some algebra, that the right sides of (127) and (300) are identical.
  In fact, in the current Gaussian setting, we could start by postulating that the distribution that maximizes the Augustin–Csiszár mutual information under the second moment constraint does not depend on α and is given by $P_{X}^{*}=N\;\left(0,\theta \right)$. Its 〈α〉-response $P_{Y\langle \alpha \rangle }^{*}$ was already obtained in Example 7. Then, an alternative method to find $C_{\alpha }^{c}\left(\theta \right)$, given in Section 6.2 of [43], is to follow the approach outlined in Item 53. To validate the choice of $P_{X}^{*}$ we must show that it maximizes $B(P_{X},P_{Y\langle \alpha \rangle }^{*})$ (in the notation introduced in (199)) among the subset of $P_{A}$ which satisfies $E[X^{2}]\leq \theta$. This follows from the fact that $D_{\alpha }\left(P_{Y|X=x}∥P_{Y\langle \alpha \rangle }^{*}\right)$ is an affine function of $x^{2}$.
76. Let’s now use the result in Item 73 to evaluate, with a novel parametrization, the error exponent functions for the Gaussian channel under an average power constraint.
  **Theorem** **23.** *Let A=B=R, $b\left(x\right)=x^{2}$, and $P_{Y|X=a}=N\;\left(a,\sigma_{N}^{2}\right)$. Then, for β∈[0,1],*
  (312) $$\begin{array}{cc} E_{\mathrm{sp}}\left(R,\mathrm{snr}\;\sigma_{N}^{2}\right) & =\frac{\mathrm{snr}}{2}\left(1-\beta \right)\log e-\frac{1}{2}\log\left(1+\mathrm{snr}\;\beta (1-\beta )\right), \end{array}$$
  (313) $$\begin{array}{cc} R & =\frac{1}{2}\log\left(1+\frac{\beta^{2}}{\beta \left(1-\beta \right)+\frac{1}{\mathrm{snr}}}\right). \end{array}$$
*The critical rate and cutoff rate are, respectively,*
  (314) $$\begin{array}{cc} R_{c}\; & =\frac{1}{2}\log\left(\frac{1}{2}+\frac{\mathrm{snr}}{4}+\frac{1}{2}\sqrt{1+\frac{{\mathrm{snr}}^{2}}{4}}\right), \end{array}$$
  (315) $$\begin{array}{cc} R_{0} & =\frac{1}{2}\left(1+\frac{\mathrm{snr}}{2}-\sqrt{1+\frac{{\mathrm{snr}}^{2}}{4}}\right)\;\log e+\frac{1}{2}\log\left(\frac{1}{2}+\frac{1}{2}\sqrt{1+\frac{{\mathrm{snr}}^{2}}{4}}\right). \end{array}$$
  **Proof.**  Expression (315) for the cutoff rate follows by letting ρ=1 in (301) and (302). The supremum in (281) is attained by $\rho^{*}\geq 0$ that satisfies (recall the concavity result in Theorem 9-(a))
  (316) $$\begin{array}{cc} R & =\frac{d}{d\rho }\;\rho \;C_{\frac{1}{1+\rho }}^{c}\left(\mathrm{snr}\;\sigma_{N}^{2}\right){|}_{\rho \leftarrow \rho^{*}} \end{array}$$
  (317) $$\begin{array}{cc} \; & =\frac{1}{2}\log\left(\mathrm{snr}+\frac{1}{\beta }\right)-\frac{1}{2}\log\left(1+\rho^{*}\right), \end{array}$$
  obtained after a dose of symbolic computation working with (301). In particular, letting $\rho^{*}=1$, we obtain the critical rate in (314). Note that if in (302) we substitute $\rho \leftarrow \rho^{*}$, with $\rho^{*}$ given as a function of *R*, snr and β by (317), we end up with an equation involving *R*, snr, and β. We proceed to verify that that equation is, in fact, (312). By solving a quadratic equation, we can readily check that (302) is the positive root of
  (318) $$\begin{array}{c} 1+\rho =\mathrm{snr}\left(1-\beta \right)+\frac{1}{\beta }. \end{array}$$
  If we particularize (318) to $\rho \leftarrow \rho^{*}$, with $\rho^{*}$ given by (317), namely,
  (319) $$\begin{array}{c} \rho^{*}=-1+\exp\left(-2R\right)\left(\mathrm{snr}+\frac{1}{\beta }\right), \end{array}$$
  we obtain
  (320) $$\begin{array}{c} \exp\left(2R\right)=\frac{\mathrm{snr}\;\beta +1}{\mathrm{snr}\;\beta (1-\beta )+1}, \end{array}$$
  which is (313). Notice that the right side of (320) is monotonic increasing in β>0 ranging from 1 (for β=0) to 1+snr (for β=1). Therefore, β∈[0,1] spans the whole gamut of values of *R* of interest.
  Assembling (281), (301) and (317), we obtain
  $$\begin{array}{cc} \; & \;E_{\mathrm{sp}}\left(R,\mathrm{snr}\;\sigma_{N}^{2}\right) \end{array}$$
  (321) $$\begin{array}{cc} \; & =-\rho^{*}R+\frac{\mathrm{snr}}{2}\left(1-\beta \right)\log e+\frac{\rho^{*}}{2}\log\left(1+\beta \;\mathrm{snr}\right)-\frac{1+\rho^{*}}{2}\log\left((1+\rho^{*})\beta \right)\\ \; & =-\rho^{*}R+\frac{\mathrm{snr}}{2}\left(1-\beta \right)\log e+\frac{\rho^{*}}{2}\log\left(1+\beta \;\mathrm{snr}\right)-\frac{1+\rho^{*}}{2}\log\beta \end{array}$$
  (322) $$\begin{array}{cc} \; & \;\;\;\;\;\;+\left(1+\rho^{*}\right)R-\frac{1+\rho^{*}}{2}\log\left(\mathrm{snr}+\frac{1}{\beta }\right) \end{array}$$
  (323) $$\begin{array}{cc} \; & =R+\frac{\mathrm{snr}}{2}\left(1-\beta \right)\log e-\frac{1}{2}\log\left(1+\beta \;\mathrm{snr}\right) \end{array}$$
  (324) $$\begin{array}{cc} \; & =\frac{\mathrm{snr}}{2}\left(1-\beta \right)\log e-\frac{1}{2}\log\left(1+\mathrm{snr}\;\beta (1-\beta )\right), \end{array}$$
  where (324) follows by substituting (313) on the left side. □
  Note that the parametric expression in (312) and (313) (shown in Figure 2) is, in fact, a closed-form expression for $E_{\mathrm{sp}}\left(R,\mathrm{snr}\;\sigma_{N}^{2}\right)$ since we can invert (313) to obtain
  (325) $$\begin{array}{c} \beta =\frac{1}{2}\left(1-\exp(-2\;R)\right)\left(1+\sqrt{1+\frac{4}{\mathrm{snr}\;(1-\exp(-2\;R))}}\right). \end{array}$$
  The random coding error exponent is
  (326) $$\begin{array}{c} E_{r}\left(R,\theta \right)=\left\{\begin{array}{cc} E_{\mathrm{sp}}\left(R,\theta \right), & R\in (R_{c},\frac{1}{2}\log\left(1+\mathrm{snr}\right));\\ R_{0}-R, & R\in [0,R_{c}], \end{array}\right. \end{array}$$
  with the critical rate $R_{c}$ and cutoff rate $R_{0}$ in (314) and (315), respectively. It can be checked that (326) coincides with the expression given by Gallager [9] (p. 340) where he optimizes (235) with respect to ρ and *r*, but not $P_{X}$, which he just assumes to be $P_{X}=N\;\left(0,\theta \right)$. The expression for $R_{c}$ in (314) can be found in (7.4.34) of [9]; $R_{0}$ in (314) is implicit in p. 340 of [9], and explicit in e.g., [69].
77. The expression for $E_{\mathrm{sp}}\left(R,\theta \right)$ in Theorem 23 has more structure than meets the eye. The analysis in Item 73 has shown that $E_{\mathrm{sp}}\left(R,P_{X}\right)$ is maximized over $P_{X}$ with second moment not exceeding θ by $P_{X}^{*}=N\;\left(0,\theta \right)$ regardless of $R\in \left(0,\frac{1}{2}\log\left(1+\mathrm{snr}\right)\right)$. The fact that we have found a closed-form expression for (254) when evaluated at such input probability measure and $P_{Y|X=a}=N\;\left(a,\sigma_{N}^{2}\right)$ is indicative that the minimum therein is attained by a Gaussian random transformation $Q_{Y|X}^{*}$. This is indeed the case: define the random transformation
  (327) $$\begin{array}{cc} Q_{Y|X=a}^{*} & =N\;\left(\beta \;a,\sigma_{1}^{2}\right), \end{array}$$
  (328) $$\begin{array}{cc} \frac{\sigma_{1}^{2}}{\sigma_{N}^{2}} & =1+\mathrm{snr}\;\beta (1-\beta ). \end{array}$$
  In comparison with the nominal random transformation $P_{Y|X=a}=N\;\left(a,\sigma_{N}^{2}\right)$, this channel attenuates the input and contaminates it with a more powerful noise. Then,
  (329) $$\begin{array}{cc} I(P_{X}^{*},Q_{Y|X}^{*}) & =\frac{1}{2}\log\left(1+\frac{\beta^{2}}{\beta \left(1-\beta \right)+\frac{1}{\mathrm{snr}}}\right)=R. \end{array}$$
  Furthermore, invoking (33), we get
  (330) $$\begin{array}{cc} D(Q_{Y|X}^{*}∥P_{Y|X}|P_{X}^{*}) & =E\left[D\left(N\;\left(\beta X^{*},\sigma_{1}^{2}\right)\;∥\;N\;\left(X^{*},\sigma_{N}^{2}\right)\right)\right] \end{array}$$
  (331) $$\begin{array}{cc} \; & =\frac{1}{2}\left({\left(\beta -1\right)}^{2}\mathrm{snr}+\frac{\sigma_{1}^{2}}{\sigma_{N}^{2}}-1\right)\log e-\frac{1}{2}\log\frac{\sigma_{1}^{2}}{\sigma_{N}^{2}} \end{array}$$
  (332) $$\begin{array}{cc} \; & =\frac{\mathrm{snr}}{2}\left(1-\beta \right)\log e-\frac{1}{2}\log\left(1+\mathrm{snr}\;\beta (1-\beta )\right) \end{array}$$
  (333) $$\begin{array}{cc} \; & =E_{\mathrm{sp}}\left(R,\mathrm{snr}\;\sigma_{N}^{2}\right), \end{array}$$
  where (333) is (312). Therefore, $Q_{Y|X}^{*}$ does indeed achieve the minimum in (254) if $P_{Y|X=a}=N\;\left(a,\sigma_{N}^{2}\right)$ and $P_{X}^{*}=N\;\left(0,\theta \right)$. So, the most likely error mechanism is the result of atypically large noise strength and an attenuated received signal. Both effects cannot be combined into additional noise variance: there is no $\sigma^{2}>0$ such that $Q_{Y|X=a}=N\;\left(a,\sigma^{2}\right)$ achieves the minimum in (254).

## 12. Additive Independent Exponential Noise; Input-Mean Constraint

This section finds the sphere-packing error exponent for the additive independent exponential noise channel under an input-mean constraint.

**Figure 3:**
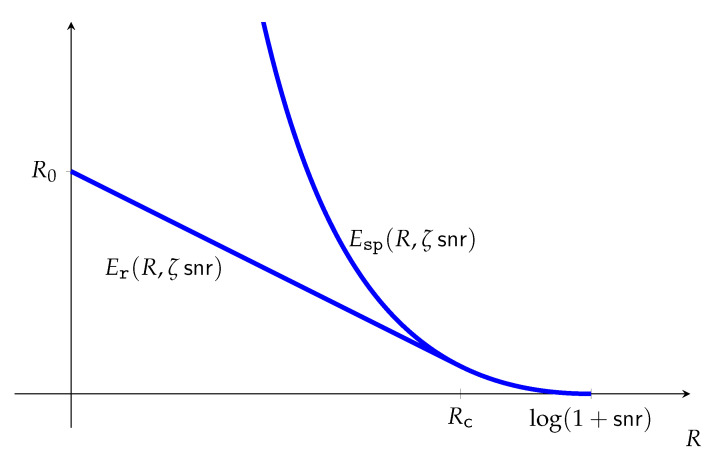
Error exponent functions in (354), (355) and (376).

78. Suppose that A=B=[0,∞), b(x)=x, and
  (334) $$\begin{array}{c} Y=X+N, \end{array}$$
  where *N* is exponentially distributed, independent of *X*, and E[N]=ζ. Therefore $P_{Y|X=a}$ has density
  (335) $$\begin{array}{c} p_{Y|X=a}\left(t\right)=\frac{1}{\zeta }e^{-\frac{t-a}{\zeta }}1\left\{t\geq a\right\}. \end{array}$$
  It is shown in [70,71] that
  (336) $$\begin{array}{cc} \max_{X:E[X]\leq \theta }I\left(X;X+N\right) & =\log\left(1+\mathrm{snr}\right), \end{array}$$
  (337) $$\begin{array}{cc} \mathrm{snr} & =\frac{\theta }{\zeta }, \end{array}$$
  achieved by a mixed random variable with density
  (338) $$\begin{array}{c} f_{X}^{*}\left(t\right)=\frac{\zeta }{\zeta +\theta }\;\delta \left(t\right)+\frac{\theta }{{\left(\zeta +\theta \right)}^{2}}\;e^{-t/(\zeta +\theta )}1\left\{t>0\right\}. \end{array}$$
  To determine $C_{\alpha }^{c}\left(\mathrm{snr}\;\zeta \right)$, α∈(0,1), we invoke Theorem 18. A sensible candidate for the auxiliary input distribution $Q_{X}^{*}$ is a mixed random variable with density
  (339) $$\begin{array}{cc} q_{X}^{*}\left(t\right) & =\Gamma^{*}\;\delta \left(t\right)+\left(1-\Gamma^{*}\right)\frac{1}{\mu }e^{-t/\mu }\;1\left\{t>0\right\}, \end{array}$$
  (340) $$\begin{array}{cc} \mu & =\frac{\zeta }{\alpha \;\Gamma^{*}}, \end{array}$$
  where $\Gamma^{*}\in \left(0,1\right)$ is yet to be determined. This is an attractive choice because its α-response, $Q_{Y[\alpha ]}^{*}$, is particularly simple: exponential with mean $\alpha \;\mu =\frac{\zeta }{\Gamma^{*}}$, as we can verify using Laplace transforms. Then, if *Z* is exponential with unit mean, with the aid of Example 5, we can write
  (341) $$\begin{array}{cc} D_{\alpha }\left(P_{Y|X=x}\;∥\;Q_{Y[\alpha ]}^{*}\right) & =D_{\alpha }\left(\zeta \;Z+x\;∥\;\alpha \;\mu \;Z\right) \end{array}$$
  (342) $$\begin{array}{cc} \; & =\frac{x}{\alpha \;\mu }\log e+\log\frac{\alpha \;\mu }{\zeta }+\frac{1}{1-\alpha }\log\left(\alpha +\left(1-\alpha \right)\frac{\zeta }{\alpha \;\mu }\right) \end{array}$$
  (343) $$\begin{array}{cc} \; & =\frac{\Gamma^{*}x}{\zeta }\log e-\log\Gamma^{*}+\frac{1}{1-\alpha }\log\left(\alpha +\left(1-\alpha \right)\Gamma^{*}\right). \end{array}$$
  So, (218) is satisfied with
  (344) $$\begin{array}{cc} \nu^{*} & =\frac{\Gamma^{*}}{\zeta }\;\log e, \end{array}$$
  (345) $$\begin{array}{cc} c_{\alpha }\left(\nu^{*}\right) & =\frac{1}{1-\alpha }\log\left(\alpha +\left(1-\alpha \right)\Gamma^{*}\right)-\log\Gamma^{*}. \end{array}$$
  To evaluate (217), it is useful to note that if γ>−1, then
  (346) $$\begin{array}{cc} E\left[Ze^{-\gamma Z}\right] & =\frac{1}{{\left(1+\gamma \right)}^{2}}, \end{array}$$
  (347) $$\begin{array}{cc} E\left[e^{-\gamma Z}\right] & =\frac{1}{1+\gamma }. \end{array}$$
  Therefore, the left side of (217) specializes to, with ${\bar{X}}^{*}∼Q_{X}^{*}$,
  (348) $$\begin{array}{cc} E\left[b\left({\bar{X}}^{*}\right)\exp\left(-\left(1-\alpha \right)\nu^{*}\;b\left({\bar{X}}^{*}\right)\right)\right]\; & =\frac{\mu (1-\Gamma^{*})}{{\left(1+\mu \left(1-\alpha \right)\frac{\nu^{*}}{\log e}\right)}^{2}} \end{array}$$
  (349) $$\begin{array}{cc} \; & =\zeta \;\alpha \left(\frac{1}{\Gamma^{*}}-1\right), \end{array}$$
  while the expectation on the right side of (217) is given by
  (350) $$\begin{array}{cc} E\left[\exp\left(-\left(1-\alpha \right)\nu^{*}\;b\left({\bar{X}}^{*}\right)\right)\right]\; & =\alpha +\Gamma^{*}-\alpha \Gamma^{*}. \end{array}$$
  Therefore, (217) yields
  (351) $$\begin{array}{c} \mathrm{snr}=\frac{1}{\Gamma^{*}}-\frac{1}{\alpha +\left(1-\alpha \right)\Gamma^{*}} \end{array}$$
  whose solution is
  (352) $$\begin{array}{c} \Gamma^{*}=\frac{1}{2\rho \;\mathrm{snr}}\left(\sqrt{{\left(1+\mathrm{snr}\right)}^{2}+4\rho \;\mathrm{snr}}-1-\mathrm{snr}\right), \end{array}$$
  with $\rho =\frac{1-\alpha }{\alpha }$. So, finally, (220), (344) and (345) give the closed-form expression
  (353) $$\begin{array}{c} C_{\alpha }^{c}\left(\theta \right)=\mathrm{snr}\;\Gamma^{*}\log e-\log\Gamma^{*}+\frac{1}{1-\alpha }\log\left(\alpha +\left(1-\alpha \right)\Gamma^{*}\right). \end{array}$$
  As in Item 73, we can postulate an auxiliary distribution that satisfies (230) for every ν≥0. This is identical to what we did in (341)–(343) except that now (344) and (345) hold for generic ν and Γ. Then, (351) is the result of solving $\theta =-{\dot{c}}_{\alpha }\left(\nu^{*}\right)$, which is, in fact, somewhat simpler than obtaining it through (217).
79. We proceed to get a very simple parametric expression for $E_{\mathrm{sp}}\left(R,\theta \right)$.
  **Theorem** **24.** *Let A=B=[0,∞), b(x)=x, and Y=X+N, with N exponentially distributed, independent of X, and E[N]=ζ. Then, under the average cost constraint E[b(X)]≤ζsnr,*
  (354) $$\begin{array}{cc} E_{\mathrm{sp}}\left(R,\zeta \;\mathrm{snr}\right) & =\left(\frac{1}{\eta }-1\right)\log e+\log\eta , \end{array}$$
  (355) $$\begin{array}{cc} R & =\log(1+\eta \;\mathrm{snr}), \end{array}$$
  *where η∈(0,1].*
  **Proof.**  Rewriting (353), results in
  (356) $$\begin{array}{c} \rho \;C_{\frac{1}{1+\rho }}^{c}\left(\theta \right)=\rho \;\mathrm{snr}\;\Gamma^{*}\log e-\rho \log\Gamma^{*}+\left(1+\rho \right)\log\frac{1+\rho \;\Gamma^{*}}{1+\rho }, \end{array}$$
  which is monotonically decreasing with ρ. With ${\dot{\Gamma }}^{*}=\frac{\partial }{\partial \rho }\Gamma^{*}\left(\rho ,\mathrm{snr}\right)$, the counterpart of (317) is now
  (357) $$\begin{array}{cc} R & =\frac{d}{d\rho }\rho \;C_{\frac{1}{1+\rho }}^{c}\left(\theta \right){|}_{\rho \leftarrow \rho^{*}} \end{array}$$
  (358) $$\begin{array}{cc} \; & =\left(\Gamma^{*}+\rho^{*}{\dot{\Gamma }}^{*}\right)\left(\mathrm{snr}-\frac{1}{\Gamma^{*}}+\frac{1+\rho^{*}}{1+\rho^{*}\Gamma^{*}}\right)\log e+\log\frac{1+\rho^{*}\Gamma^{*}}{\Gamma^{*}+\rho^{*}\;\Gamma^{*}} \end{array}$$
  (359) $$\begin{array}{cc} \; & =\left(\Gamma^{*}+\rho^{*}{\dot{\Gamma }}^{*}\right)\left(\mathrm{snr}+\frac{1}{\Gamma^{*}}\frac{\Gamma^{*}-1}{1+\rho^{*}\Gamma^{*}}\right)\log e+\log\frac{1+\rho^{*}\Gamma^{*}}{\Gamma^{*}+\rho^{*}\;\Gamma^{*}} \end{array}$$
  (360) $$\begin{array}{cc} \; & =\log\frac{1+\rho^{*}\Gamma^{*}}{\Gamma^{*}+\rho^{*}\;\Gamma^{*}}, \end{array}$$
  where the drastic simplification in (360) occurs because, with the current parametrization, (351) becomes
  (361) $$\begin{array}{c} 1-\Gamma^{*}=\left(1+\rho^{*}\;\Gamma^{*}\right)\;\Gamma^{*}\;\mathrm{snr}. \end{array}$$
  Now we go ahead and express both $\rho^{*}$ and $\Gamma^{*}$ as functions of snr and *R* exclusively. We may rewrite (357)–(360) as
  (362) $$\begin{array}{c} \rho^{*}\;\Gamma^{*}=\frac{\exp\left(-R\right)-\Gamma^{*}}{1-\exp(-R)}, \end{array}$$
  which, when plugged in (361), results in
  (363) $$\begin{array}{cc} \Gamma^{*} & =\frac{1}{\mathrm{snr}}\left(1-\exp(-R)\right)<1, \end{array}$$
  (364) $$\begin{array}{cc} \rho^{*} & =\frac{(1+\mathrm{snr})\exp(-R)-1}{{\left(1-\exp(-R)\right)}^{2}}>0, \end{array}$$
  where the inequalities in (363) and (364) follow from R<log(1+snr). So, in conclusion,
  (365) $$\begin{array}{cc} E_{\mathrm{sp}}\left(R,\theta \right) & =\max_{\rho \geq 0}\;\left\{\rho \;C_{\frac{1}{1+\rho }}^{c}\left(\theta \right)-\rho \;R\right\} \end{array}$$
  (366) $$\begin{array}{cc} \; & =\rho^{*}\;C_{\frac{1}{1+\rho^{*}}}^{c}\left(\theta \right)-\rho^{*}\;R \end{array}$$
  (367) $$\begin{array}{cc} \; & =\rho^{*}\;\mathrm{snr}\;\Gamma^{*}\log e-\rho^{*}\log\Gamma^{*}+\left(1+\rho^{*}\right)\log\frac{1+\rho^{*}\Gamma^{*}}{1+\rho^{*}}-\rho^{*}\;R \end{array}$$
  (368) $$\begin{array}{cc} \; & =\rho^{*}\;\mathrm{snr}\;\Gamma^{*}\log e-\rho^{*}\log\Gamma^{*}+\left(1+\rho^{*}\right)\left(R+\log\Gamma^{*}\right)-\rho^{*}\;R \end{array}$$
  (369) $$\begin{array}{cc} \; & =\rho^{*}\;\mathrm{snr}\;\Gamma^{*}\log e+\log\Gamma^{*}+R \end{array}$$
  (370) $$\begin{array}{cc} \; & =\left(\frac{\mathrm{snr}}{\exp(R)-1}-1\right)\log e+\log\frac{\exp(R)-1}{\mathrm{snr}} \end{array}$$
  (371) $$\begin{array}{cc} \; & =\left(\frac{1}{\eta }-1\right)\log e+\log\eta , \end{array}$$
  where we have introduced
  (372) $$\begin{array}{c} \eta =\frac{\exp(R)-1}{\mathrm{snr}}=\frac{\Gamma^{*}}{1-\mathrm{snr}\;\Gamma^{*}}. \end{array}$$
  Evidently, the left identity in (372) is the same as (355). □
  The critical rate and the cutoff rate are obtained by particularizing (360) and (356) to $\rho^{*}=1$ and ρ=1, respectively. This yields
  (373) $$\begin{array}{cc} R_{c} & =\log\frac{1+\Gamma_{1}^{*}}{2\;\Gamma_{1}^{*}}, \end{array}$$
  (374) $$\begin{array}{cc} R_{0} & =\mathrm{snr}\;\Gamma_{1}^{*}\log e-\log\left(4\;\Gamma_{1}^{*}\right)+2\log\left(1+\;\Gamma_{1}^{*}\right), \end{array}$$
  (375) $$\begin{array}{cc} \Gamma_{1}^{*} & =\frac{\sqrt{{\left(1+\mathrm{snr}\right)}^{2}+4\;\mathrm{snr}}-1-\mathrm{snr}}{2\;\mathrm{snr}}. \end{array}$$
  As in (326), the random coding error exponent is
  (376) $$\begin{array}{c} E_{r}\left(R,\zeta \;\mathrm{snr}\right)=\left\{\begin{array}{cc} E_{\mathrm{sp}}\left(R,\zeta \;\mathrm{snr}\right), & R\in (R_{c},\log\left(1+\mathrm{snr}\right));\\ R_{0}-R, & R\in [0,R_{c}], \end{array}\right. \end{array}$$
  with the critical rate $R_{c}$ and cutoff rate $R_{0}$ in (373) and (375), respectively. This function is shown along with $E_{\mathrm{sp}}\left(R,\zeta \;\mathrm{snr}\right)$ in Figure 3 for snr=3.
80. In parallel to Item 77, we find the random transformation that explains the most likely mechanism to produce errors at every rate *R*, namely the minimizer of (254) when $P_{X}=P_{X}^{*}$, the maximizer of the Augustin–Csiszár mutual information of order α. In this case, $P_{X}^{*}$ is not as trivial to guess as in Section 11, but since we already found $Q_{X}^{*}$ in (339) with $\Gamma =\Gamma^{*}$, we can invoke Theorem 17 to show that the density of $P_{X}^{*}$ achieving the maximal order-α Augustin–Csiszár mutual information is
  (377) $$\begin{array}{c} p_{X}^{*}\left(t\right)=\frac{\Gamma^{*}}{\alpha +\left(1-\alpha \right)\Gamma^{*}}\;\delta \left(t\right)+\frac{1-\Gamma^{*}}{\alpha +\left(1-\alpha \right)\Gamma^{*}}\;\frac{\alpha \;\Gamma^{*}}{\zeta }\;e^{-t\;\Gamma^{*}/\zeta }\;1\left\{t>0\right\}, \end{array}$$
  whose mean is, as it should,
  (378) $$\begin{array}{c} \frac{\alpha \;\zeta }{\Gamma^{*}}\frac{1-\Gamma^{*}}{\alpha +\left(1-\alpha \right)\Gamma^{*}}=\zeta \;\mathrm{snr}=\theta . \end{array}$$
  Let $Q_{Y}^{*}$ be exponential with mean θ+κ, and $Q_{Y|X=a}^{*}$ have density
  (379) $$\begin{array}{c} q_{Y|X=a}^{*}\left(t\right)=\frac{1}{\kappa }\;e^{-\frac{t-a}{\kappa }}\;1\left\{t\geq a\right\}, \end{array}$$
  with
  (380) $$\begin{array}{c} \kappa =\frac{\zeta }{\eta }, \end{array}$$
  and η as defined in (372). Using Laplace transforms, we can verify that $P_{X}^{*}\to Q_{Y|X}^{*}\to Q_{Y}^{*}$ where $P_{X}^{*}$ is the probability measure with density in (377). Let *Z* be unit-mean exponentially distributed. Writing mutual information as the difference between the output differential entropy and the noise differential entropy we get
  (381) $$\begin{array}{cc} I(P_{X}^{*},Q_{Y|X}^{*}) & =h((\theta +\kappa )Z)-h(\kappa Z) \end{array}$$
  (382) $$\begin{array}{cc} \; & =\log\left(1+\frac{\theta }{\kappa }\right) \end{array}$$
  (383) $$\begin{array}{cc} \; & =R, \end{array}$$
  in view of (363). Furthermore, using (335) and (379),
  (384) $$\begin{array}{cc} D(Q_{Y|X}^{*}\;∥\;P_{Y|X}|P_{X}^{*}) & =\log\frac{\zeta }{\kappa }+\left(\frac{\kappa }{\zeta }-1\right)\log e \end{array}$$
  (385) $$\begin{array}{cc} \; & =\log\eta +\left(\frac{1}{\eta }-1\right)\log e \end{array}$$
  (386) $$\begin{array}{cc} \; & =E_{\mathrm{sp}}\left(R,\zeta \;\mathrm{snr}\right), \end{array}$$
  where we have used (380) and (354). Therefore, we have shown that $Q_{Y|X}^{*}$ is indeed the minimizer of (254). In this case, the most likely mechanism for errors to happen is that the channel adds independent exponential noise with mean ζ/η, instead of the nominal mean ζ. In this respect, the behavior is reminiscent of that of the exponential timing channel for which the error exponent is dominated (at least above critical rate) by an exponential server which is slower than the nominal [72].

## 13. Recap

81. The analysis of the fundamental limits of noisy channels in the regime of vanishing error probability with blocklength growing without bound expresses channel capacity in terms of a basic information measure: the input–output mutual information maximized over the input distribution. In the regime of fixed nonzero error probability, the asymptotic fundamental limit is a function of not only capacity but channel dispersion [73], which is also expressible in terms of an information measure: the variance of the information density obtained with the capacity-achieving distribution. In the regime of exponentially decreasing error probability (at fixed rate below capacity) the analysis of the fundamental limits has gone through three distinct phases. No information measures were involved during the first phase and any optimization with respect to various auxiliary parameters and input distribution had to rely on standard convex optimization techniques, such as Karush-Kuhn-Tucker conditions, which not only are cumbersome to solve in this particular setting, but shed little light on the structure of the solution. The second phase firmly anchored the problem in a large deviations foundation, with the fundamental limits expressed in terms of conditional relative entropy as well as mutual information. Unfortunately, the associated maximinimization in (2) did not immediately lend itself to analytical progress. Thanks to Csiszár’s realization of the relevance of Rényi’s information measures to this problem, the third phase has found a way to, not only express the error exponent functions as a function of information measures, but to solve the associated optimization problems in a systematic way. While, in the absence of cost constraints, the problem reduces to finding the maximal α-mutual information, cost constraints make the problem much more challenging because of the difficulty in determining the order-α Augustin–Csiszár mutual information. Fortunately, thanks to the introduction of an auxiliary input distribution (the 〈α〉-adjunct of the distribution that maximizes $I_{\alpha }^{c}$), we have shown that α-mutual information also comes to the rescue in the maximization of the order-α Augustin–Csiszár mutual information in the presence of average cost constraints. We have also finally ended the isolation of Gallager’s $E_{0}$ function with cost constraints from the representations in Phases 2 and 3. The pursuit of such a link is what motivated Augustin in 1978 to define a generalized mutual information measure. Overall, the analysis has given yet another instance of the benefits of variational representations of information measures, leading to solutions based on saddle points. However, we have steered clear of off-the-shelf minimax theorems and their associated topological constraints.
  We have worked out two channels/cost constraints (additive Gaussian noise with quadratic cost, and additive exponential noise with a linear cost) that admit closed-form error-exponent functions, most easily expressed in parametric form. Furthermore, in Items 77 and 80 we have illuminated the structure of those closed-form expressions by identifying the anomalous channel behavior responsible for most errors at every given rate. In the exponential noise case, the solution is simply a noisier exponential channel, while in the Gaussian case it is the result of both a noisier Gaussian channel and an attenuated input.
  These observations prompt the question of whether there might be an alternative general approach that eschews Rényi’s information measures to arrive at not only the most likely anomalous channel behavior, but the error exponent functions themselves.

## Data Availability

Not applicable.

## Appendix A

Recall that the relative information ${ı}_{P∥Q}$ is defined only if P≪Q, while D(P∥Q)∈[0,+∞] is always defined and equal to +∞ if (but not only if) *P*  *Q*.

**Lemma** **A1.**
*If Q≪R and X∼P≪R, then*

(A1) $$\begin{array}{c} E\left[{ı}_{P∥R}\left(X\right)-{ı}_{Q∥R}\left(X\right)\right]=D\left(P\;∥\;Q\right), \end{array}$$

*regardless of whether the right side is finite.*

**Proof.** If P≪Q≪R, we may invoke the chain rule (7) to decompose

(A2) $$\begin{array}{c} {ı}_{P∥R}\left(a\right)-{ı}_{Q∥R}\left(a\right)={ı}_{P∥Q}\left(a\right). \end{array}$$

Then, the result follows by taking expectations of (A2) when a←X∼P. To show that (A1) also holds when *P*  *Q*, i.e., that the expectation on the left side is +∞, we invoke the Lebesgue decomposition theorem (e.g. p. 384 of [74]), which ensures that we can find α∈[0,1), $P_{0}⊥Q$ and $P_{1}\ll Q$, such that

(A3) $$\begin{array}{c} P=\alpha \;P_{1}+\left(1-\alpha \right)P_{0}. \end{array}$$

Since $P_{1}⊥P_{0}$, we have

(A4) $$\begin{array}{cc} D(P_{1}\;∥\;P) & =\log\frac{1}{\alpha }, \end{array}$$

(A5) $$\begin{array}{cc} D(P_{0}\;∥\;P) & =\log\frac{1}{1-\alpha }. \end{array}$$

If $X_{1}∼P_{1}$, then

(A6) $$\begin{array}{cc} E\left[{ı}_{P∥R}\left(X_{1}\right)-{ı}_{Q∥R}\left(X_{1}\right)\right]\; & =E\left[{ı}_{P_{1}∥R}\left(X_{1}\right)-{ı}_{Q∥R}\left(X_{1}\right)\right]-E\left[{ı}_{P_{1}∥R}\left(X_{1}\right)-{ı}_{P∥R}\left(X_{1}\right)\right] \end{array}$$

(A7) $$\begin{array}{cc} \; & =D\left(P_{1}\;∥\;Q\right)-D\left(P_{1}\;∥\;P\right) \end{array}$$

(A8) $$\begin{array}{cc} \; & =D\left(P_{1}\;∥\;Q\right)-\log\frac{1}{\alpha }, \end{array}$$

where

- (A7) ⟸ (A1) with $\left(P,Q,R\right)\leftarrow \left(P_{1},Q,R\right)$ and (A1) with $\left(P,Q,R\right)\leftarrow \left(P_{1},P,R\right)$, which we are entitled to invoke since $P_{1}$ is dominated by both *Q* and *R*;
- (A8) ⟸ (A4).

Analogously, if $X_{0}∼P_{0}$, then

(A9) $$\begin{array}{cc} E\left[{ı}_{P∥R}\left(X_{0}\right)\right]\; & =E\left[{ı}_{P_{0}∥R}\left(X_{0}\right)\right]-E\left[{ı}_{P_{0}∥R}\left(X_{0}\right)-{ı}_{P∥R}\left(X_{0}\right)\right] \end{array}$$

(A10) $$\begin{array}{cc} \; & =D\left(P_{0}\;∥\;R\right)-D\left(P_{0}\;∥\;P\right) \end{array}$$

(A11) $$\begin{array}{cc} \; & =D\left(P_{0}\;∥\;R\right)-\log\frac{1}{1-\alpha }. \end{array}$$

Therefore, we are ready to conclude that

$$\begin{array}{cc} \; & \;E\left[{ı}_{P∥R}\left(X\right)-{ı}_{Q∥R}\left(X\right)\right] \end{array}$$

(A12) $$\begin{array}{c} =\alpha \;E\left[{ı}_{P∥R}\left(X_{1}\right)-{ı}_{Q∥R}\left(X_{1}\right)\right]+\left(1-\alpha \right)\;E\left[{ı}_{P∥R}\left(X_{0}\right)-{ı}_{Q∥R}\left(X_{0}\right)\right] \end{array}$$

(A13) $$\begin{array}{cc} \; & =\alpha \;D\left(P_{1}\;∥\;Q\right)+\left(1-\alpha \right)D\left(P_{0}\;∥\;R\right)-\left(1-\alpha \right)E\left[{ı}_{Q∥R}\left(X_{0}\right)\right]-h\left(\alpha \right) \end{array}$$

(A14) $$\begin{array}{cc} \; & =+\infty , \end{array}$$

where

- (A12) ⟸ (A3);
- (A13) ⟸h(·) is the binary entropy function, (A8) and (A11);
- (A14) ⟸$E\left[{ı}_{Q∥R}\left(X_{0}\right)\right]=-\infty$⟸$P_{0}\left(x\in A:\frac{dQ}{dR}\left(x\right)=0\right)=1$⟸$P_{0}⊥Q$.

□

**Corollary** **A1.**
*Suppose that Q≪R and X∼P≪R. Then,*

(A15) $$\begin{array}{c} E\left[{ı}_{Q∥R}\left(X\right)\right]=D\left(P\;∥\;R\right)-D\left(P\;∥\;Q\right), \end{array}$$

*as long as at least one of the relative entropies on the right side is finite.*

## References

[1] Shannon C.E. (1948). A Mathematical Theory of Communication. Bell Syst. Tech. J..

[2] Rice S.O. (1950). Communication in the Presence of Noise–Probability of Error for Two Encoding Schemes. Bell Syst. Tech. J..

[3] Shannon C.E. (1959). Probability of Error for Optimal Codes in a Gaussian Channel. Bell Syst. Tech. J..

[4] Elias P. (1955). Coding for Noisy Channels. IRE Conv. Rec..

[5] Feinstein A. (1955). Error Bounds in Noisy Channels without Memory. IRE Trans. Inf. Theory.

[6] Shannon C.E. (1957). Certain Results in Coding Theory for Noisy Channels. Inf. Control.

[7] Fano R.M. (1961). Transmission of Information.

[8] Gallager R.G. (1965). A Simple Derivation of the Coding Theorem and Some Applications. IEEE Trans. Inf. Theory.

[9] Gallager R.G. (1968). Information Theory and Reliable Communication.

[10] Shannon C.E., Gallager R.G., Berlekamp E. (1967). Lower Bounds to Error Probability for Coding on Discrete Memoryless Channels, I. Inf. Control.

[11] Shannon C.E., Gallager R.G., Berlekamp E. (1967). Lower Bounds to Error Probability for Coding on Discrete Memoryless Channels, II. Inf. Control.

[12] Dobrushin R.L. (1962). Asymptotic Estimates of the Error Probability for Transmission of Messages over a Discrete Memoryless Communication Channel with a Symmetric Transition Probability Matrix. Theory Probab. Appl..

[13] Dobrushin R.L. (1962). Optimal Binary Codes for Low Rates of Information Transmission. Theory Probab. Appl..

[14] Kullback S., Leibler R.A. (1951). On Information and Sufficiency. Ann. Math. Stat..

[15] Csiszár I., Körner J. (1981). Graph Decomposition: A New Key to Coding Theorems. IEEE Trans. Inf. Theory.

[16] Barg A., Forney G.D. (2002). Random codes: Minimum Distances and Error Exponents. IEEE Trans. Inf. Theory.

[17] Sason I., Shamai S. (2006). Performance Analysis of Linear Codes under Maximum-likelihood Decoding: A Tutorial. Found. Trends Commun. Inf. Theory.

[18] Ashikhmin A.E., Barg A., Litsyn S.N. (2000). A New Upper Bound on the Reliability Function of the Gaussian Channel. IEEE Trans. Inf. Theory.

[19] Haroutunian E.A., Haroutunian M.E., Harutyunyan A.N. (2007). Reliability Criteria in Information Theory and in Statistical Hypothesis Testing. Found. Trends Commun. Inf. Theory.

[20] Scarlett J., Peng L., Merhav N., Martinez A., Guillén i Fàbregas A. (2014). Expurgated Random-coding Ensembles: Exponents, Refinements, and Connections. IEEE Trans. Inf. Theory.

[21] Somekh-Baruch A., Scarlett J., Guillén i Fàbregas A. A Recursive Cost-Constrained Construction that Attains the Expurgated Exponent. Proceedings of the 2019 IEEE International Symposium on Information Theory.

[22] Haroutunian E.A. (1968). Estimates of the Exponent of the Error Probability for a Semicontinuous Memoryless Channel. Probl. Inf. Transm..

[23] Blahut R.E. (1974). Hypothesis Testing and Information Theory. IEEE Trans. Inf. Theory.

[24] Csiszár I., Körner J. (1981). Information Theory: Coding Theorems for Discrete Memoryless Systems.

[25] Rényi A., Neyman J. (1961). On Measures of Information and Entropy. Berkeley Symposium on Mathematical Statistics and Probability.

[26] Campbell L.L. (1965). A Coding Theorem and Rényi’s Entropy. Inf. Control.

[27] Arimoto S. (1975). Information Measures and Capacity of Order *α* for Discrete Memoryless Channels. Topics in Information Theory.

[28] Sason I., Verdú S. (2018). Arimoto-Rényi conditional entropy and Bayesian *M*-ary hypothesis testing. IEEE Trans. Inf. Theory.

[29] Fano R.M. (1953). Class Notes for Course 6.574: Statistical Theory of Information.

[30] Csiszár I. (1972). A Class of Measures of Informativity of Observation Channels. Period. Mat. Hung..

[31] Sibson R. (1969). Information Radius. Z. Wahrscheinlichkeitstheorie Und Verw. Geb..

[32] Csiszár I. (1995). Generalized Cutoff Rates and Rényi’s Information Measures. IEEE Trans. Inf. Theory.

[33] Arimoto S. (1976). Computation of Random Coding Exponent Functions. IEEE Trans. Inf. Theory.

[34] Candan C. (2020). Chebyshev Center Computation on Probability Simplex with *α*-divergence Measure. IEEE Signal Process. Lett..

[35] Poltyrev G.S. (1982). Random Coding Bounds for Discrete Memoryless Channels. Probl. Inf. Transm..

[36] Augustin U. (1978). Noisy Channels. Ph.D. Thesis.

[37] Tomamichel M., Hayashi M. (2018). Operational Interpretation of Rényi Information Measures via Composite Hypothesis Testing against Product and Markov Distributions. IEEE Trans. Inf. Theory.

[38] Polyanskiy Y., Verdú S. Arimoto Channel Coding Converse and Rényi Divergence. Proceedings of the 48th Annual Allerton Conference on Communication, Control, and Computing.

[39] Shayevitz O. On Rényi Measures and Hypothesis Testing. Proceedings of the 2011 IEEE International Symposium on Information Theory.

[40] Verdú S. *α*-Mutual Information. Proceedings of the 2015 Information Theory and Applications Workshop (ITA).

[41] Ho S.W., Verdú S. Convexity/Concavity of Rényi Entropy and *α*-Mutual Information. Proceedings of the 2015 IEEE International Symposium on Information Theory.

[42] Nakiboglu B. (2019). The Rényi Capacity and Center. IEEE Trans. Inf. Theory.

[43] Nakiboglu B. (2018). The Augustin Capacity and Center. arXiv.

[44] Dalai M. (2017). Some Remarks on Classical and Classical-Quantum Sphere Packing Bounds: Rényi vs. Kullback–Leibler. Entropy.

[45] Cai C., Verdú S. (2019). Conditional Rényi Divergence Saddlepoint and the Maximization of *α*-Mutual Information. Entropy.

[46] Vázquez-Vilar G., Martinez A., Guillén i Fàbregas A. A Derivation of the Cost-constrained Sphere-Packing Exponent. Proceedings of the 2015 IEEE International Symposium on Information Theory.

[47] Wyner A.D. (1988). Capacity and Error Exponent for the Direct Detection Photon Channel. IEEE Trans. Inf. Theory.

[48] Csiszár I., Körner J. (2011). Information Theory: Coding Theorems for Discrete Memoryless Systems.

[49] Rényi A. (1959). On Measures of Dependence. Acta Math. Hung..

[50] van Erven T., Harremoës P. (2014). Rényi Divergence and Kullback-Leibler Divergence. IEEE Trans. Inf. Theory.

[51] Csiszár I., Matúš F. (2003). Information Projections Revisited. IEEE Trans. Inf. Theory.

[52] Csiszár I. (1967). Information-type Measures of Difference of Probability Distributions and Indirect Observations. Stud. Sci. Math. Hung..

[53] Nakiboglu B. (2019). The Sphere Packing Bound via Augustin’s Method. IEEE Trans. Inf. Theory.

[54] Nakiboglu B. (2019). The Augustin Capacity and Center. Probl. Inf. Transm..

[55] Vázquez-Vilar G. (2019). Error Probability Bounds for Gaussian Channels under Maximal and Average Power Constraints. arXiv.

[56] Shannon C.E. (1957). Geometrische Deutung einiger Ergebnisse bei der Berechnung der Kanalkapazität. Nachrichtentechnische Z..

[57] Verdú S., Han T.S. (1994). A General Formula for Channel Capacity. IEEE Trans. Inf. Theory.

[58] Kemperman J.H.B. (1974). On the Shannon Capacity of an Arbitrary Channel. K. Ned. Akad. Van Wet. Indag. Math..

[59] Aubin J.P. (1979). Mathematical Methods of Game and Economic Theory.

[60] Luenberger D.G. (1969). Optimization by Vector Space Methods.

[61] Gastpar M., Rimoldi B., Vetterli M. (2003). To Code, or Not to Code: Lossy Source–Channel Communication Revisited. IEEE Trans. Inf. Theory.

[62] Arimoto S. (1973). On the Converse to the Coding Theorem for Discrete Memoryless Channels. IEEE Trans. Inf. Theory.

[63] Sason I. (2016). On the Rényi Divergence, Joint Range of Relative Entropies, Measures and a Channel Coding Theorem. IEEE Trans. Inf. Theory.

[64] Dalai M., Winter A. (2017). Constant Compositions in the Sphere Packing Bound for Classical-quantum Channels. IEEE Trans. Inf. Theory.

[65] Nakiboglu B. (2020). The Sphere Packing Bound for Memoryless Channels. Probl. Inf. Transm..

[66] Dalai M. (2013). Lower Bounds on the Probability of Error for Classical and Classical-quantum Channels. IEEE Trans. Inf. Theory.

[67] Shannon C.E. (1956). The Zero Error Capacity of a Noisy Channel. IRE Trans. Inf. Theory.

[68] Feder M., Merhav N. (1994). Relations Between Entropy and Error Probability. IEEE Trans. Inf. Theory.

[69] Einarsson G. (1979). Signal Design for the Amplitude-limited Gaussian Channel by Error Bound Optimization. IEEE Trans. Commun..

[70] Anantharam V., Verdú S. (1996). Bits through Queues. IEEE Trans. Inf. Theory.

[71] Verdú S. (1996). The Exponential Distribution in Information Theory. Probl. Inf. Transm..

[72] Arikan E. (1996). On the Reliability Exponent of the Exponential Timing Channel. IEEE Trans. Inf. Theory.

[73] Polyanskiy Y., Poor H.V., Verdú S. (2010). Channel Coding Rate in the Finite Blocklength Regime. IEEE Trans. Inf. Theory.

[74] Royden H.L., Fitzpatrick P. (2010). Real Analysis.

